# Six New Unsymmetrical Imino-1,8-naphthalimide Derivatives Substituted at 3-C Position—Photophysical Investigations

**DOI:** 10.3390/ma15197043

**Published:** 2022-10-10

**Authors:** Sonia Kotowicz, Mateusz Korzec, Jan Grzegorz Małecki, Sylwia Golba, Mariola Siwy, Sebastian Maćkowski, Ewa Schab-Balcerzak

**Affiliations:** 1 Institute of Chemistry, University of Silesia, 9 Szkolna Str., 40-006 Katowice, Poland; 2 Institute of Materials Science, University of Silesia, 41-500 Chorzow, Poland; 3 Centre of Polymer and Carbon Materials, Polish Academy of Sciences, 34 M. Curie-Sklodowska Str., 41-819 Zabrze, Poland; 4 Faculty of Physics, Astronomy and Informatics, Institute of Physics, Nicolaus Copernicus University, 5 Grudziadzka Str., 87-100 Torun, Poland

**Keywords:** 1,8-naphthalimide, electrochemistry, luminescence, imines

## Abstract

In this research, six novel unsymmetrical imino-1,8-naphthalimides (AzNI) were synthesized. Comprehensive thermal (thermogravimetric analysis (TGA) and differential scanning calorimetry (DSC), optical (UV-Vis, photoluminescence), and electrochemical (CV, DPV) studies were carried out to characterize these new compounds. The molecules showed the onset of thermal decomposition in the temperature range 283–372 °C and molecular glass behavior. Imino-1,8-naphthalimides underwent reduction and oxidation processes with the electrochemical energy band gap (E_g_) below 2.41 eV. The optical properties were evaluated in solvents with different polarities and in the solid-state as a thin films and binary blends with poly(*N*-vinylcarbazole): (2-tert-butylphenyl-5-biphenyl-1,3,4-oxadiazole) (PVK:PBD). Presented compounds emitted blue light in the solutions and in the green or violet spectral range in the solid-state. Their ability to emit light under external voltage was examined. The devices with guest-host structure emitted light with the maximum located in the blue to red spectral range of the electroluminescence band (EL) depending on the content of the AzNI in the PVK:PBD matrix (guest-host structure).

## 1. Introduction

The progress and expansion of organic electronics are possible thanks to the synthesis of the new organic semiconductors [1,2], which have been seeing its most significant development since the second half of the 20th century, and is fueled by application of these materials in a light-emitting diodes (OLED) [3,4,5], field-effect transistors (OFET) [6], and photovoltaic cells (PV) [7,8,9]. New organic molecules—both low molecular weight compounds and polymers/oligomers—with specific thermal, redox, and optical properties are still desired, in particular if a synthesis routes are cheap and efficient and are in accord with the green chemistry approach. The organic semiconductors may contain nitrogen-nitrogen (-N=N-), carbon-carbon (-C=C-) or carbon-nitrogen (-C=N-) double bonds and aromatic heterocyclic rings [10]. Azomethinoimides are organic materials with electron-withdrawing properties [11]. Among the n-type semiconductors, the imides have been widely studied as compounds for applications in organic electronics [12,13,14,15,16,17,18]. The ability to self-organize, accompanied by high thermal stability, and chemical and oxidation resistance, render the naphthalimides good acceptor-donor materials [19,20,21,22,23,24]. In addition, the 1,8-naphthalimide derivatives were also used as dyes for cell imaging and as probes or sensors [25,26,27,28,29,30].

In the literature, naphthalimides and their derivatives (substituted at 4- or 3-C position) were applied as active materials in light-emitting diodes. Blue light was registered for the device with the following structure: ITO/PEDOT:PSS/compound/Al based on 4-(2-fenoxi-p-xileno)-*N*-methyl-1,8-naphthalimide [31]. Another example showed that by changing the aliphatic system with the aromatic one in the imide ring and the substitution of the triphenylamine at the 4-C position yields diodes (ITO/PEDOT:PSS/compound/LiF/Al or ITO/PEDOT:PSS/compound/dimethyl-4,7-diphenyl-1,10-phenanthroline (BCP)/LiF/Al) emitting yellowish-green light, whereas the use of the 4,4′-bis(*N*-carbazolyl)-1,1′-biphenyl (CBP) matrix resulted in a shift of the electroluminescence spectrum to the green spectral range [32]. The red light induced by external voltage was observed for the device with the structure of ITO/MoO_3_/4,4′,4′′-tri(*N*-carbazolyl)triphenylamine/compund:matrix/2,2′,2′′-(1,3,5-benzinetriyl)-tris(1-phenyl-1-*H*-benzimidazole) (TPBI)/LiF/Al, demonstrating thus for the first time the possibility of using 1,8-naphthalimide derivative as red emitters [33]. The electroluminescence ability of the unsymmetrical azomethinoimides was investigated in a diode with the structure of ITO/PVK:compound/Al and in this case the emission of green light was observed [34]. The electroluminescence properties of a device ITO/PEDOT:PSS/PVK:compound/TPBI/LiF/Al based on the divinylene 1,8-naphthalimide derivatives with fluorene or phenylene core were also investigated. Such diodes emitted green or orange light [35]. The obtained materials with the possibility of color tunability, maximizing at the same time the external quantum efficiency (EQE), and the stability of the device operation is still a topic of current research [16].

In this work, six new azomethino-*N*-hexyl-1,8-naphthalimide derivatives are presented. The influence of the substituent in the 3-C position on thermal, photophysical, and electrochemical properties, including the ability to generate electroluminescence, is discussed. The experimental results were supported by the DFT calculations. Additionally, the properties of two compounds, AzNI-5 and AzNI-6, are discussed in comparison with previously reported symmetrical analogues (with two 1,8-naphthalimide group) [36]. The obtained results may be helpful to design new materials with specific properties, for developing new materials in organic optoelectronics, or as pretenders to biological imaging. The presented results are the result of extensive research on the 1,8-naphthalimide compounds substituted in the 3-C position with the imine linker.

## 2. Experimental Section

### 2.1. Materials and Characterization Methods

Based on the publication [34], 3-amino-*N*-hexyl-1,8-naphthalimide was synthesized. Characterization methods, materials, films, blends, and OLEDs preparations are described in ESI (Supplementary Materials).

### 2.2. Synthesis Description

Previously synthesized 3-amino-N-hexyl-1,8-naphthalimide (m = 0.296 g, 1 mmol) was dissolved in 10 cm^3^ of methanol and to the dissolved imine the 1 eq of appropriate aldehyde(9-anthracenecarboxaldehyde, 1-naphthaldehyde, 1-pyrenecarboxaldehyde, 4-[(2-cyanoethyl)methylamino]benzaldehyde, 4-phenyl-benzaldehyde, 4-(2-phenyleth-1-ynyl)benzaldehyde) was added. The condensation reaction was carried out for 20 h at room temperature (25 °C ± 1 °C). The obtained powders were filtered, washed with 10 cm^3^ of methanol and dried at 60 °C (±5 °C) over 2 h.

**3-(9-anthracene)-*N*-hexyl-1,8-naphthalimide—AzNI-1**

Yield = 41%. Yellow solid. **^1^H NMR** (400 MHz, DMSO-d_6_, δ, ppm): 10.05 (s, 1H, -CH=N-); 9.01 (d, *J* =8.7 Hz, 2H, -CH); 8.88 (s, 1H, -CH); 8.56 (m, 3H, -CH); 8.50 (d, *J* =7.2 Hz, 1H, -CH); 8.23 (d, *J* =8.3 Hz, 2H, -CH); 7.93 (t, *J* =7.8 Hz, 1H, -CH); 7.93–7.98 (m, 2H, -CH), 7.67–7.61 (m, 2H, -CH); 4.16–4.02 (m, 2H, -N-CH_2_-); 1.76–1.59 (m, 2H, -CH_2_-); 1.44–1.20 (m, 6H, -CH_2_-); 0.88 (t, *J* =6.9 Hz, 3H, -CH_3_). **^13^C NMR** (101 MHz, DMSO-d_6_, δ, ppm): 163.7; 163.6; 162.1; 155.6; 150.8; 140.5; 137.9; 134.6; 132.9; 130.8; 130.3; 130.1; 129.4; 129.2; 129.1; 128.5; 128.2; 126.9; 126.1; 124.9; 124.3; 123.7; 122.5; 31.4; 27.9; 26.6; 22.4; 14.4. **FTIR** (KBr, *v*, cm^−1^): 3052 (C-H aromatic); 2957, 2930 (C-H aliphatic); 1697, 1660 (C=O imide); 1613 (-CH=N- imine). **Anal. Calcd** for C_33_H_28_N_2_O_2_ (484.59 g/mol): C(81.79%) H(5.82%) N(5.78%); found: C(81.79%) H(5.81%) N(5.81%).

**3-(1-naphthalene)-*N*-hexyl-1,8-naphthalimide—AzNI-2**

Yield = 40%. Yellow solid. **^1^H NMR** (400 MHz, DMSO-d_6_, δ, ppm): 9.47 (s, 1H, -CH=N-); 9.35 (d, *J* =8.5 Hz, 1H, -CH); 8.52–8.43 (m, 3H, -CH); 8.42 (d, *J* =2.0 Hz, 1H, -CH); 8.31 (d, *J* =7.2 Hz, 1H, -CH); 8.19 (d, *J* =8.2 Hz, 1H, -CH); 8.09 (d, *J* =7.6 Hz, 1H, -CH); 7.88 (t,*J* =7.7 Hz,1H, -CH); 7.77–7.62 (m, 3H, -CH); 4.12–4.04 (m, 2H, -N-CH_2_-); 1.70–1.62 (m, 2H, -CH_2_-); 1.43–1.25 (m, 6H, -CH_2_-); 0.87 (t, *J* =7.0 Hz, 3H, -CH_3_). **^13^C NMR** (101 MHz, DMSO-d_6_, δ, ppm): 163.9; 163.7; 150.8; 134.7; 134.0; 133.1; 133.0; 131.7; 131.4; 131.2; 130.4; 129.3; 128.3; 127.0; 126.3; 126.0; 125.1; 124.7; 123.7; 122.6; 31.4; 27.9; 26.6; 22.4; 14.4. **FTIR** (KBr, *v*, cm^−1^): 3051 (C-H aromatic); 2930, 2854 (C-H aliphatic); 1697, 1661 (C=O imide); 1623 (-CH=N- imine). **Anal. Calcd** for C_29_H_26_N_2_O_2_ (434.52 g/mol): C(80.16%) H(6.03%) N(6.45%); found: C(80.06%) H(6.05%) N(6.47%).

**3-(1-pyrene)-*N-*hexyl-1,8-naphthalimide—AzNI-3**

Yield = 52%. Yellow solid. **^1^H NMR** (400 MHz, DMSO-d_6_, δ, ppm): 9.91 (s, 1H, -CH=N-); 9.39 (d, *J* =9.4 Hz, 1H, -CH); 8.91 (d, *J* =8.1 Hz, 1H, -CH); 8.61 (m, 1H, -CH); 8.53–8.42 (m, 7H, -CH); 8.37 (d, *J* =8.9 Hz, 1H, -CH); 8.30 (d, *J* =8.9 Hz, 1H, -CH); 8.18 (t, *J* =7.7 Hz, 1H, -CH); 7.91 (t, *J* =7.8 Hz, 1H, -CH); 4.15–4.01 (m, 2H, -N-CH_2_-); 1.75–1.62 (m, 2H, -CH_2_-); 1.42–1.28 (m, 6H, -CH_2_-); 0.88 (t, *J* =6,9 Hz, 3H, -CH_3_).**^13^C NMR** (101 MHz, DMSO-d_6_ or CDCl_3_, δ, ppm): The product was insufficiently soluble for analysis. **FTIR** (KBr, *v*, cm^−1^): 3043 (C-H aromatic); 2952,2853 (C-H aliphatic); 1698, 1661 (C=O imide); 1624 (-CH=N- imine). **Anal. Calcd** for C_35_H_28_N_2_O_2_ (508.61 g/mol): C(82.65%) H(5.55%) N(5.51%); found: C(82.03%) H(5.52%) N(5.42%).

**3-(4-[(2-cyanoethyl)methylamino]-4-benzo)-*N*-hexyl-1,8-naphthalimide—AzNI-4**

Yield = 65%. Yellow solid. **^1^H NMR** (400 MHz, DMSO-d_6_, δ, ppm): 8.70 (s, 1H, -CH=N-); 8.42 (d, *J* =7.1 Hz, 2H, -CH); 8.35 (s, 1H, -CH); 8.21 (s, 1H, -CH); 7.91–7.81 (m, 3H, -CH); 6.93 (d, *J* =8.8 Hz, 2H, -CH); 4.13–4.02 (m, 2H, -N-CH_2_-);3.81 (t, *J* =6.5 Hz, 2H, -N-CH_2_-); 3.08 (s, 3H, -N-CH_3_); 2.80 (t, *J* =6.6 Hz, 2H, -CH_2_-); 1.74–1.57 (m, 2H,-CH_2_-); 1.41–1.26 (m, 6H, -CH_2_-); 0.88 (t, *J* =6.6 Hz, 3H, -CH_3_). **^13^C NMR** (101 MHz, DMSO-d_6_, δ, ppm): 163.9; 163.8; 162.5; 151.6; 151.4; 134.3; 133.1; 131.3; 129.8; 127.9; 125.9; 125.9; 124.9; 124.1; 123.6; 122.6; 119.7; 112.36; 47.9; 38.4; 31.3; 27.9; 26.6; 22.4; 15.6; 14.2. **FTIR** (KBr, *v*, cm^−1^): 3078 (C-H aromatic); 2954, 2858 (C-H aliphatic); 2246 (C≡C); 1697,1656 (C=O imide); 1629 (-CH=N- imine). **Anal. Calcd** for C_29_H_30_N_4_O_2_ (466.57 g/mol): C(74.65%) H(6.48%) N(12.01%); found: C(74.33%) H(6.45%) N(11.94%).

**3-(4-phenyl-benzo)-*N*-hexyl-1,8-naphthalimide—AzNI-5**

Yield = 49%. Yellow solid. **^1^H NMR** (400 MHz, DMSO-d_6_, δ, ppm): 8.93 (s, 1H, -CH=N-); 8.47–8.39 (m, 3H, -CH); 8.30 (d, *J* =2.0 Hz, 1H, -CH); 8.13 (d, *J* =8.3 Hz, 2H, -CH); 7.91–7.85 (m, 3H, -CH); 7.78 (d, *J* =7.3 Hz, 2H, -CH); 7.52 (t, *J* =7.5 Hz, 2H, -CH); 7.44 (t, *J* =7.3 Hz, 1H, -CH); 4.06 (t, *J* =7.3 Hz, 2H, -N-CH_2_-); 1.69–1.59 (m, 2H, -CH_2_-); 1.40–1.25 (m, 6H, -CH_2_-); 0.87 (t, *J* =6.9 Hz, 3H, -CH_3_). **^13^C NMR** (101 MHz, DMSO-d_6_, δ, ppm): The product was insufficiently soluble for analysis. **^1^H NMR** (400 MHz, CDCl_3_, δ, ppm): 8.71 (s, 1H, -CH=N-); 8.55 (d, *J* =5.8 Hz, 2H, -CH); 8.21 (d, *J* =8.2 Hz, 1H-CH);8.06 (d, *J* =7.9 Hz, 2H, -CH);8.00 (s, 1H, -CH); 7.77 (d, *J* =7.9 Hz, 3H, -CH); 7.69 (d, *J* =7.7 Hz, 2H, -CH); 7.51 (t, *J* =7.3 Hz, 2H, -CH); 7.43 (t, *J* =7.2 Hz, 1H, -CH); 7.28 (s, 1H,-CH); 4.20–4.15 (m, 2H, -N-CH_2_-);1.85–1.70 (m, 2H, -CH_2_-); 1.53–1.29 (m, 6H, -CH_2_-); 0.96–0.95 (m, 3H, -CH_3_). **^13^C NMR** (101 MHz, CDCl_3_, δ, ppm): 164.1; 164.0; 161.9; 150.0; 144.8; 140.1; 134.7; 133.6; 132.0; 130.3; 129.7; 129.0; 128.1; 127.0; 127.6; 127.4; 127.4; 127.2; 126.5; 125.1; 124.7; 123.9; 122.8; 113.9; 40.6; 31.6; 28.1; 26.8; 22.6; 14.1. **FTIR** (KBr, *v*, cm^−1^): 3059 (C-H aromatic); 2929, 2857 (C-H aliphatic); 1699, 1658 (C=O imide); 1631 (-CH=N- imine). **Anal. Calcd** for C_31_H_28_N_2_O_2_ (460.56 g/mol): C, 80.84; H, 6.13; N, 6.08; found: C, 80.79; H, 6.11; N, 6.08.

**3-(4-(2-phenyleth-1-ynyl)benzo)-*N*-hexyl-1,8-naphthalimide—AzNI-6**

Yield = 55%. Yellow solid. **^1^H NMR** (400 MHz, DMSO-d_6_, δ, ppm): 8.95 (s, 1H, -CH=N-); 8.48–8.40 (m, 3H, -CH); 8.33 (d, *J* =2.0 Hz, 1H, -CH); 8.10 (d, *J* =8.2 Hz, 2H, -CH); 7.89 (t, *J* =7.7 Hz, 1H, -CH); 7.76 (d, *J* =8.2 Hz, 2H, -CH); 7.66–7.56 (m, 2H, -CH); 7.53–7.41 (m, 3H, -CH); 4.12–4.01 (m, 2H, -N-CH_2_-); 1.74–1.58 (m, 2H, -CH_2_-); 1.43–1.22 (m, 6H, -CH_2_-); 0.88 (t, *J* =6.8 Hz, 3H, -CH_3_). **^13^C NMR** (101 MHz, DMSO-d_6_, δ, ppm): 163.3; 163.7; 162.7; 150.2; 136.2; 134.6; 132.9; 132.3; 131.9; 130.4; 129.6; 129.3; 128.2; 126.4; 126.2; 125.5; 124.9; 123.8; 122.7; 122.5; 92.4; 89.5; 31.4; 27.9; 26.6; 22.4; 14.22. **FTIR** (KBr, *v*, cm^−1^): 3050 (C-H aromatic); 2953, 2929 (C-H aliphatic); 2213 (C≡C); 1699, 1655 (C=O imide); 1654 (-CH=N- imine). **Anal. Calcd** for C_33_H_28_N_2_O_2_ (484.59 g/mol): C, 81.79; H, 5.82; N, 5.78; found: C, 81.32; H, 5.68; N, 5.93.

## 3. Result and Discussion

### 3.1. Structural Characterization

**Figure 1:**
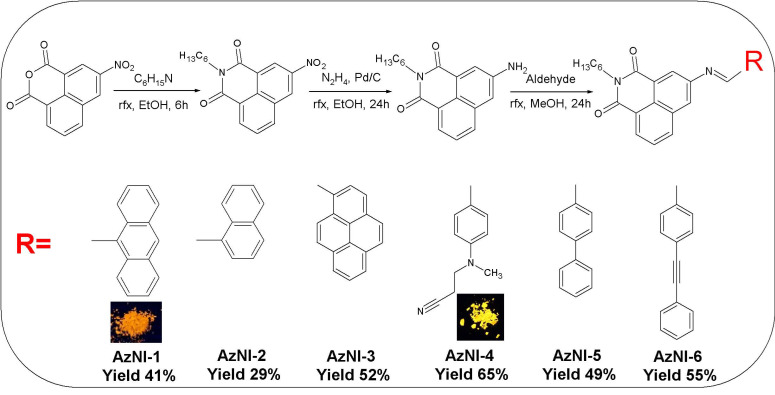
The synthetic route and chemical structure of *N*-hexyl-1,8-naphthalimide derivatives Insert: photos of the selected compounds under UV-light with λ_ex_ = 366 nm.

The new imino-1,8-naphthalimides (AzNIs) derivatives were synthesized in eco-friendly conditions by condensation reaction. AzNIs with imine bond were obtained as yellow solids, and for two of them, the photos under UV-light (excitation wavelength of 366 nm) were presented (Figure S2 in Supplementary Material). The imino-1,8-naphthalimides are substituted at 3-C position via imine linkage with the naphthalene ring with a 9-anthracene (AzNI-1), 1-naphthalene (AzNI-2), 1-pyrene (AzNI-3), (2-cyanoethyl)methylamino-4-benzyle (AzNI-4), 4-phenyl-benzene (AzNI-5) and 4-(2-phenyleth-1-ynyl)benzene (AzNI-6). The structural formula and synthetic route of the targeted compounds is presented in Figure 1. The ^1^H NMR spectra were registered (Figure S1) and exhibit the proton of the –C=N– group as a singlet in the range of 8.70–10.05 ppm with a shift toward higher ppm for AzNI-10 with anthracene substituent. The proton signals of the aromatic rings were seen in the range characteristic for these compounds. The proton signals of the -CH_3_ and -CH_2_- groups of the (2-cyanoethyl)methylamine substituent (compound AzNI-4) were registered at 2.80 ppm, 3.08 ppm, and 3.81 ppm, respectively. In the case of compounds with anthracene (AzNI-1) and naphthalene (AzNI-2), the signal of the imine proton was weaker shielded for the compound with an anthracene substituent. Additionally, in the FTIR spectra, the absorption band of the imine bond was located at lower frequencies for AzNI-1, which may indicate a better degree of conjugation for this compound [37]. The absorption band observed in the range of 1613–1654 cm^−1^ in the FTIR spectra was derived from the stretching vibration of the –C=N– group. Presence of the –C≡C– bond vibration in the AzNI-6 compound was noted at 2213 cm^−1^, and absorption band of the stretching vibrations of the –C≡N bond in the AzNI-4 compound was noted at 2246 cm^−1^.

Absorption bands of the stretching vibrations of –C=O bonds in the imide ring were observed in the range of 1655–1699 cm^−1^ and bands of aliphatic groups in the range 2854–28,957 cm^−1^. The elemental analysis was also performed. Good agreement of the nitrogen, carbon, and hydrogen atoms with the theoretical values was found.

**Figure 2:**
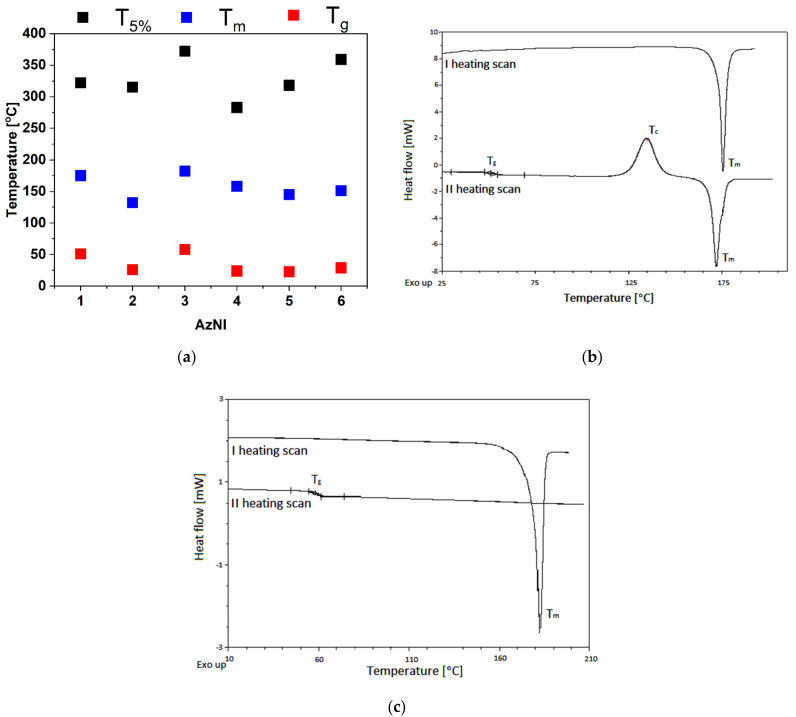
(**a**) Collected data from the DSC TGA investigations and (**b**) DSC thermograms of AzNI-1 and (**c**) AzNI-3.

The thermogravimetric analysis (TGA) and differential scanning calorimetry (DSC) were used to thermal investigations under nitrogen. Thermal stability was defined using TGA, determining the temperature of 5% weight loss (T_5_) of the sample during dynamic heating and the temperature of the maximum decomposition rate (T_max_) from the differential curve (DTG). In addition, the percentage of the sample residue after heating to 600 °C was given (Table 1). The phase transition temperatures (T_m_, T_c_) and the glass transition temperature (T_g_) were determined by DSC (heating/cooling rate of 20 °C·min^−1^ under nitrogen). The data from thermal analysis are collected in Table 1 and in Figure 2.

The T_5_ was obtained in the range of 283–372 °C (Table 1, Figure 2a and Supplementary Figure S3a). The highest T_5_ was seen for azomethinoimide with a pyrene substituent (AzNI-3). Azomethinoimides after synthesis were obtained as crystalline materials with a melting temperature in the range of 132–182 °C. Presented imines were molecular glasses with the glass transition temperature (T_g_, registered after rapid cooling in the second heating scan). In the second heating scan, the glass transition temperature, “cold crystallization temperature” and melting temperature were observed (Figure 2b), except for AzNI-3 and AzNI-5. These molecules can form a stable amorphous phase, without crystallization and melting during heating above T_g_ (Figure 2c). The T_m_ and T_g_ temperatures were observed in line with the trend for substituents: naphthalene < phenanthrene < anthracene < pyrene (a compound with naphthalene substituent described in our former work [38]). The presence of ethynyl bond (AzNI-6) increased the T_5%_, T_m_ and T_g_ compared with the molecule bearing 4-phenyl-benzyle unit (AzNI-5). It can be interesting to compare AzNI-5 and AzNI-6 with its reported symmetrical analogues [36]. The symmetrical azomethino-1,8-naphthalimides showed higher T_5_, T_m_ and T_g_ temperatures compared with AzNI-5 and AzNI-6, with one exception. The T_5_ was higher for AzNI-6 (T_5_ = 359 °C) than for his symmetrical analog (T_5_ = 285 °C).

### 3.2. Redox Properties

**Figure 3:**
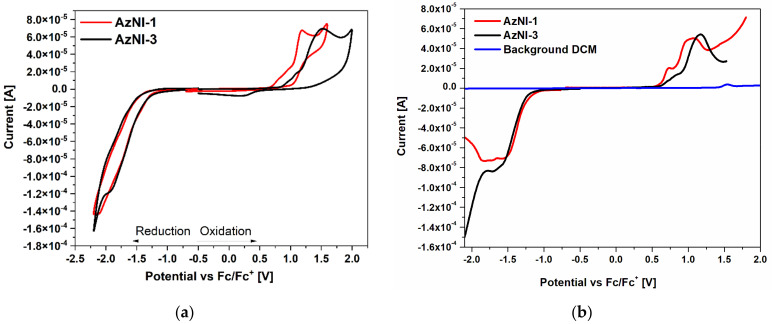
(**a**) Cyclic voltammetry (CV) and (**b**) differential pulse voltammetry (DPV) scans of AzNI-1 and AzNI-3 in positive and negative potential range (v = 0.1 V/s for CV and = 0.05 V/s for DPV, electrolyte 0.1 M Bu_4_NPF_6_ in CH_2_Cl_2_ with Pt as a working electrode).

The electrochemical investigations were performed in dichloromethane (CH_2_Cl_2_) and dry acetonitrile (ACN) solution with three component cells (with platinum electrode (Pt) as a working electrode) using two electrochemical methods: cyclic voltammetry (CV) and differential pulse voltammetry (DPV). The experimentally determined onset of the oxidation and reduction peaks were used for the calculations of the ionization potentials (IP) and electron affinities (EA), which correspond to the HOMO and LUMO energy levels. The ionization potentials value of ferrocene (Fc) was equal to −5.1 eV as provided by findings of P. Bujak, et al. [39]. The electrochemical data based on the dichloromethane investigation are collected in Table 2 and based on the dry ACN in Table S7, and representative voltammograms are presented in Figure 3 and Supplementary Figure S13.

For all compounds, two distinct processes induced by an external voltage are visible. They relate to both the oxidation act (in the positive potential range) and to the reduction act (in the negative potential range). The first process is related to the oxidation of the side substituent part, while in the reduction mainly imine part of the molecule is involved [40]. In the positive potential range (the potential values provided in reference to the mentioned internal standard, namely vs Fc/Fc^+^ redox couple) anodic peak of irreversible nature is formed as the electron is withdrawn from the molecule’s structure. The lowest E_ox_ value was found in two investigated solutions for AzNI–4 substituted with 4-[(2-cyanoethyl)methylamino]-4-benzo group, which may be related with free electron pair of nitrogen that can be subtracted in the oxidation act. On the other end of the oxidation proneness queue, there is AzNI–3 with a pyrene ring characterized by the highest oxidation potential value as a marker of reluctance of the molecule to lose the electron (Figure 3). The increase in number of aromatic rings in the molecule’s structure is related to the higher oxidation potential as seen in the case of samples where E_ox_^1(onset)^ is increasing in the order AzNI–4 < AzNI–5 (biphenyl substituent) < AzNI–6 (4-(2-phenyleth-1-ynyl)benzene substituent). Furthermore, the spatial separation of the neighboring phenyl rings also provides an increase in the E_ox_ onset value.

As the system is polarized in the other direction, one may find the reduction act with the reduction onset potential as low as at −1.75 V for AzNI–2 in CH_2_Cl_2_ (−1.60 V in ACN)spanning to the −1.26 V for AzNI–1 in CH_2_Cl_2_ and −1.42 V for AzNI-4 in ACN (for CV measurements). It seems plausible that in the case of AzNI–2 the 1-naphthalene—substituent lowers E_red_^1(onset)^ value, while the 9-anthracene and 4-[(2-cyanoethyl)methylamino]-4-benzo substituent higher this value. The greater number of aromatic rings in the molecule’s structure leads to the lower reductive potential as seen in the case of samples where E_red_^(onset)^ is increasing in the order AzNI–5 < AzNI–6 < AzNI–4 (in CH_2_Cl_2_).

The electrochemical energy band gaps (E_g_) calculated based on the E_HOMO_ and E_LUMO_ values were in the range of 1.92–2.41 eV (values derived from CV in CH_2_Cl_2_, 1.87–2.36 eV in ACN). The lowest E_g_ value was found for azomethine with anthracene substituent (AzNI–1) (1.92 eV), followed by AzNI–4 (1.97 eV), AzNI–3 (2.14 eV), AzNI–6 (2.25 eV), AzNI–5 (2.28 eV) and AzNI–2 (2.41 eV) in dichloromethane solution. In dry acetonitrile the lowest E_g_ value was found for azomethine AzNI-4 (1.87 eV) and the highest for AzNI-3 (2.36 eV) (Table S7). The changes are connected with shifts of E_LUMO_ value and E_HOMO_ value presenting interplay between molecules dual ability to deliver and take electrons. The lowest optical band gap (E_g_^opt^) in three solutions was also observed for AzNI-1 (2.53–2.61 eV) (Table S6). However, the highest optical band gap was seen for AzNI-6 and AzNI-5, with very little values difference between them (3.01–3.07 eV).

The previously described symmetrical azomethino-1,8-naphthalimides [36] demonstrated an easier reduction process (with lower potential) than their unsymmetrical counterparts presented in this work (analogues to compounds with biphenyl (AzNI-5) and 4-(2-phenyleth-1-ynyl)benzene (AzNI-6)). The lower value of the reduction potential influenced the position of E_LUMO_, lowering it and thus reducing the value of the energy band gap.

### 3.3. DFT Calculations

**Figure 4:**
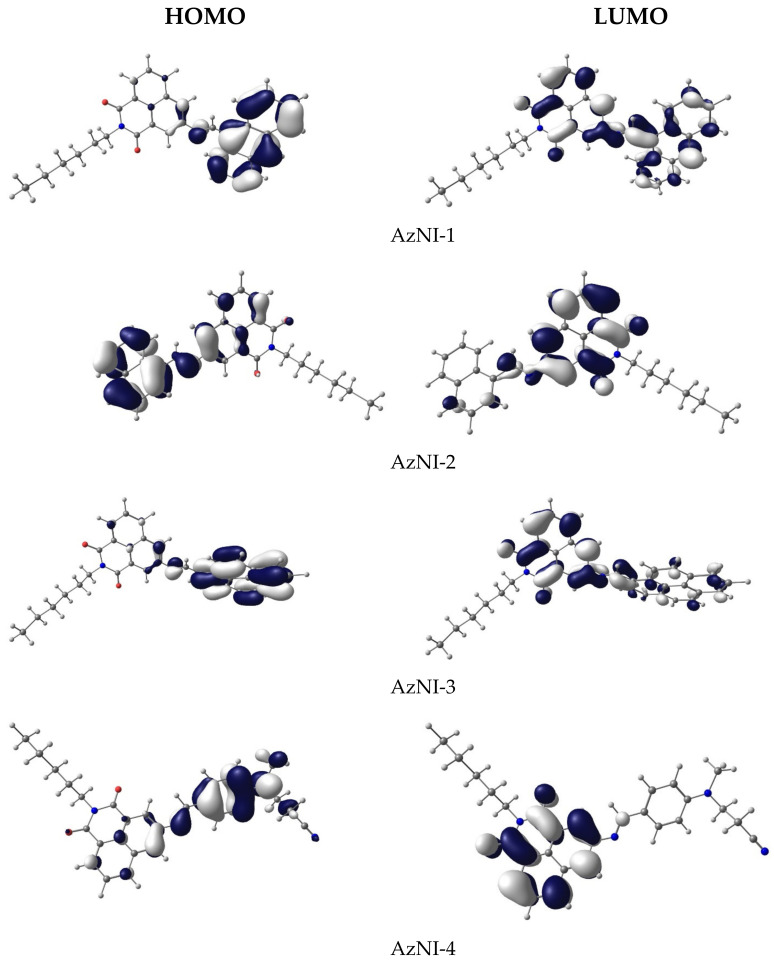

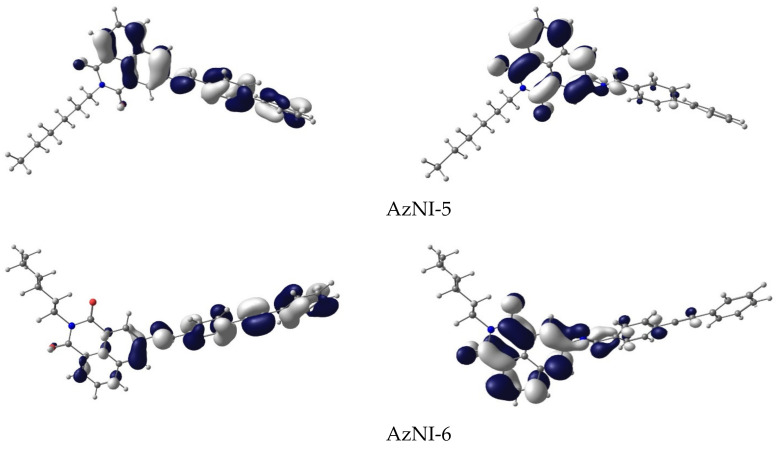
Contours of HOMO and LUMO energy levels of studied compounds.

Theoretical calculations were calculated using the Gaussian09 program (C.01, Gaussian, Inc., Wallingford CT, USA, 2019) and the calculation details are given in the Supplementary Materials (Figure S4 and S5, Table S1–S4). Molecular geometry of the singlet (S) ground state of the compounds was optimized in the gas phase on the B3LYP/6-31g^++^ level of theory augmented with GD3BJ dispersion correction model. A frequency calculation for the compounds was carried out, verifying that the optimized molecular structure corresponds to energy minimum (only positive frequencies were expected). The calculations were carried out for analysis of the HOMO, LUMO energy levels and UV-Vis and photoluminescence data. Optimized geometries and contours of the molecular energy orbitals are presented in Figure 4 and Supplementary Figure S4 in Supplementary Material.

The calculated HOMO energies do not vary much from the experimental values (cf. Table 2). The largest difference between calculated and experimental values occurs in the compound with 4-phenyl-benzene (AzNI-5, ∆ = 0.32 eV). In the case of LUMO energies, the energy differences are much higher and on average around 1 eV, because the virtual orbitals generally are more difficult to describe theoretically. However, the calculated HOMO and LUMO energies were used only for consistency with geometry optimization. To describe the molecular orbitals in detail, the contribution of the parts of the molecules to a molecular orbital was calculated, i.e., 1,8-napthalimide with aliphatic (–C_6_H_13_) chain, imine (–HC=N–) and substituent fragments (R = 9-anthracene, 1-naphthalene, 1-pyrene, (2-cyanoethyl)methylamine, biphenyl and 4-(2-phenyleth-1-ynyl)benzene). The obtained DOS diagrams are presented in Figure S5 and compositions of selected molecular orbitals in the ground state are gathered in Table S1. Electronic structures of these compounds are similar and so are the HOMO orbitals, except for AzNI-5, in which HOMO is localized on both aromatic parts, while LUMO mainly comprises the 1,8-naphtalimide part. Additionally, the case of AzNI-1 LUMO includes the π antibonding orbitals of conjugate bonds (cf. Figure 4 and Supplementary Figure S5 and Supplementary Table S1).

In the S_0_ state, the compounds display the deviation from planarity and the mean plane angles between 1,8-napthalimide and substituent (R) range from about 30° (AzNI-1) to 50° (Table S2 in Supplementary Material). All molecules are polar and the values of the calculated dipole moment in chloroform solution range from 5.97 D for AzNI-4 to 8.01 D for AzNI-3. The dipole moments of the imines in S (singlet) and T (triplet) excited states are higher than in the ground state. In the excited states, the geometry of the molecules flattens compared to the ground state, although in the case of AzNI-1 the changes in geometry are relatively small.

**Figure 5:**
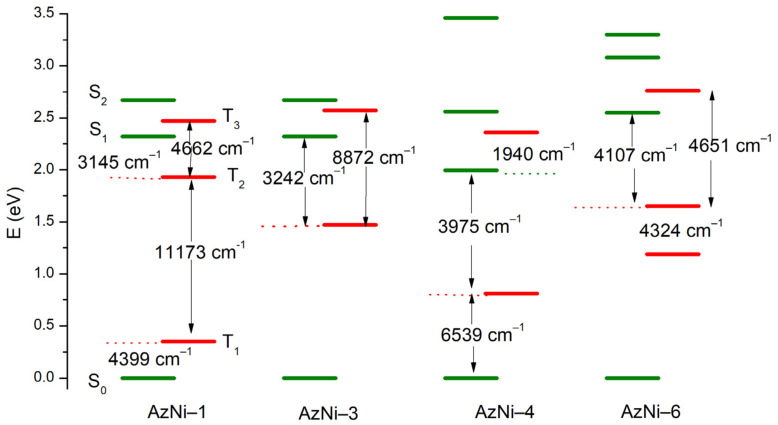
Low-lying energy states in AzNI-1, AzNI-3, AzNI-4, and AzNI-6 molecules.

According to the TD-DFT calculations, the excitation wavelengths resulting in emission (*vide infra*) have a hybrid nature: locally excited/intra molecular charge transfer (LE/ICT) (Table S3). These compounds exhibit photoluminescence with low quantum yields and TD-DFT method was used to optimize singlet and triplet excited states in chloroform as solvent. Calculated energy differences between the ground and the first singlet excited state of AzNI-1, AzNI-3, and AzNI-4 agree well with the experimental values of emission maxima (Table S4). The deactivation as a result of the internal energy conversion is highly possible because of the relatively small differences in energies of the lowest singlet and triplet excited states (Figure 5 and Table S4). Furthermore, the disagreement between S_1_ and T_1_ excited states correspond to the frequencies of the vibrational modes in aliphatic –C_6_H_13_ chain and aromatic C–H bonds in the molecules, the energy of these states may be dispersed as a result of the oscillations within the molecules.

### 3.4. Optical Investigations

#### 3.4.1. UV-Vis Absorption and Emission

**Figure 6:**
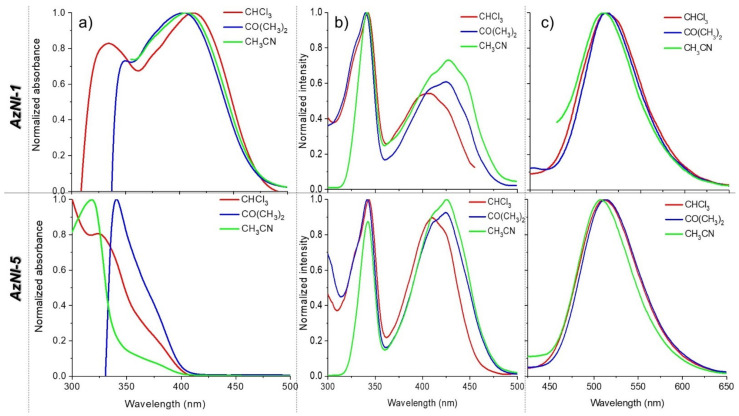
The (**a**) electronic absorption, (**b**) excitation, and (**c**) emission (λ_ex_ = 340 nm) spectra of AzNI–1 and AzNI-5 in various solvents.

The optical properties of the new azomethino-1,8-naphthalimides were tested in various solvents, such as: CHCl_3_ (chloroform*,* **ε** *=* 4.89), CO(CH_3_)_2_ (acetone*,* **ε** *=* 20.56) and CH_3_CN (acetonitrile*,* **ε** *=* 35.94) solutions in 10^−5^ mol/dm^3^ concentration. In addition, studies were performed also for thin films and blends with PVK:PBD matrix (50 wt.%:50 wt.%) with 2 or 15 wt.% AzNI content and obtained on the glass substrate (Figure S12). Photoluminescence (PL) properties were described by the quantum yields (Φ) and fluorescence lifetimes (τ). The obtained spectroscopic data are listed in Table 3 and Table 4, whereas in Figure 6 as well as in Figure S6–S10 the appropriate PL spectra are presented.

Analyzing the collected data, it can be noticed that the substituent in the naphthalene unit influences the electronic absorption properties. For the compounds with condensed aromatic ring substituents (AzNI-1 with 9-anthracene and AzNI-3 with 1-pyrene), two absorption bands are visible in the range from 300 nm to 500 nm (4.13–2.48 eV), while for the other compounds only one absorption maximum (λ_max_) is seen, between 300 nm and 350 nm (4.13–3.54 eV, Figure 6 and Supplementary Figure S6) in various solvents.

The absorption band with the maximum (λ_max_) between 318 nm–349 nm corresponds to π–π* transitions in the imide unit [38], while the second band from 374 nm to 415 nm seen in UV-Vis spectra of AzNI-3 and AzNI-1 comes from fused phenyl rings, such as pyrene (AzNI-3) and anthracene (AzNI-1) (Figure 6 and Supplementary Figure S6). The absorption band in the lower energies (3.32–2.99 eV) can be attributed to the charge transfer (CT) between the substitutes and naphthalimide (intra molecular charge transfer/locally excited (ICT/LE) nature) [22,36,38]. A better degree of conjugation for azomethino-1,8-naphthalimide with anthracene substituent was noticeable, confirmed by NMR and FTIR spectra and the position of λ_max_ in the lower energies [38]. Similar behavior was observed for bis-(imino-1,8-naphthalimides) with triphenylamine described in our recent publication [36].

In thin films, the position of λ_max_ was registered between 344 nm (for AzNI-4 with (2-cyanoethyl)methylamine) and 424 nm (for AzNI-1 with anthracene). The compounds AzNI-2 (naphthalene substituent) and AzNI-5 (biphenyl substituent) did not form good quality film on the glass substrates [41]. The bathochromic shift was observed in thin films for compounds with 1-pyrene (AzNI-3, ∆λ_max_ = 15 nm), 9-anthracene (AzNI-1, ∆λ_max_ = 13 nm), and 4-(2-phenyleth-1-ynyl)benzene (AzNI-6, ∆λ_max_ = 21 nm) compared with the chloroform solution. A similar position of λ_max_ in chloroform solution and a thin film was observed in the case of AzNI-4 (with (2-cyanoethyl)methylamino-4-benzene) compound. A bathochromic shift is also observed in the maximum emission band (λ_em_) of the layer relative to the chloroform solution for AzNI-3 (Table 4). This behavior can be explained by the presence of the J-aggregates in the thin film, as confirmed by the absorption and emission spectra [42]. In the absorption spectra of the PVK:PBD blends, only the extensions of the absorption band from the matrix were observed (at λ_max_= 310 nm and λ_max_ = 344 nm) [43]. The PVK:PBD blends containing 15 wt.% of the AzNI-1 and AzNI-3 showed absorption bands at lower energies, corresponding to the investigated compounds (Table 3).

The presented azomethino-1,8-naphthalimides emitted light in a blue spectral region in the solutions, and only for the compound AzNI-4 was a green emission in chloroform seen. The slight shift of the maximum emission band toward lower wavelength values was observed in AzNI-4 by increasing the solvent polarity (λ_em_= 523 nm in CHCl_3_ and λ_em_= 511 nm in CH_3_CN) (Table 4). The calculated Stokes shift varies from 4946 cm^−1^ to 11,507 cm^−1^ depending on the compound structure and solvents (Table 4). The compounds generally showed a low emission intensity (Ф at about 0.11–0.51% in acetonitrile or 0.01–4.42% in chloroform). As mentioned in Section 3.3, the emission can be deactivated because of internal energy conversion (ISC). However, in the deactivation of the singlet excited state the PET mechanism can be responsible (the photoinduced electron transfer), owing to the fact that HOMO and LUMO are localized mainly on the substituent and 1,8-napthalimide parts of the molecules (Table S1 and Figure S5) and the PET may occur from HOMO to LUMO of both aromatic fragments with participation of the azomethine linker.

In previous works [37,38,44], it was found that an increase in photoluminescence through the protonation of the imine bond related to the inhibition of the PET process can take place. The non-radiative decay rates outperformed radiation processes (Table 4). The same trend was observed in our previous investigations [44]. In most cases, a lower quantum yield value was seen in a polar solvent, only for AzNI-6 differences were insignificant.

The emission in the solid-state in thin film was visible only for AzNI-3 (with pyrene), AzNI-4 (with (2-cyanoethyl)methylamine) and AzNI-6 (with 4-(2-phenyleth-1-ynyl)benzene) in the green (AzNI-3 and AzNI-4) and violet (AzNI-6) spectral region. The compounds AzNI-3 and AzNI-6 had the highest quantum yields in a solid-state than in the chloroform solution; however, AzNI-4 was characterized by the quenching of the emission in the aggregated state (Ф = 2.74% in film and Ф = 4.42% in chloroform). The emission investigations were also performed for blends (PVK:PBD:AzNI), where the host (PVK: PBD) and the guest (AzNI) structure were created [45]. Transfer of the energy in the host-guest structure may occur, consisting of the transfer of the energy from the host to the guest in the ground state [43,46]. The transfer process may take place according to the resonance (Förster transfer) or exchange (Dexter) mechanism as a non-radiative energy transfer [43]. The Förster energy transfer is a result of dipole-dipole interactions. It can occur between the guest and the host at greater distances than the exchange mechanism. In the case of the Dexter energy transfer, the distance between the guest and the host must be minimal, and their electron clouds must overlap. The triplet-triplet energy transfer is allowed in the exchange mechanism, while in the singlet-singlet, the resonance mechanism [47]. The Förster energy transfer may occur when the host’s photoluminescent band (PL) coincides with the guest absorption spectrum, the distance condition is maintained, and the host emission lifetime is sufficiently long. The energy transfer occurs when the emission intensity of the guest in the presence of the host increases and the host decreases [48]. In a solid-state and in solutions, the overlapping of the PL PVK:PBD matrix with absorption spectrum of the AzNI was visible for compounds with anthracene (AzNI-1) and pyrene (AzNI-3) (Figure S10). In the emission spectra of the PVK:PBD:AzNI blends two bands were seen, one band localized mainly in the PVK:PBD PL spectrum range and the second one localized at higher energies (λ_em_ ≈ 470–513 nm) (Figure S10). In the PVK:PBD:AzNI-2 and PVK:PBD:AzNI-5 systems the energy transfer between AzNI and PVK:PBD matrix is rather ineffective, there was no significant increase in the guest emission in the presence of the host. The effective energy transfer can be seen in the case of blends with compounds containing anthracene (AzNI-1), pyrene (AzNI-3), and 2-cyanoethyl)methylamino-4-benzene (AzNI-4) with 2 wt.% content in the matrix AzNI-3 and 15 wt.% content in the matrix AzNI-1 and AzNI-4, for which an increase in the guest emission and a decrease in the matrix emission were observed.

The symmetrical analogues [36] showed a bathochromic shift of the absorption and emission bands, with significant differences in the compound with an ethynyl bond (AzNI-6 in this work). For the asymmetrical structure, a hypsochromic shift of the maximum emission band in the form of a thin layer was obtained by as much as 98 nm in relation to the symmetrical analog and bathochromic shift by 38 nm in a chloroform solution. For the 5,5′-(biphenyl-4,4′-diimine)-bis (2-(2-hylhexyl)-1*H*-benzo [de] isoquinoline-1,3 (2*H*)-dione) absorption and emission spectra were registered in the solid-state form, which proves better properties of the layer-forming ability thanks to the presence of the second naphthalimide group.

#### 3.4.2. Electroluminescence

**Figure 7:**
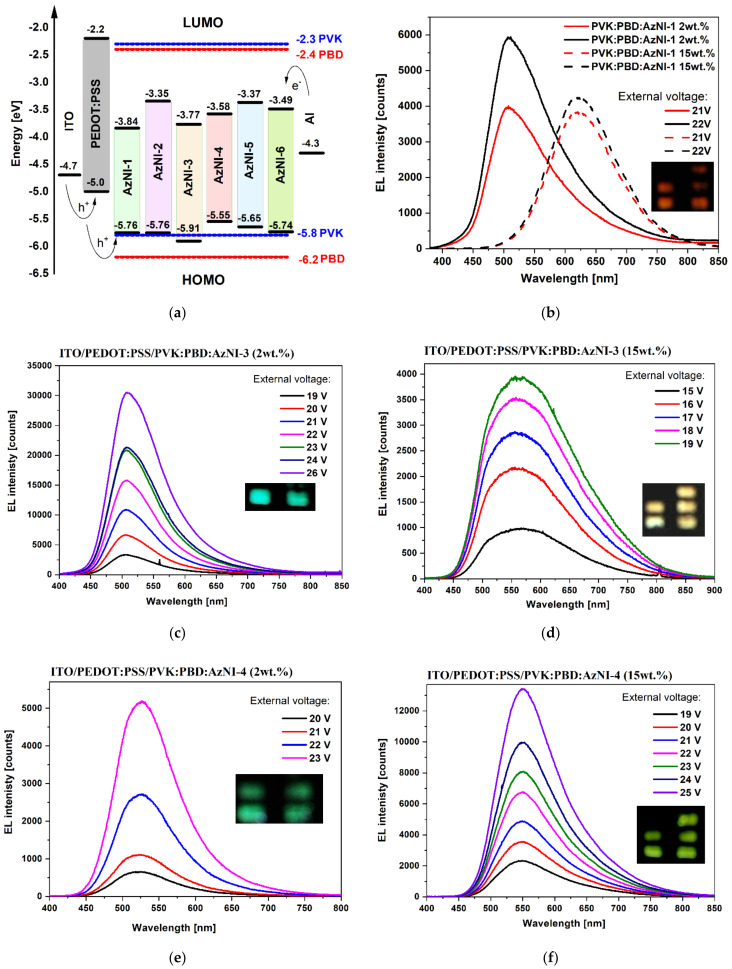

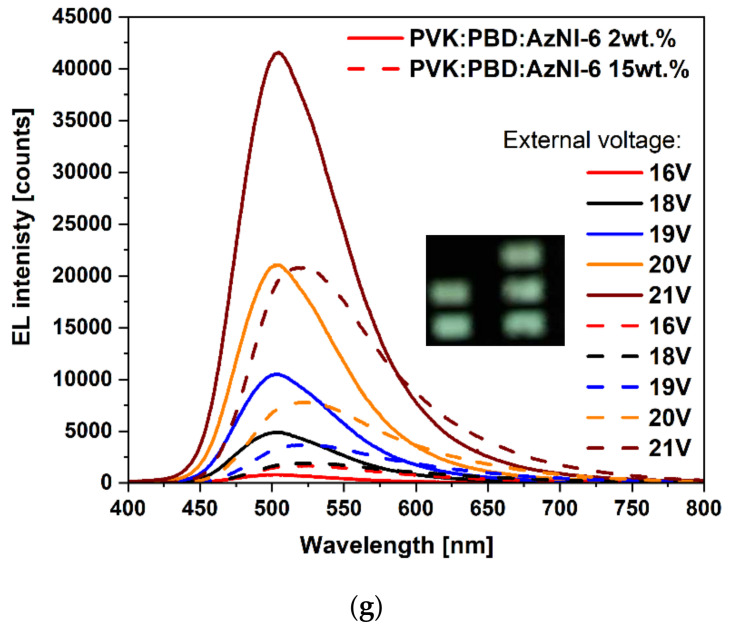
(**a**) HOMO and LUMO energy levels with work function of ITO and Al, (**b**–**g**) electroluminescence spectra’s of the tested diodes (insert: photos of the working diodes).

Initial studies to verify the ability of selected compounds to exhibit electroluminescence (EL) were carried out. For this purpose, diodes, in which the obtained compounds acted as active layers, or their component (guest-host structure) were constructed. The prototype devices with the architecture ITO/PEDOT:PSS/compound/Al and ITO/PEDOT:PSS/PVK:PBD:compound/Al were constructed. Glass coated with a layer of indium tin oxide (ITO), and aluminum (Al) acted as electrodes, anode, and cathode, respectively. PEDOT:PSS (poly(3,4-ethylenedioxythiophene) polystyrene sulfonate) was used as the ITO smoothing layer as the unevenness of the ITO layer during vacuum evaporation can cause breakdown when higher voltage is applied. PEDOT:PSS was also used as a layer facilitating hole injection, and PVK and PBD acted as a hole and electron conductive material, respectively. The content of the compounds AzNI in the PVK:PBD (50 wt.%:50 wt.%) matrix was 2 and 15 wt %. Electroluminescence spectra were obtained for various values of the applied voltage. The results are collected in Table 5 and EL spectra are presented in Figure 7.

Diodes with the active layer based on a neat azomethinoimides AzNI did not emit light under the external voltage. Diodes with the guest-host structure (PVK:PBD:compound) emitted light from blue to red spectral region, except for the device with AzNI-2 (1-naphthalene substituent) and AzNI-5 (4-phenyl-benzyl substituent) (Table 5), where the EL spectra were not registered. The blue electroluminescence in the case of diodes with 2 wt.% content of AzNI-1 (anthracene substituent, λ_EL_ = 513 nm), AzNI-3 (1-pyrene substituent, λ_EL_ = 506 nm), and AzNI-6 (4-(2-phenyleth-1-ynyl)benzene substituent, λ_EL_ = 506 nm) in the PVK:PBD matrix were observed. The devices with 2 and 15 wt.% content of AzNI-4 ((2-cyanoethyl)methylamine substituent, λ_EL_ = 526 nm, and λ_EL_ = 551 nm) and 15 wt.% content of AzNI-6 (4-(2-phenyleth-1-ynyl)benzene substituent, λ_EL_ = 519 nm) emitted light in green spectral region (Figure 7e–g). The yellow and red electroluminescence was registered for diodes with 15 wt.% content of AzNI-1 (λ_EL_ = 620 nm) and AzNI-3 (λ_EL_ = 560 nm). The highest intensity of EL was obtained for a device with 2 wt.%.content of azomethinoimide with 4-(2-phenyleth-1-ynyl)benzene substituent (AzNI-6) in the matrix under 21 V. It was observed that with an increase in the compound’s content in the active layer the bathochromic shift of the λ_EL_ take places (blue → red for AzNI-1, blue → yellow for AzNI-3 and blue → green for AzNI-6) with the reduction of the EL intensity. No changes in the λ_EL_ position’s dependence on the compounds content in the PVK:PBD matrix were found for the diode with AzNI-4 with (2-cyanoethyl)methylamine, but the increase of EL intensity was seen. Above 26 V, the light-emitting diodes degraded.

The lack of EL of devices containing a neat AzNI compounds in the active layer may result from problems in the carrier transport, the low charge carrier mobility, or the poor ability to create stable coatings. The use of the two-component PVK: PBD matrix allowed for the registration of the EL spectra. Moreover, the LUMO orbital of the AzNI compounds was below the LUMO of the PVK and PBD, while the HOMO oscillated around the HOMO orbital of the matrix (Figure 7a). Such a location of the orbitals may indicate efficient energy transfer from the matrix and the dominance of the Förster mechanism. Based on the absorption and emission investigations, the Förster mechanism is unlikely to exist in PVK:PBD:AzNI-6 configuration. However, in the recombination process in OLED devices, the energy transfer processes and the mechanism of trapping charges may coexist and the presence of these mechanisms may be seen in our OLED structures [49]. To better understand the processes that are taking place in the presented devices, it is necessary to conduct additional research.

The effect of the number of aromatic rings on the electroluminescence spectrum was observed. EL was not observed for a diode based on the compound with 1-naphthalene (AzNI-2), as was previously mentioned. For the compound with anthracene, the EL spectrum was obtained with a maximum in the blue spectral range (2 wt.% content in the matrix), and for the compound with phenanthrene red EL was observed for the 2 wt.% content in the PVK:PBD matrix [38]. The device based on a compound with pyrene showed blue EL. Increasing the number of the aromatic rings did not shift the EL spectrum toward longer wavelengths. The symmetrical analogues [36] to AzNI-5 and AzNI-6 showed EL in green (2 wt.% content in PVK:PBD, λ_EL_ = 525 and 259 nm) and red (ITO/PEDOT:PSS/symmetrical analog to AzNI-5/Al,λ_EL_ = 675 nm) spectral regions. Devices with AzNI-5 (4-phenyl-benzyl substituent) did not emit light. Comparing the 2 wt.% content of the AzNI-6 (4-(2-phenyleth-1-ynyl)benzyl substituent) in the matrix and its symmetrical analog, a bathochromic shift of the EL spectrum of the symmetrical counterpart was observed, while higher EL intensity was obtained for the asymmetrical structure (AzNI-6) at a lower operating voltage.

In our previous publications [34,36,38,44,45], the electroluminescence study of the imino-imides was also performed. Based on these investigations, the perspective compounds as materials for OLED applications are 1,8-napthalimides with triphenylamine substituent [38,45]. As mentioned in the Introduction, the presence of triphenylamine in the 4-C position in the naphthalene ring and the doping of dimethyl-4,7-diphenyl-1,10-phenanthroline allowed obtaining a stable yellowish-green light, and in our case, the doping of PVK:PBD also allowed for the recording of EL spectra with the possibility of obtaining different colors [32,38,45]. Most publications on naphthalimides in organic electronics concern compound substituted at the 4-C position in the naphthalene ring to obtain the blue, green, orange, red, and even white emitters in OLEDs [16]. Depending on the substituents on the imide ring and naphthalene ring, diodes of various colors can be obtained. An essential element is also the structure of the organic diode, which is properly designed to obtain clear and time-stable colors. Scientists are also researching compounds showing thermally activated delayed fluorescence (TADF) as more efficient materials, whereas in the diode structure also the doping materials were used to obtain more stable devices, thus receiving orange and red emitters [5,50].

## 4. Conclusions

Six new compounds with the imine bond and 1,8-naphthalimide main molecule fragment were synthesized and characterized considering their important properties for optoelectronics. Based on the obtained results from performed investigations, it can be concluded that:

- crystalline compounds with T_m_ in the range of 132–182 °C with the possibility of their amorphization were obtained,
- azomethinoimides were electrochemically active with the low energy band gap (below 2.41 eV),
- the molecules showed a low PL quantum yield, due to probably the photoinduced electron transfer process and the prevailing non-radiation processes,
- an energy transfer from the PVK:PBD matrix to AzNI was observed in the blends, unfortunately slightly affecting the quantum yield values. However, the use of the matrix allowed to induction the emission of light under external voltage and the maximum of electroluminescence band from the blue to red spectral region dependent on the compounds content was seen.

Our previous works demonstrated the ability to cancer cellular imaging of the azomethino-1,8-naphthalimides substituted at the 3-C position. Therefore, the biological investigations will be performed for this series of compounds in the next research step.

## Figures and Tables

**Table 1:** TGA and DSC data of the investigated *N*-hexyl-1,8-naphthalimides derivatives.

Molecule	TGA			DSC			
				I Heating Scan	II Heating Scan		
	T_5_^a^ (°C)	T_max_^b^ (°C)	Residue	T_m_^c^	T_g_^e^	T_c_^d^	T_m_^c^
			at 600 °C (%)	(°C)	(°C)	(°C)	(°C)
**AzNI-1**	322	411	21	175	51	135	172
**AzNI-2**	315	380	4	132	26	120	131
**AzNI-3**	372	460	10	182	58	nd	nd
**AzNI-4**	283	301,407	8	158	24	127	160
**AzNI-5**	318	387	2	145	23	nd	nd
**AzNI-6**	359	527	50	151	29	90	151

^a^ T_5_—temperature based on 5% weight loss. ^b^ Temperature of the maximum decomposition rate from DTG curves. ^c^ Melting temperature. ^d^ Cold crystallization temperature. ^e^ Glass transition temperature. nd—not detected. TGA were measured in the range of 25–600 °C, and DSC were measured to 250 °C.

**Table 2:** The redox properties of the azomethino-1,8-naphthalimides.

Molecule	Method	E_red_^1^	E_red_^1(onset)^	E_ox_^1^	E_ox_^1(onset)^	E_LUMO_	LUMO ^a^	E_HOMO_	HOMO ^a^	E_g_
		[V]	[V]	[V]	[V]	[eV]	[eV]	[eV]	[eV]	[eV]
**AzNI–1**	CV	−1.69 ^a^	−1.26	0.82 ^a^	0.66	−3.84	−2.50	−5.76	−5.48	1.92
	DPV	−1.58	−1.34	0.73	0.62	−3.76		−5.72		1.96
**AzNI–2**	CV	−1.88 ^b^	−1.75	0.87 ^a^	0.66	−3.35	−2.46	−5.76	−5.89	2.41
	DPV	−1.92	−1.65	0.78	0.56	−3.45		−5.66		2.21
**AzNI–3**	CV	−1.92 ^a^	−1.33	0.95 ^a^	0.81	−3.77	−2.48	−5.91	−5.54	2.14
	DPV	−1.71	−1.39	0.72	0.62	−3.71		−5.72		2.01
**AzNI–4**	CV	−1.98 ^a^	−1.52	0.69 ^a^	0.45	−3.58	−2.40	−5.55	−5.46	1.97
	DPV	−1.73	−1.41	0.49	0.31	−3.69		−5.41		1.72
**AzNI–5**	CV	−1.86 ^b^	−1.73	0.75 ^a^	0.55	−3.37	−2.45	−5.65	−6.01	2.28
	DPV	−1.85	−1.67	0.73	0.59	−3.43		−5.69		2.26
**AzNI–6**	CV	−1.85 ^a^	−1.61	0.74 ^a^	0.64	−3.49	−2.48	−5.74	−5.86	2.25
	DPV	−1.82	−1.60	0.64	0.49	−3.50		−5.59		2.09

E_HOMO_ = (−5.1−E_ox_^1(onset)^)·|e|, E_LUMO_ = (−5.1 − E_red_^1(onset)^)·|e|, E_g_ = E_ox_^1(onset)^−E_red_^1(onset)^. Solvent: CH_2_Cl_2_ and 0.1 mol/dm^3^ Bu_4_NPF_6_ and platinum wire as a working electrode. ^a^ Irreversible process. ^b^ *Quasi*-reversible process. E_ox_^1^—the first oxidation process, E_red_^1^—the first reduction process, E_red_^1(onset)^—the onset potential of the first reduction process, E_ox_^1(onset)^—the onset potential of the first oxidation process. E_HOMO_ and E_LUMO_ as IP and EA. v = 0.1 V/s for CV and v = 0.05 V/s for DPV. ^a^LUMO and HOMO calculated by DFT.

**Table 3:** UV-Vis absorption data of synthesized compounds.

Molecule	CHCl_3_	CO(CH_3_)_2_	CH_3_CN	Film	Blend PVK:PBD	Blend PVK:PBD
	λ_max_ (ε × 10^4^) ^a^ (nm)				2 wt.%^b^	15 wt.%^b^
**AzNI-1**	-	-	-	-	310 ^sh^	310 ^sh^
	331(2.1)	347 ^sh^			344 ^sh^	344 ^sh^
	411(2.5)	403(1.7)	406(1.9)	424	-	425
	-		-	-	310 ^sh^	310 ^sh^
**AzNI–2**	338(7.5)	337(3.6)	334(3.6)		344 ^sh^	344 ^sh^
	378 ^sh^	375 ^sh^	375 ^sh^		-	-
**AzNI–3**	-	-	-	-	310 ^sh^	310 ^sh^
	346 ^sh^	337(4.6)	-	-	344 ^sh^	344 ^sh^
	381(3.2)	-	378(4.9)	-	-	383
	405(3.0)	400 ^sh^	400 ^sh^	420	-	405
**AzNI–4**	349(4.3)	348(5.7)	348 ^sh^	344	310 ^sh^	310 ^sh^
					344 ^sh^	344 ^sh^
**AzNI–5**	323(5.5)	340(3.9)	318(24.4)	-	310 ^sh^	310 ^sh^
					344 ^sh^	344 ^sh^
**AzNI–6**	335(6.3)	340(6.1)	319(7.1)	356	310 ^sh^	310 ^sh^
					344 ^sh^	344 ^sh^

Solutions: CHCl_3_**ε** = 4.89, CO(CH_3_)_2_**ε** = 20.56, CH_3_CN **ε** = 35.94. Concentration of the solutions 10^−5^ mol/dm^3^. ^a^ ε—absorption coefficient, [dm^3^·mol^−1^·cm^−1^]. ^b^ 2 wt.% or 15 wt.% concentration of the compound in the matrix PVK:PBD. ^sh^—shoulder.

**Table 4:** PL data of the synthesized azomethino-1,8-naphthalimides.

Molecule	Medium	λ_max_	λ_em_	Stokes Shift ^c^	Φ	τ_eff_	X^2^	k_r_·10^6 d^	k_nr_·10^6 d^
		(nm)	(nm)	(cm^−1^)	(%)	(ns)		(s^−1^)	(s^−1^)
**AzNI-1**	**CHCl_3_**	331	511	10,642	2.00	14.01	0.986	1.43	69.95
		411	511	4761	0.42	-	-	-	-
	**CO(CH_3_)_2_**	403	511	5244	-	-	-	-	-
	**CH_3_CN**	406	508	4946	0.27	-	-	-	-
	**Blend PVK:PBD ^a^**	310	380;470	5942	3.42;1.56	-	-	-	-
	**Blend PVK:PBD ^b^**	310	380;494	5942	3.26;2.06	-	-	-	-
**AzNI-2**	**CHCl_3_**	338	511	10,016	2.45	9.81	1.029	2.50	99.44
	**CO(CH_3_)_2_**	337	506	9911	-	-	-	-	-
	**CH_3_CN**	334	510	10,332	0.22	-	-	-	-
	**Blend PVK:PBD ^a^**	310	379;493	5873	4.02;1.41	-	-	-	-
	**Blend PVK:PBD ^b^**	310	379;493 ^sh^	55,873	3.58	-	-	-	-
**AzNI-3**	**CHCl_3_**	381	499	6207	1.40	9.48	1.159	1.48	104.01
		405	509	5045	0.20	-	-	-	-
	**CO(CH_3_)_2_**	337	513	10,180	-	-	-	-	-
	**CH_3_CN**	378	508	6770	0.11	-	-	-	-
	**Film**	420	547	5528	3.52	-	-	-	-
	**Blend PVK:PBD ^a^**	310	392;478	6748	2.50;2.10	-	-	-	-
	**Blend PVK:PBD ^b^**	310	377;494	5733	4.50;1.95	-	-	-	-
**AzNI-4**	**CHCl_3_**	349	523	9533	4.42	8.25	1.028	5.36	115.85
	**CO(CH_3_)_2_**	348	511	9166	-	-	-	-	-
	**CH_3_CN**	348	511	9166	0.69	-	-	-	-
	**Film**	344	566	11,402	2.74	-	-	-	-
	**Blend PVK:PBD ^a^**	310	384;495	6216	2.40;1.70	-	-	-	-
	**Blend PVK:PBD ^b^**	310	380;513	5942	2.60;4.60	-	-	-	-
**AzNI-5**	**CHCl_3_**	323	511	11,390	2.65	10.89	1.069	2.43	89.39
	**CO(CH_3_)_2_**	340	512	9881	-	-	-	-	-
	**CH_3_CN**	318	507	11,723	0.32	-	-	-	-
	**Blend PVK:PBD ^a^**	310	378;492	5803	3.70;1.87	-	-	-	-
	**Blend PVK:PBD ^b^**	310	378;494 ^sh^	5803	4.82	-	-	-	-
**AzNI-6**	**CHCl_3_**	335	508	10,166	0.46	15.68	1.069	0.29	63.48
	**CO(CH_3_)_2_**	340	513	9919	-	-	-	-	-
	**CH_3_CN**	319	504	11,507	0.51	-	-	-	-
	**Film**	356	411	3759	2.13	-	-	-	-
	**Blend PVK:PBD ^a^**	310	395;472	6942	2.80;2.50	-	-	-	-
	**Blend PVK:PBD ^b^**	310	383;496	6148	3.50;2.10	-	-	-	-

c_solution_ = 10^−5^ mol/dm^3^, **^a^** 2 wt.% of the compound in the PVK:PBD (50:50 wt.%), ^b^ 15 wt.% of the compound in the PVK:PBD (50 wt.%:50 wt.%), ^c^ Stokes shifts, Δν = (1/λ_abs_ − 1/λ_em_)⋅10^7^ [cm^−1^], The dominant band have been underlined. ^d^ k_r_—radiative decay rates, k_r_ = Ф/τ_eff_, k_nr_—non-radiative decay rates, k_nr_ =(1 − Ф)/τ_eff_. ^sh^—shoulder.

**Table 5:** Electroluminescence intensity of the OLED devices with the maximum of the electroluminescence band (λ_EL_) under external voltage (U_EL_) applied.

	Devices Parameters		
The Active Layer	λ_EL_^a^	EL_Max_^b^	U_ELMax_^c^
	(nm)	(counts)	(V)
PVK:PBD:**AzNI-1** 2 wt.%	513	7580	23
PVK:PBD:**AzNI-1** 15 wt.%	620	4233	22
PVK:PBD:**AzNI-3** 2 wt.%	506	30,430	26
PVK:PBD:**AzNI-3** 15 wt.%	560	3931	19
PVK:PBD:**AzNI-4** 2 wt.%	526	5187	23
PVK:PBD:**AzNI-4** 15 wt.%	551	13,419	25
PVK:PBD:**AzNI-6** 2 wt.%	506	41,681	21
PVK:PBD:**AzNI-6** 15 wt.%	519	20,781	21

^a^ λ_EL_—maximum of the electroluminescence band, ^b^ EL_Max_—maximum intensity at λ_EL_, ^c^ U_ELMax_—external voltage for the maximum electroluminescence intensity.

## Data Availability

Not applicable.

## Supplementary Materials

The following supporting information can be downloaded at: https://www.mdpi.com/article/10.3390/ma15197043/s1, Figure S1: ^1^H NMR(DMSO-d_6_, 400 MHz) and ^13^C NMR spectra (DMSO-d_6_ or CDCl_3_, 101 MHz); Figure S2: Photographs of the solid state of AzNIs under daylight and under UV irradiation; Figure S3: (a) TGA thermograms and DSC thermograms of (b) AzNI-5 and (c) AzNI-6; Figure S4: Optimized geometries of the compounds AzNI; Figure S5: Density-of-states diagrams; Figure S6: The absorption spectra of the *N*-hexyl-1,8-naphthalimides derivatives (AzNI-1–6) in the various solvents with the emission spectra of the PVK:PBD matrix; Figure S7: The excitation and emission spectra of the *N*-hexyl-1,8-naphthalimides derivatives (AzNI-2, 3, 4, 6) in the various solvents (λ_ex_ = 340 nm); Figure S8: The absorption and excitation spectra of the *N*-hexyl-1,8-naphthalimides derivatives (AzNI-1–6) in acetone; Figure S9: The 3D *fluorescence* spectra of the analyzed compounds in the excitation range from 300 to 500 nm and the collected emissions in the range from 400 to 650 nm in the chloroform (CHCl_3_), acetone (CO(CH_3_)_2_) and acetonitryle (CH_3_CN). Measurements were performed for equal concentration of each compound (c = 1·10^−5^ mol/dm^3^) and under the same measurement conditions; Figure S10: Normalize absorption spectra of a thin films and emission spectra in solutions and blends PVK:PBD:AzNI (and selected a thin films) with emission of the matrix; Figure S11: The dependence of the Stokes Shift on solvent orientation polarizability *Δf*. Stokes shifts calculated according to the equation Δν = (1/λ_abs_ − 1/λ_em_)⋅10^7^ [cm^−1^]. *Δf* stands for the orientation polarizability defined as: Δ*f* = [(ε − 1)/(2ε − 1)] − [(n^2^ − 1)/(2n^2^ − 1)], where *ε* and *n* are the dielectric constants and the refractive index of the solvent, respectively; Figure S12: The demonstrative drawing of obtaining thin layers on the glass substrate; Figure S13: CV of the samples 1-6 (AzNI-1–AzNI-6) (except AzNI-5, sample no5) in 0.1 M tetrabutylammonium hexafluorophosphate (Bu_4_NPF_6_) in dry acetonitrile (ACN) after Ar bubbling, at room temperature; Table S1: Ground and S_1_ state composition of selected molecular orbitals; Table S2: Calculated, in the chloroform solution, dipole moments and mean plane angles for the imine molecules; Table S3: The calculated electronic transitions corresponding to excitation wavelength in CHCl_3_ solution; Table S4: Energy differences between excited states; Table S5: Life-time measurements with a time-correlated single photon counting (TCSPC) method; Table S6: The electrochemical band gap and the optical band gap; Table S7: The electrochemical data investigated in acetonitrile. References [51,52,53,54,55,56,57] are cited in the Supplementary Materials.

## References

[1] Lee C.W., Kim O.Y., Lee J.Y. (2014). Organic materials for organic electronic devices. J. Ind. Eng. Chem..

[2] Das S., Dhara S. (2021). Chemical Solution Synthesis for Materials Design and Thin Film Device Applications.

[3] Yuan Q., Wang T., Yu P., Zhang H., Zhang H., Ji W. (2021). A review on the electroluminescence properties of quantum-dot light-emitting diodes. Org. Electron..

[4] Hu Y.-X., Xia X., He W.-Z., Tang Z.-J., Lv Y.-L., Li X., Zhang D.-Y. (2019). Recent developments in benzothiazole-based iridium(Ⅲ) complexes for application in OLEDs as electrophosphorescent emitters. Org. Electron..

[5] Wang Z., Wang C., Zhang H., Liu Z., Zhao B., Li W. (2019). The application of charge transfer host based exciplex and thermally activated delayed fluorescence materials in organic light-emitting diodes. Org. Electron..

[6] Matsui H., Takeda Y., Tokito S. (2019). Flexible and printed organic transistors: From materials to integrated circuits. Org. Electron..

[7] Wang J., Yao H., Xu Y., Ma L., Hou J. (2021). Recent progress in reducing voltage loss in organic photovoltaic cells. Mater. Chem. Front..

[8] Zheng H., Li D., Ran C., Zhang Q., Song L., Chen Y., Muller-Buschbaum P., Huang W. (2021). Emerging Organic/Hybrid Photovoltaic Cells for Indoor Applications: Recent Advances and Perspectives. RRL Sol..

[9] Xiang L., Gao F., Cao Y., Li D., Liu Q., Liu H., Li S. (2022). Progress on the stability and encapsulation techniques of perovskite solar cells. Org. Electron..

[10] Ziarani G.M., Moradi R., Lashgari N., Kruger H.G. (2018). Chapter 11-Imide Dyes. Metal-Free Synthetic Organic Dyes.

[11] Gan J.-A., Song Q.L., Hou X.Y., Chen K., Tian H. (2004). 1,8-Naphthalimides for non-doping OLEDs: The tunable emission color from blue, green to red. J. Photochem. Photobiol. A Chem..

[12] Ulla H., Kiran M.R., Garudachari B., Satyanarayan M.N., Umesh G., Isloor A.M. (2014). Blue emitting halogen–phenoxy substituted 1,8-naphthalimides for potential organic light emitting diode applications. Opt. Mater..

[13] Yordanova S., Grobchev I., Stoyanov S., Milusheva V., Petkov I. (2014). Synthesis and functional characteristics of two new yellow-green fluorescent PAMAM dendrimers periphery modified with 1,8-naphthalimides. Inorg. Chim. Acta.

[14] Bojinov V.B., Simeonov D.B. (2010). Synthesis of highly photostable blue-emitting 1,8-naphthalimides and their acrylonitrile copolymers. Polym. Degrad. Stab..

[15] Prezhdo O.V., Uspenskii B.V., Prezhdo V.V., Boszczyk W., Distanov V.B. (2007). Synthesis and spectral-luminescent characteristics of *N*-substituted 1,8-naphthalimides. Dye. Pigment..

[16] Kagatikar S., Sunil D. (2022). A systematic review on 1,8-naphthalimide derivatives as emissive materials in organic light-emitting diodes. J.Mater. Sci..

[17] Sonalin S., Sakthivel K., Nagarajan S. (2018). Functionalization of 1, 8-Naphthalimides—An approach towards air-stable n- type organic semiconductors. Mater. Today Proc..

[18] Poddar M., Sivakumar G., Misra R. (2019). Donor–acceptor substituted 1,8-naphthalimides: Design, synthesis, and structure–property relationship. J. Mater. Chem. C.

[19] Saini A., Thomas K.R.J., Sachdev A., Gopinath P. (2017). Photophysics, Electrochemistry, Morphology, and Bioimaging Applications of New 1,8-Naphthalimide Derivatives Containing Different Chromophores. Chem. Asian J..

[20] Izawa H., Yasufuku F., Nokami T., Ifuku S., Saimoto H., Matsui T., Morihashi K., Sumita M. (2021). Unique Photophysical Properties of 1,8-Naphthalimide Derivatives: Generation of Semi-stable Radical Anion Species by Photo-Induced Electron Transfer from a Carboxy Group. ACS Omega.

[21] Masimukku N., Gudeika D., Volyniuk D.Y., Bezvikonnyi O., Simokaitiene J., Matulis V., Lyakhov D., Azovskyi V., Gražulevičus J.V. (2022). Bipolar 1,8-naphthalimides showing high electron mobility and red AIE-active TADF for OLED applications. Phys. Chem. Chem. Phys..

[22] Rout Y., Misra R. (2021). Design and synthesis of 1,8-naphthalimide functionalized benzothiadiazoles. New J. Chem..

[23] Kelly L.A., Roll M., Joseph J., Seenisamy J., Barrett J., Kauser K., Warner K.S. (2021). Solvent-Dependent Photophysics and Reactivity of Monomeric and Dimeric 4-Amino-1,8-Naphthalimides. J. Phys. Chem. A.

[24] Zhao Q., Yang Q. (2021). Tetraphenylethenyl-Modified 1,8-Naphthalimide Dye with Efficient Aggregation-Enhanced Emisssion, Solvatochromism and Intramolecular Charge Transfer Characteristics. J. Phys. Conf. Ser..

[25] Adair L.D., Trinh N., Verite P.M., Jacquwmin D., Jolliffe K.A., New E.J. (2020). Synthesis of Nitro-Aryl Functionalised 4-Amino-1,8-Naphthalimides and Their Evaluation as Fluorescent Hypoxia Sensors. Chem. A Eur. J..

[26] Yin Y., Chen Z., Fan C., Liu G., Pu S. (2019). 1,8-Naphthalimide-Based Highly Emissive Luminophors with Various Mechanofluorochromism and Aggregation-Induced Characteristics. ACS Omega.

[27] Pablos J.L., Hernández E., Catalina F., Corrales T. (2022). Solid Fluorescence pH Sensors Based on 1,8-Naphthalimide Copolymers Synthesized by UV Curing. Chemosensors.

[28] Betancourt F., Valente A., Yan H. (2021). 1,8-Naphthalimide derivatives as probes for protein surface hydrophobicity. J. Photochem. Photobiol. A Chem..

[29] Saito G., Velluto D., Resmini M. (2018). Synthesis of 1,8-naphthalimide-based probes with fluorescent switch triggered by flufenamic acid. R. Soc. Open Sci..

[30] Dong H.-Q., Wei T.-B., Ma X.-Q., Yang Q.-Y., Zhang Y.-F., Sun Y.-J., Shi B.-B., Yao H., Zhang Y.-M., Lin Q. (2020). 1,8-Naphthalimide-based fluorescent chemosensors: Recent advances and perspectives. J. Mater. Chem. C.

[31] Triboni E.R., Fernandes M.R., Garcia J.R., Carreira M.C., Berlinck R.G.S., Filho P.B., Roman L.S., Hümmelgen I.A., Reyes R., Cremona M. (2015). Naphthalimide-derivative with blue electroluminescence for OLED applications. J. Taibah Univ. Sci..

[32] Arunchai R., Sudyoadsuk T., Prachumrak N., Namuangruk S., Promarak V., Sukwattanasinitt M., Rashatasakhon P. (2015). Synthesis and characterization of new triphenylamino-1,8-naphthalimides for organic light-emitting diode applications. New J. Chem..

[33] Luo S., Lin J., Zhou J., Wang Y., Liu X., Huang Y., Lu Z., Hu C. (2015). Novel 1,8-naphthalimide derivatives for standard-red organic light-emitting device applications. J. Mater. Chem. C.

[34] Schab-Balcerzak E., Siwy M., Filapek M., Kula S., Malecki G., Laba K., Lapkowski M., Janeczek H., Domanski M. (2015). New core-substituted with electron-donating group 1,8-naphthalimides towards optoelectronic applications. J. Lumin..

[35] Mikroyannidis J.A., Shanghui Y., Liu Y. (2009). Electroluminesent divinylene- and trivinylene-molecules with terminal naphthalimide or phthalimide segments. Synth. Met..

[36] Kotowicz S., Korzec M., Pająk A.K., Golba S., Małecki J.G., Siwy M., Grzelak J., Maćkowski S., Schab-Balcerzak E. (2021). New Acceptor–Donor–Acceptor Systems Based on Bis-(Imino-1,8-Naphthalimide). Materials.

[37] Sęk D., Lapkowski M., Dudek H., Karoń K., Janeczek H., Jarząbek B. (2012). Optical and electrochemical properties of three-dimensional conjugated triphenylamine-azomethine molecules. Synth. Met..

[38] Kotowicz S., Korzec M., Malarz K., Krystkowska A., Mrozek-Wilczkiewicz A., Golba S., Siwy M., Maćkowski S., Schab-Balcerzak E. (2021). Luminescence and Electrochemical Activity of New Unsymmetrical 3-Imino-1,8-naphthalimide Derivatives. Materials.

[39] Bujak P., Kulszewicz-Bajer I., Zagorska M., Maurel V., Wielgus I., Pron A. (2013). Polymers for electronics and spintronics. Chem. Soc. Rev..

[40] Iwan A., Boharewicz B., Parafiniuk K., Tazbir I., Gorecki L., Sikora A., Filapek M., Schab-Balcerzak E. (2014). New air-stable aromatic polyazomethines with triphenylamine or phenylenevinylene moieties towards photovoltaic application. Synth. Met..

[41] Gaudin O.P.M., Samuel I.D.W., Amriou S., Burn P.L. (2010). Thickness dependent absorption spectra in conjugated polymers: Morphology or interference?. Appl. Phys. Lett..

[42] Más-Montoya M., Janssen R.A.J. (2017). The Effect of H- and J-Aggregation on the Photophysical and Photovoltaic Properties of Small Thiophene–Pyridine–DPP Molecules for Bulk-Heterojunction Solar Cells. Adv. Funct. Mater..

[43] Glowacki I., Szamel Z. (2010). The nature of trapping sites and recombination centres in PVK and PVK–PBD electroluminescent matrices seen by spectrally resolved thermoluminescence. J. Phys. D Appl. Phys..

[44] Korzec M., Kotowicz S., Gawecki R., Malarz K., Mrozek-Wilczkiewicz A., Siwy M., Schab-Balcerzak E., Grzelak J., Maćkowski S. (2021). 1,8-Naphthalimides 3-substituted with imine or β-ketoenamine unit evaluated as compounds for organic electronics and cell imaging. Dye. Pigment..

[45] Kotowicz S., Korzec M., Siwy M., Golba S., Malecki J.G., Janeczek H., Mackowski S., Bednarczyk K., Libera M., Schab-Balcerzak E. (2018). Novel 1,8-naphthalimides substituted at 3-C position: Synthesis and evaluation of thermal, electrochemical and luminescent properties. Dye. Pigment..

[46] Skórka Ł., Kurzep P., Wiosna-Sałyga G., Łuszczyńska B., Wielgus I., Wróbel Z., Ulański J., Kulszewicz-Bajer I. (2017). New diarylaminophenyl derivatives of carbazole: Effect of substituent position on their redox, spectroscopic and electroluminescent properties. Synth. Met..

[47] Kotwica K., Bujak P., Wamil D., Pieczonka A., Wiosna-Salyga G., Gunka P.A., Jaroch T., Nowakowski R., Luszczynska B., Witkowska E. (2015). Structural, Spectroscopic, Electrochemical, and Electroluminescent Properties of Tetraalkoxydinaphthophenazines: New Solution-Processable Nonlinear Azaacenes. J. Phys. Chem. C.

[48] Hussain S.A. (2009). An introduction to fluorescence resonance energy transfer (FRET). arXiv.

[49] Yersin H. (2008). Highly Efficient OLEDs with Phosphorescent Materials.

[50] Chen T., Lu C.-H., Huang C.-W., Zeng X., Gao J., Chen Z., Xiang Y., Zeng W., Huang Z., Gong S. (2019). Tuning the emissive characteristics of TADF emitters by fusing heterocycles with acridine as donors: Highly efficient orange to red organic light-emitting diodes with EQE over 20%. J. Mater. Chem. C.

[51] Frisch M.J., Trucks G.W., Schlegel H.B., Scuseria G.E., Robb M.A., Cheeseman J.R., Scalmani G., Barone V., Petersson G.A., Nakatsuji H. (2016). Gaussian 09.

[52] Becke A.D. (1993). Density-functional thermochemistry III. The role of exact exchange. J. Chem. Phys..

[53] Lee C., Yang W., Parr R.G. (1988). Development of the Colle-Salvetti correlation-energy formula into a functional of the electron den sity. Phys. Rev. B.

[54] Grimme S., Ehrlich S., Goerigk L. (2011). Effect of the damping function in dispersion corrected density functional theory. J. Comput. Chem..

[55] Barone V., Cossi M. (1998). Quantum Calculation of Molecular Energies and Energy Gradients in Solution by a Conductor Solvent Model. J. Phys. Chem. A.

[56] O’Boyle N.M., Tenderholt A.L., Langner K.M. (2008). A Library for Package-Independent Computational Chemistry Algorithms. J. Comput. Chem..

[57] Casida M.E., Seminario J.M. (1996). Recent Developments and Applications of Modern Density Functional Theory, Theoretical and Computational Chemistry.

