# Supplementary material for: Six New Unsymmetrical Imino-1,8-naphthalimide Derivatives Substituted at 3-C Position—Photophysical Investigations
Source: Materials (Basel). 2022 Oct 10;15(19):7043. doi: 10.3390/ma15197043 (PMC9573252; doi:10.3390/ma15197043)
Supplement: Supplementary file 1 [file materials-15-07043-s001.zip › materials-1908382-supplementary.pdf]

materials

MDPI

Article

Six New Unsymmetrical imino-1,8-naphthalimide Derivatives Substituted at 3-C Position—Photophysical Investigations

Sonia Kotowicz [1,*], Mateusz Korzec [1], Jan Grzegorz Małecki [1], Sylwia Golba [2], Mariola Siwy [3], Sebastian Maćkowski [4] and Ewa Schab-Balcerzak [1,3]

1 Institute of Chemistry, University of Silesia, 9 Szkolna Str., 40-006 Katowice, Poland
2 Institute of Materials Science, University of Silesia, 41-500 Chorzow, Poland
3 Centre of Polymer and Carbon Materials, Polish Academy of Sciences, 34 M. Curie-Sklodowska Str., 41-819 Zabrze, Poland
4 Faculty of Physics, Astronomy and Informatics, Institute of Physics, Nicolaus Copernicus University, 5 Grudziadzka Str., 87-100 Torun, Poland
* Correspondence: sonia.kotowicz@us.edu.pl

Citation: Kotowicz, S.; Korzec, M.; Małecki, J.G.; Golba, S.; Siwy, M.; Maćkowski, S.; Schab-Balcerzak, E. Six New Unsymmetrical imino-1,8-naphthalimide Derivatives Substituted at 3-C Position—Photophysical Investigations. *Materials* **2022**, *15*, 7043. https://doi.org/10.3390/ma15197043

Academic Editor: Niclas Solin

Received: 25 August 2022
Accepted: 4 October 2022
Published: 10 October 2022

Publisher's Note: MDPI stays neutral with regard to jurisdictional claims in published maps and institutional affiliations.

Copyright: © 2022 by the authors. Licensee MDPI, Basel, Switzerland. This article is an open access article distributed under the terms and conditions of the Creative Commons Attribution (CC BY) license (https://creativecommons.org/licenses/by/4.0/).

Abstract: In this research, six novel unsymmetrical imino-1,8-naphthalimides (AzNI) were synthesized. Comprehensive thermal (thermogravimetric analysis (TGA) and differential scanning calorimetry (DSC), optical (UV-Vis, photoluminescence), and electrochemical (CV, DPV) studies were carried out to characterize these new compounds. The molecules showed the onset of thermal decomposition in the temperature range 283–372 °C and molecular glass behavior. Imino-1,8-naphthalimides underwent reduction and oxidation processes with the electrochemical energy band gap (E_g) below 2.41 eV. The optical properties were evaluated in solvents with different polarities and in the solid-state as a thin films and binary blends with poly(N-vinylcarbazole): (2-tert-butylphenyl-5-biphenyl-1,3,4-oxadiazole) (PVK:PBD). Presented compounds emitted blue light in the solutions and in the green or violet spectral range in the solid-state. Their ability to emit light under external voltage was examined. The devices with guest-host structure emitted light with the maximum located in the blue to red spectral range of the electroluminescence band (EL) depending on the content of the AzNI in the PVK:PBD matrix (guest-host structure).

Keywords:1,8-naphthalimide; electrochemistry; luminescence; imines

1. Materials and characterization methods

1.1. Materials

9-Anthracenecarboxaldehyde, 1-naphthaldehyde, 1-pyrenecarboxaldehyde, 4-[(2-cyanoethyl)methylamino]benzaldehyde, 4-phenyl-benzaldehyde, 4-(2-phenyleth-1-ynyl)benzaldehyde, Pd/C, 3-nitro-1,8-naphthalic anhydride, hydrazine 98%, hexylamine, poly(N-vinylcarbazole) (PVK) and 2-(4-tert-butylphenyl)-5-(4-biphenylyl)-1,3,4-oxadiazole (PBD), Bu_4NPF_6, NaOH, dichloromethanewere purchased from Sigma Aldrich. Solvents (for HPLC) were purchased from Sigma Aldrich (Saint Lois, Missouri, USA). Poly(3,4(ethylenedioxy)thiophene):poly(styrenesulfonate) (PEDOT:PSS) and ITO glass were purchased from OSSILA (Sheffield, England).

1.2. Blends and films preparation

Films and blends on glass substrates were prepared from a homogeneous chloroform solution (10 mg/ml) of core substituted N-hexyl-1,8-naphthalimides (and with PVK:PBD (50:50 in weight %) (2 or 15 wt. % AzNIs content), respectively, by spin-coating (1000 rpm, 60s; Laurell WS 650MZ-23NPPB, North Wales, PA, USA). Films and blends

were dried for 6 h in a vacuum oven (BUCHI Glass oven B-585, Darmstadt, Germany) at 50°C.

1.3. Characterization methods

Nuclear magnetic resonance (^1H and ^{13}C NMR) spectra were recorded on a Bruker AC400 spectrometer(Berlin, Germany) in DMSO-d_6 (or $CDCl_3$) as solvent and TMS as the internal standard. The synthesis was performed using IKA C-MAG HS7 (IKA® Poland Sp. z o.o., Warszawa, Poland) and VWR VMS-C7 (WR International, LLC.) hot plates. Infrared spectra (FTIR) were recorded on a Thermo Scientific Nicolet iS5 FT-IR Spectrometer in the range of 4000–400 cm^{-1} as KBr pressed pellets (KBr before use was dried). The elementary analysis was performed using Vario EL III apparatus (Elementar, Langenselbold, Germany). Differential Scanning Calorimetry (DSC) was performed using a Du Pont 1090B apparatus (USA) with a heating/cooling rate of 20 ºC·min^{-1} under nitrogen and using aluminum sample pans in the range of 0–250 °C. The glass transition temperature was recorded in the second scan after cooling. Thermogravimetric analysis (TGA) was done with a Mettler Toledo TGA STARe system (Warszawa, Poland) with a heating rate of 10 °C·min^{-1} in a constant stream of nitrogen (20 ml·min^{-1}) and a temperature range from 25 °C to 600 °C. UV-Vis absorption spectra were performed using an Evolution 220 UV-Visible Spectrophotometer (London, United Kingdom) as the concentration of 10^{-5} mol/L and 1 cm quartz cell and Jasco V-550 Spectrophotometer (Easton, MD 21601) for films and blends. Photoluminescence spectra (PL) in solutions were performed by using Varian Carry Eclipse Spectrometer (Santa Clara, US)and using Hitachi F-2500 Spectrometer (Berkshire, United Kingdom)for blends and films PL measurements. Quantum yields (Φ_f) measurements were performed by using the integrating sphere Avantes AvaSphere-80 (Edinburgh Instruments, Austion TX, US) and absolute method. The lifetime (τ) of photoluminescence was measured with a time-correlated single photon counting (TCSPC). The time-resolved measurements were performed using the picosecond pulsed diode laser, EPL-340 nm using a 60 W microsecond Xe flash lamp (Edinburgh Instruments, Austion TX, US). Pulse period for all measurements was equal to 50 ns and PMT (Hamamatsu, R928P, Shimokanzo, Iwata City, Japan) as a detector. The fluorescence decay analysis was received an instrument response function (IRF) using ludox solution and results were presented as average values of decay after exponential fitting. Electrochemical measurements (CV) were performed with Eco ChemieAutolab PGSTAT128n potentiostat (MetrohmAutolab B.V. The Netherlands). A platinum wire (diam. 1.0 mm) served as a working electrode, while platinum coil and silver wire were used as auxiliary and reference electrode respectively. All potentials were referenced with respect to the stable internal standard, which was ferrocene couple (Fc/Fc^+). Electrochemical experiments were conducted in a one-compartment cell, in CH_2Cl_2 (ACROSS Organics, 99.9% for biochemistry grade, Janssen Pharmaceuticalaan, Belgium) and dry ACN (Acetonitrile, Sigma Aldrich for HPLC, gradient grade >99.9%, (Saint Lois, Missouri, USA)under argon purging, with 0.1M Bu_4NPF_6 (Aldrich, 99%, (Saint Lois, Missouri, USA)used as the supporting electrolyte salt. Each experiment was performed in an air-conditioned room in stable temperature (t = 20 °C ± 1°C).

1.4. Theoretical calculations

The theoretical calculations were performed with the use of the density functional theory (DFT) and were carried out using the Gaussian09 program (C.01, Gaussian, Inc., Wallingford CT, 2019) [1] on B3LYP/6-31g(d,p) level [2,3] augmented with GD3BJ dispersion correction model [4]. Molecular geometry of the singlet ground state of the compounds was optimized in the gas phase and the frequency calculation for each of the compounds shows only positive values which verify that the optimized molecular structure corresponds to energy minimum. Solvent effect was taken into account using polarizable continuum model (PCM) [5] with dichloromethane and chloroform as solvents. Such calculations were carried out for analysis of the frontier molecular orbitals

structure, energy levels and UV-Vis data. Density of states diagrams were obtained with use of GaussSum program (2.2, city, country) [6]. The TD-DFT (time dependent density functional theory) method [7] was employed to calculate the electronic absorption spectra of the compounds in solvents and geometries and energies in singlet/triplet excited states.

1.5. OLED preparation and electroluminescence measurements

Devices with sandwich configuration ITO/PEDOT:PSS/AzNI/Al and ITO/PEDOT:PSS/AzNI:PVK:PBD/Al with 2 and 15 wt. % of AzNIs content in blend were prepared. Devices were prepared on OSSILA substrates with pixilated ITO anodes, cleaned with detergent, deionized water, 10% NaOH solution, water and isopropyl alcohol in an ultrasonic bath. Substrates were covered with PEDOT:PSS film by spin coating at 5000rpm for 60s and annealed for 5 min at 120°C. Active layer was spin-coated on top of the PEDOT:PSS layer from chloroform solution (10 mg/mL) at 1000 rpm for 60 s and annealed for 5 min at 100°C. Finally, Al was vacuum-deposited at a pressure of $5 \cdot 10^{-5}$ Torr. Electroluminescence (EL) spectra were measured with the voltage applied using a precise voltage supply (GwInstek PSP-405, Taipei, Taiwna) and the sample was fixed to an XYZ stage. Light from the OLED device was collected through a 30mm lens, focused on the entrance slit (50µm) of a monochromator (Shamrock SR-303i, Andor Technology, Belfast, Ireland) and detected using a CCD detector (AndoriDus 12305, Oxford Instruments, London). Typical acquisition times were equal to 10 s. The pre-alignment of the setup was done using a 405 nm laser. At this stage, the diode parameters (luminance, luminous efficacy) were not measured.

2. NMR spectra

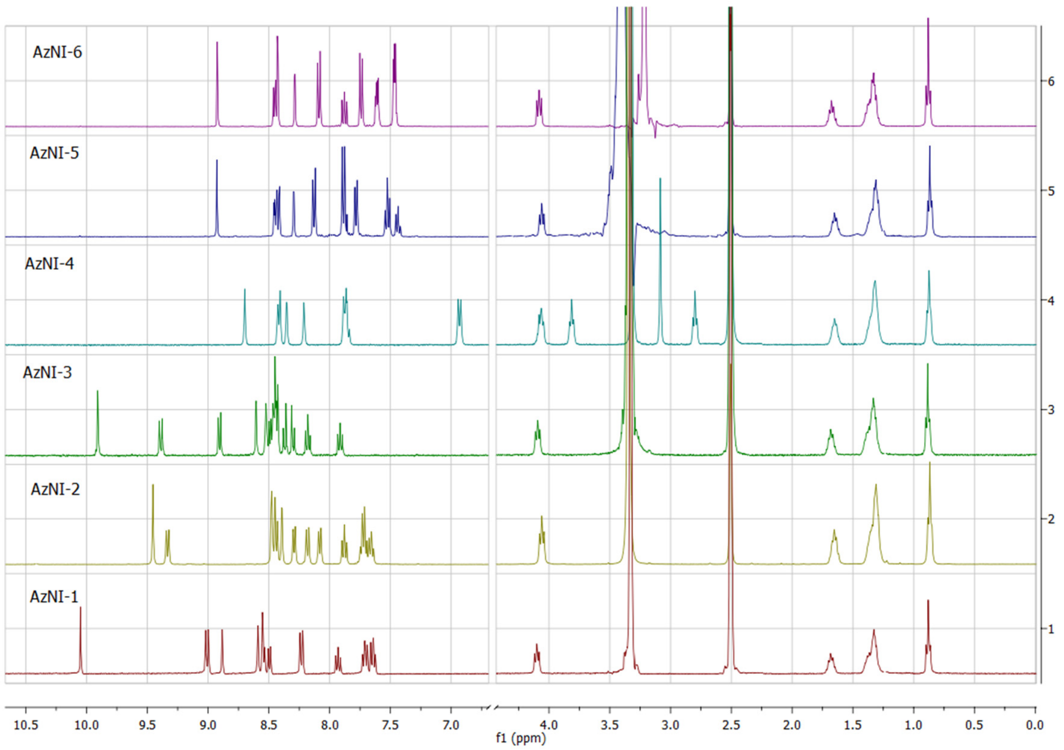

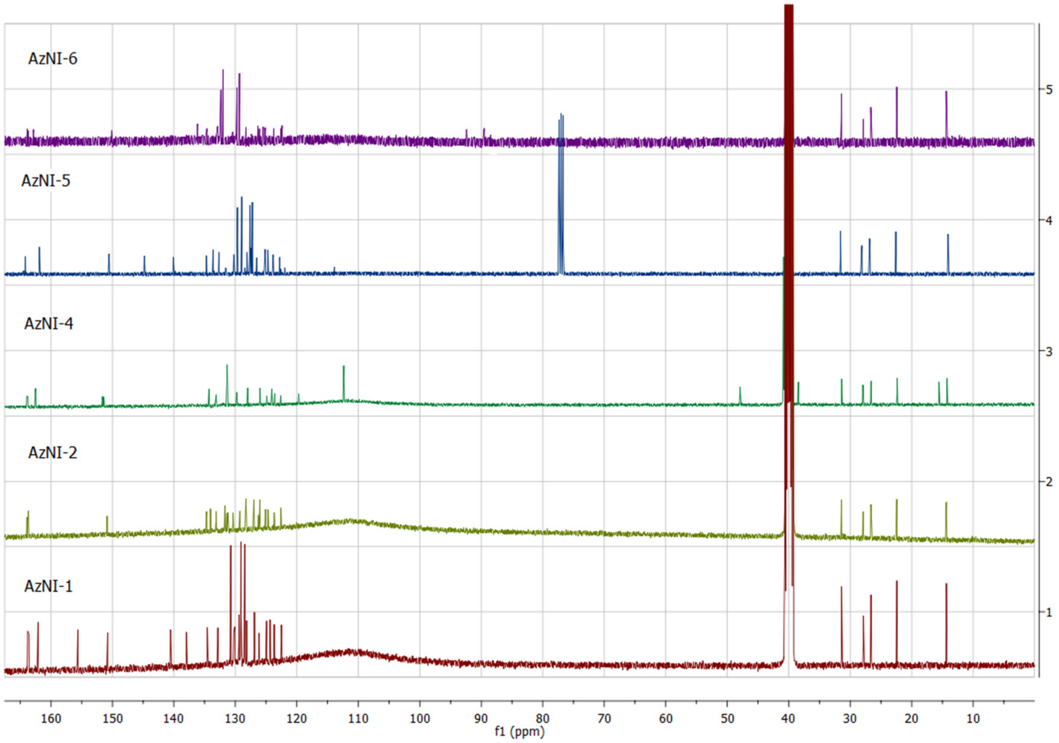

Figure S1. ^{1}H NMR(DMSO-d$_6$, 400MHz) and ^{13}C NMR spectra (DMSO-d$_6$ or CDCl$_3$, 101MHz).

3. Photographs of the solid state of AzNIs

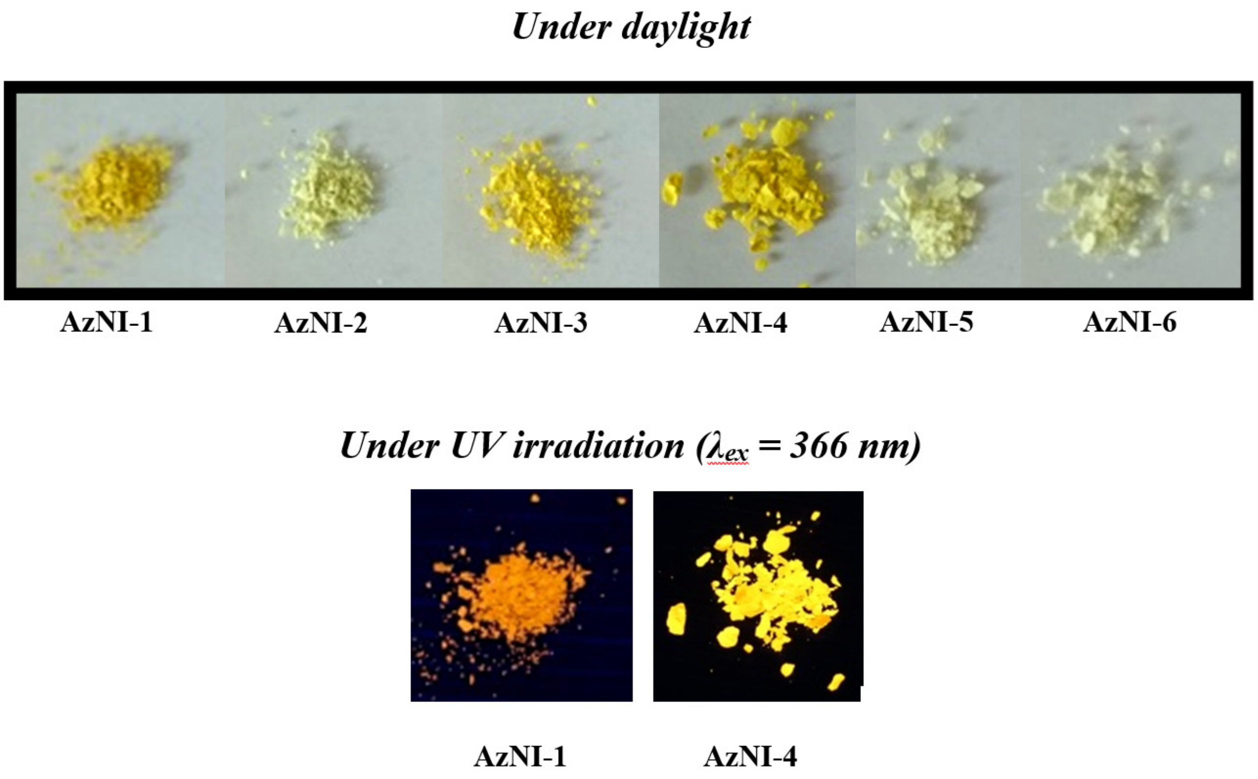

Figure S2. Photographs of the solid state of AzNIs under daylight and under UV irradiation.

4. Thermal investigations

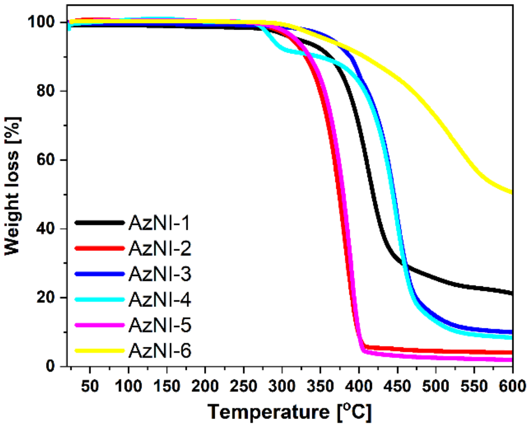

(a)

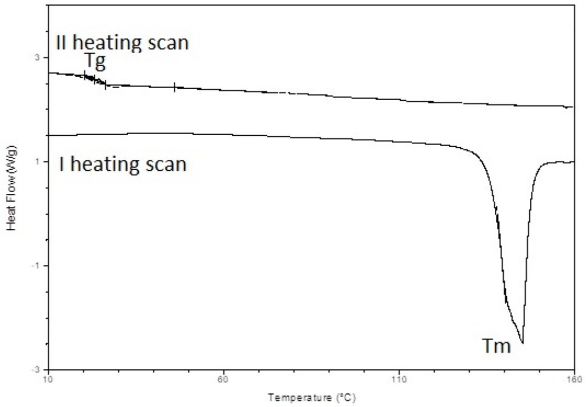

(b)

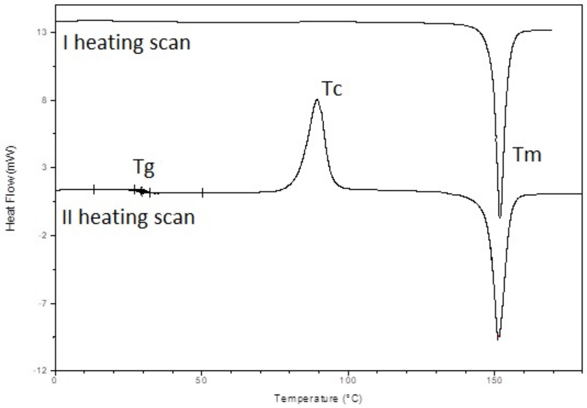

(c)

Figure S3. (**a**) TGA thermograms and DSC thermograms of (**b**) AzNI-5 and (**c**) AzNI-6.

5. Density functional theory calculations

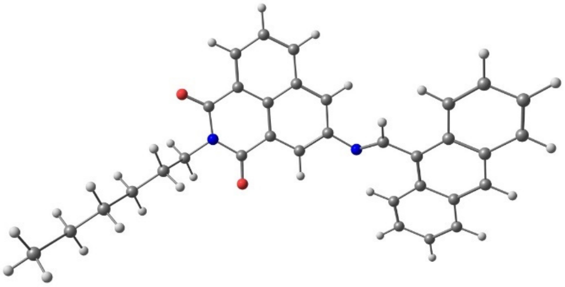

AzNI-1

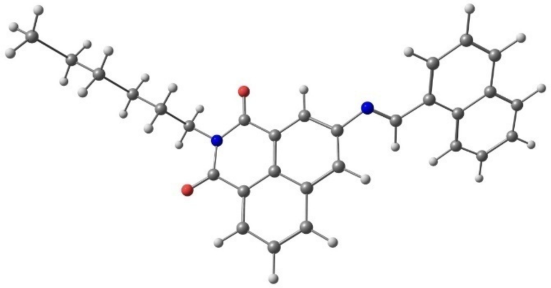

AzNI-2

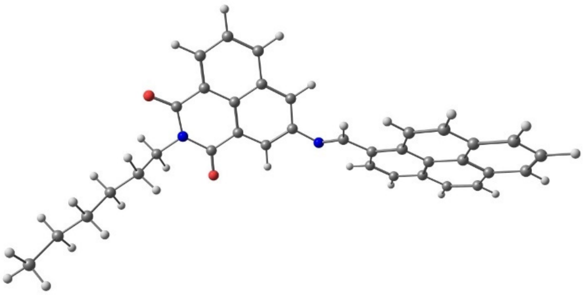

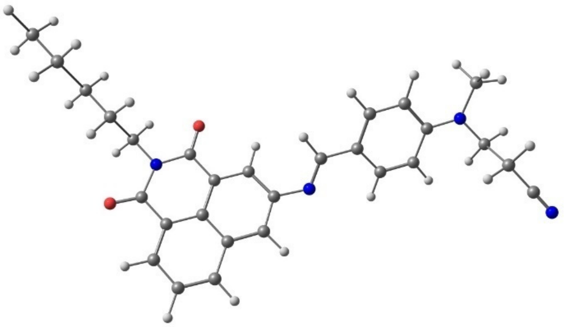

AzNI-3 AzNI-4

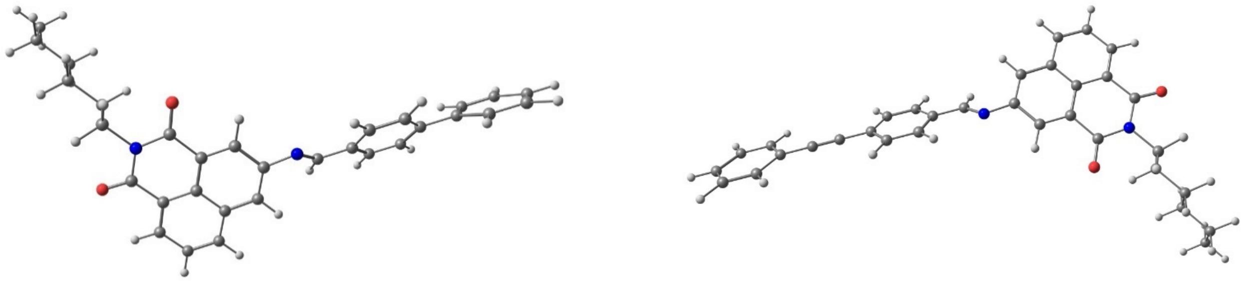

AzNI-5 AzNI-6

Figure S4. Optimized geometries of the compounds AzNI.

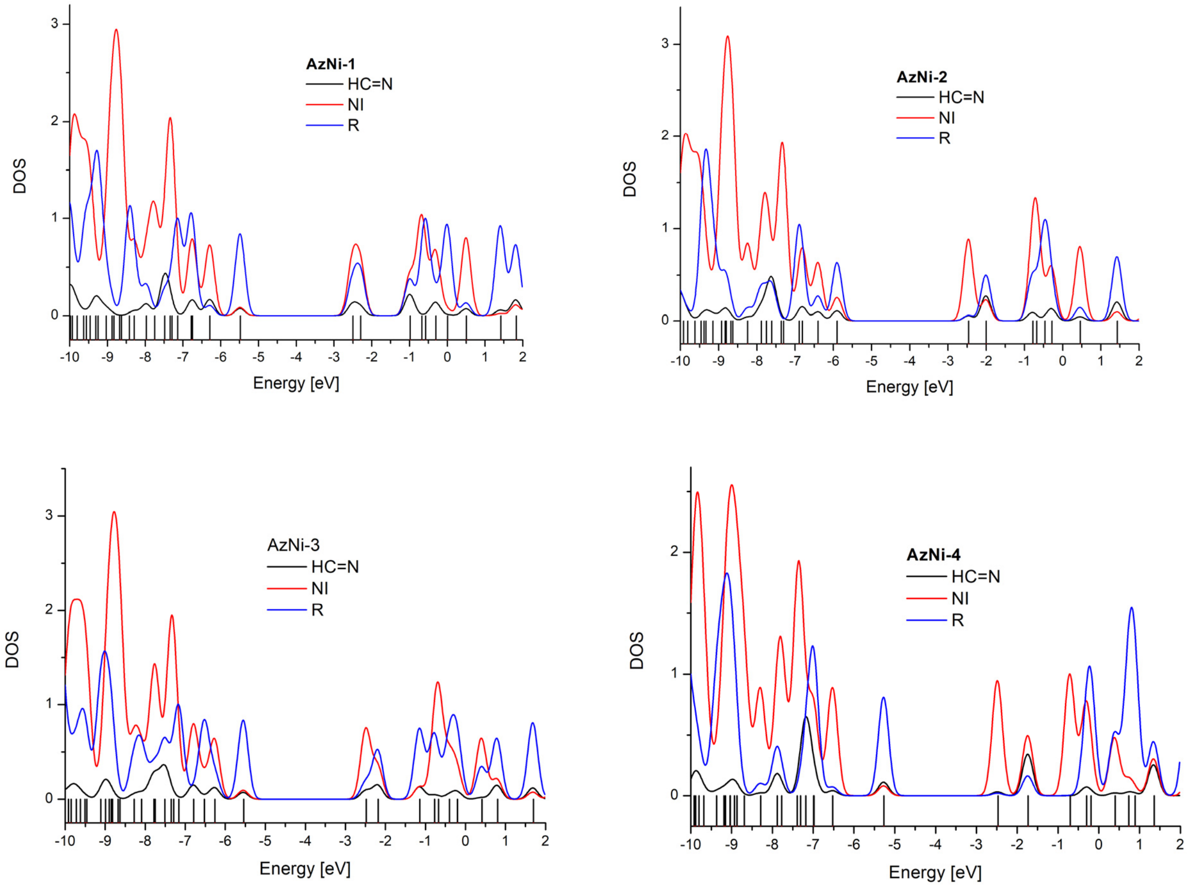

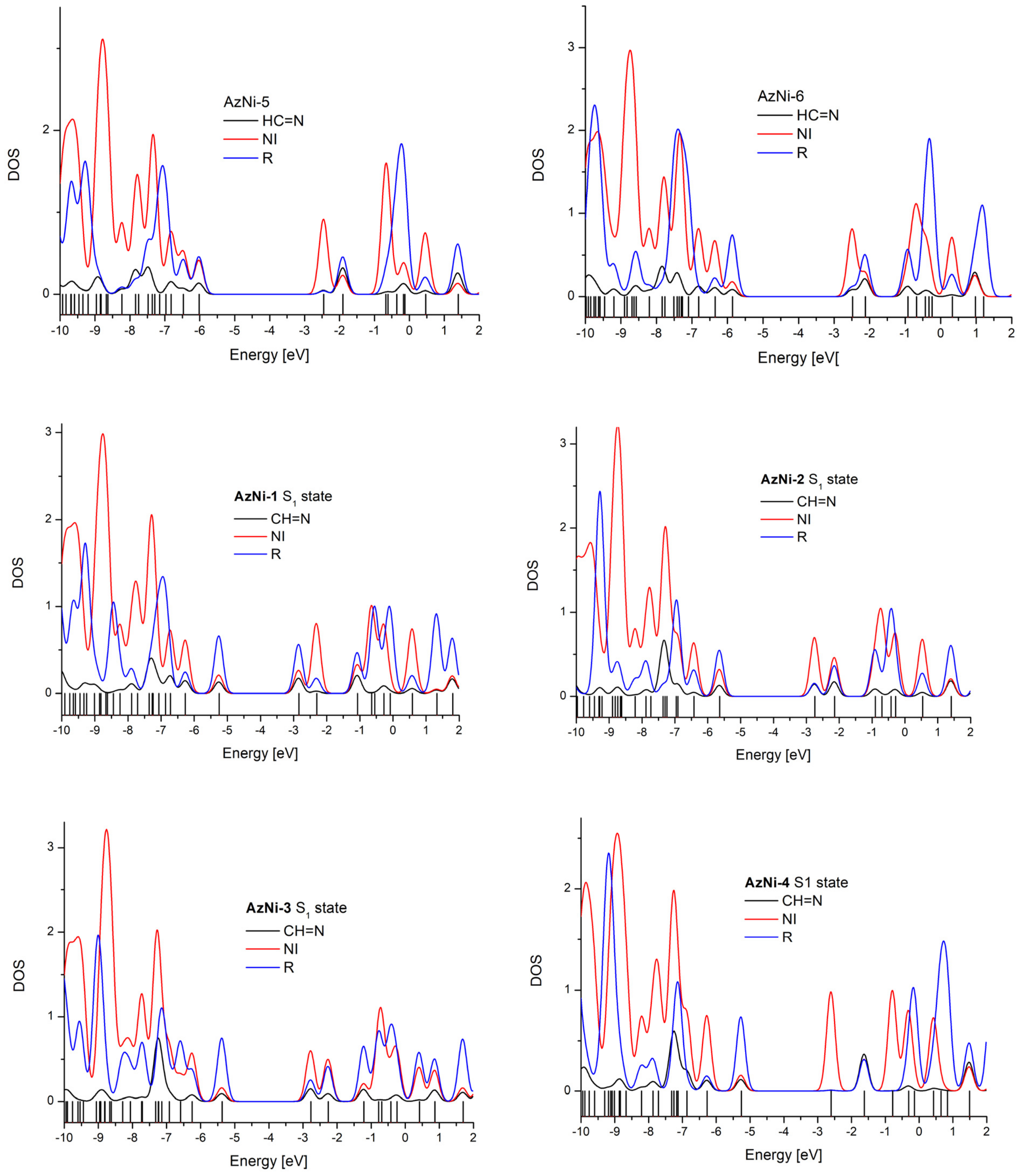
AzNi-5
HC=N
NI
R
DOS
Energy [eV]
AzNi-6
HC=N
NI
R
DOS
Energy [eV[
AzNi-1 S1 state
CH=N
NI
R
DOS
Energy [eV]
AzNi-2 S1 state
CH=N
NI
R
DOS
Energy [eV]
AzNi-3 S1 state
CH=N
NI
R
DOS
Energy [eV]
AzNi-4 S1 state
CH=N
NI
R
DOS
Energy [eV]

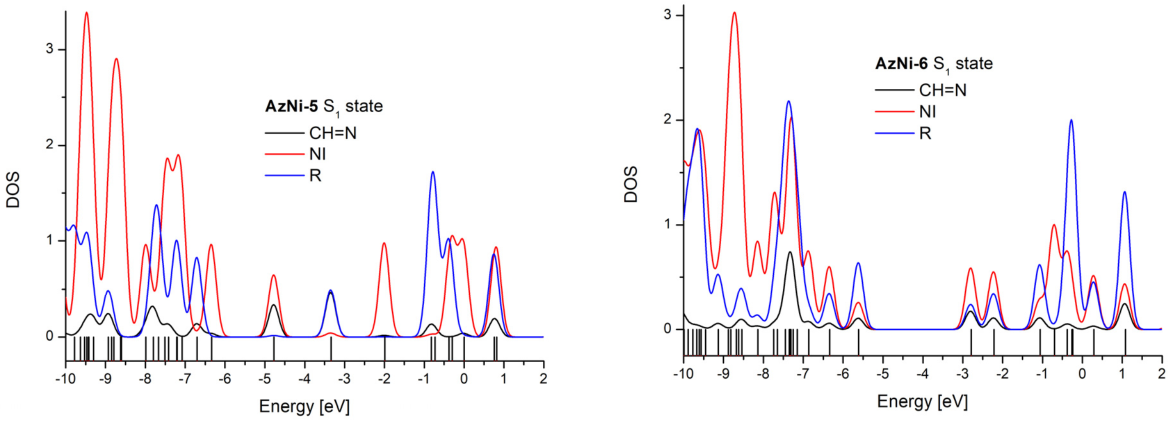

Figure S5. Density-of-states diagrams. NI: N-hexyl-1,8-naphthalimide; R: 9-anthracene, 1-naphthalene, 1-pyrene, 4-[(2-cyanoethyl)methylamino]-4-benzene, 4-phenyl-benzene, 4-(2-phenyleth-1-ynyl)benzene in AzNI-1, AzNI-2, AzNI-3, AzNI-4, AzNI-5, and AzNI-6, respectively.

Table S1. Ground and S_1 state composition of selected molecular orbitals.

GS

**AzNI-1**	eV	CH=N	NI	R	**AzNI-2**	eV	CH=N	NI	R
L+5	−0.30	14	66	20	L+5	−0.28	13	58	29
L+4	−0.57	0	2	98	L+4	−0.46	1	3	96
L+3	−0.67	0	100	0	L+3	−0.68	0	99	0
L+2	−0.98	22	41	37	L+2	−0.78	9	43	48
L+1	−2.29	8	50	42	L+1	−2.00	27	23	50
LUMO	−2.50	11	55	34	LUMO	−2.46	5	89	6
HOMO	−5.48	7	8	84	HOMO	−5.90	11	25	64
H-1	−6.29	17	73	10	H-1	−6.40	10	63	27
H-2	−6.75	14	67	19	H-2	−6.81	15	79	6
H-3	−6.78	2	12	86	H-3	−6.89	0	0	100
H-4	−7.14	0	3	97	H-4	−7.30	0	100	0
H-5	−7.29	0	100	0	H-5	−7.36	2	98	0
**AzNI-3**	eV	CH=N	NI	R	**AzNI-4**	eV	CH=N	NI	R
L+5	−0.40	4	39	57	L+5	0.41	2	73	25
L+4	−0.67	0	100	0	L+4	−0.25	0	9	91
L+3	−0.77	4	27	68	L+3	−0.36	5	74	21
L+2	−1.14	13	13	74	L+2	−0.64	0	100	0
L+1	−2.18	15	34	52	L+1	−1.61	38	29	33
LUMO	−2.48	9	73	18	LUMO	−2.40	2	97	1
HOMO	−5.54	7	9	84	HOMO	−5.46	10	15	74
H-1	−6.26	12	62	26	H-1	−6.28	14	70	16
H-2	−6.52	2	18	80	H-2	−6.83	12	83	5
H-3	−6.79	15	78	8	H-3	−7.10	0	0	99
H-4	−7.16	1	0	99	H-4	−7.27	7	91	2
H-5	−7.29	0	100	0	H-5	−7.36	5	95	0
**AzNI-5**	eV	CH=N	NI	R	**AzNI-6**	eV	CH=N	NI	R
L+5	−0.17	5	14	80	L+5	−0.33	3	26	72
L+4	−0.36	1	2	97	L+4	−0.43	5	43	52
L+3	−0.61	3	66	31	L+3	−0.68	0	100	0
L+2	−0.67	0	99	0	L+2	−0.92	12	31	57
L+1	−1.90	32	23	45	L+1	−2.12	21	28	50
LUMO	−2.45	5	91	4	LUMO	−2.48	8	81	11
HOMO	−6.02	13	41	46	HOMO	−5.86	8	18	74

H-1	−6.47	7	51	42	H-1	−6.35	10	67	22
H-2	−6.82	15	75	10	H-2	−6.81	12	82	6
H-3	−6.97	0	0	100	H-3	−7.10	0	0	100
H-4	−7.15	0	0	100	H-4	−7.27	1	1	98
H-5	−7.29	0	100	0	H-5	−7.30	0	98	2

S_1 state

**AzNI-1**	eV	CH=N	NI	R	**AzNI-2**	eV	CH=N	NI	R
L+5	−0.27	8	77	15	L+5	−0.28	8	71	21
L+4	−0.55	0	2	98	L+4	−0.42	1	7	92
L+3	−0.64	0	99	1	L+3	−0.70	1	92	7
L+2	−1.07	20	33	47	L+2	−0.90	8	38	54
L+1	−2.30	2	80	17	L+1	−2.14	17	46	36
LUMO	−2.84	17	26	56	LUMO	−2.74	14	70	15
HOMO	−5.25	13	21	66	HOMO	−5.64	13	32	55
H-1	−6.27	14	61	24	H-1	−6.42	5	64	31
H-2	−6.72	20	72	8	H-2	−6.91	13	62	25
H-3	−6.87	0	0	100	H-3	−6.96	1	7	91
H-4	−7.07	10	9	81	H-4	−7.24	0	100	0
H-5	−7.24	1	98	1	H-5	−7.30	38	57	5
**AzNI-3**	eV	CH=N	NI	R	**AzNI-4**	eV	CH=N	NI	R
L+5	−0.42	1	25	74	L+5	0.43	2	71	27
L+4	−0.69	1	83	16	L+4	−0.14	0	5	95
L+3	−0.78	1	31	68	L+3	−0.31	5	78	18
L+2	−1.21	14	21	65	L+2	−0.78	0	100	0
L+1	−2.26	9	50	41	L+1	−1.62	37	32	32
LUMO	−2.77	15	60	25	LUMO	−2.60	1	99	1
HOMO	−5.37	9	16	75	HOMO	−5.26	11	15	73
H-1	−6.25	7	57	36	H-1	−6.27	11	75	15
H-2	−6.59	1	29	70	H-2	−6.87	19	74	7
H-3	−6.92	9	63	28	H-3	−7.13	2	1	97
H-4	−7.14	3	3	94	H-4	−7.17	15	79	6
H-5	−7.23	11	87	2	H-5	−7.25	36	59	6
**AzNI-5**	eV	CH=N	NI	R	**AzNI-6**	eV	CH=N	NI	R
L+5	−0.30	0	96	4	L+5	−0.26	1	20	79
L+4	−0.38	0	3	97	L+4	−0.38	4	58	37
L+3	−0.73	3	1	96	L+3	−0.70	0	97	2
L+2	−0.82	11	2	87	L+2	−1.06	11	27	62
L+1	−1.99	2	98	0	L+1	−2.22	11	55	34
LUMO	−3.34	47	4	49	LUMO	−2.79	17	59	24
HOMO	−4.77	34	65	2	HOMO	−5.61	11	26	64
H-1	−6.34	4	96	0	H-1	−6.34	6	60	34
H-2	−6.70	14	3	83	H-2	−6.86	7	74	19
H-3	−7.08	0	100	0	H-3	−7.15	0	0	100
H-4	−7.20	0	98	2	H-4	−7.25	1	99	0
H-5	−7.21	0	2	98	H-5	−7.31	63	16	22

NI: N-hexyl-1,8-naphthalimide; R: 9-anthracene, 1-naphthalene, 1-pyrene, 4-[(2-cyanoethyl)methylamino]-4-benzene, 4-phenyl-benzene, 4-(2-phenyleth-1-ynyl)benzene in AzNI-1, AzNI-2, AzNI-3, AzNI-4, AzNI-5, and AzNI-6, respectively.

Table S2. Calculated, in the chloroform solution, dipole moments and mean plane angles for the imine molecules.

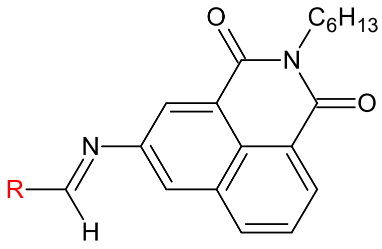

NI

	Dipole moment (D)	∠ NI-R (°)		Dipole moment (D)	∠ NI-R (°)
AzNI-1			AzNI-4		
S_0	7.30	28.72	S_0	5.97	35.51
S_1	8.59	32.67	S_1	7.00	22.61
T_1	9.02	30.74	T_1	4.49	53.76
AzNI-2			AzNI-5		
S_0	7.12	47.71	S_0	7.19	44.38
S_1	7.93	10.48	S_1	15.84	85.67
T_1	8.40	2.23	T_1	8.25	0.52
AzNI-3			AzNI-6		
S_0	8.01	45.45	S_0	7.28	43.85
S_1	8.98	6.15	S_1	8.05	6.06
T_1	9.82	3.15	T_1	8.43	1.88

Table S3. The calculated electronic transitions corresponding to excitation wavelength in $CHCl_3$ solution.

	⚜$_{exp}$	⚜$_{calc}$ (*f*)	transition	character
**AzNI-1**	411	437.7 (0.2159)	HOMO→L+1 (97%)	◎$_{R}$→◎$^{*}_{R/NI}$
	331	336.1 (0.1660)	H-3→LUMO (39%); H-2→LUMO (30%)	◎$_{R/NI}$→◎$^{*}_{R/NI}$
**AzNI-2**	338	332.4 (0.1637)	H-2→LUMO (70%)	◎$_{NI}$→◎$^{*}_{NI}$
**AzNI-3**	405	406.4 (0.7709)	HOMO→L+1 (92%)	◎$_{R}$→◎*
	381	368.6 (0.1012)	H-1→LUMO (77%)	◎$_{NI}$→◎$^{*}_{NI}$
**AzNI-4**	349	360.9 (0.9193)	HOMO→L+1 (94%)	◎$_{R}$→◎$^{*}_{R/HC=N}$
		355.5 (0.1237)	H-1→LUMO (93%)	◎$_{NI}$→◎$^{*}_{NI}$
**AzNI-5**	340	349.8 (0.6645)	HOMO→L+1 (84%)	◎ →◎*
	323	327.9 (0.1928)	H-2→LUMO (82%)	◎$_{NI}$→◎$^{*}_{NI}$
**AzNI-6**	335	337.2 (0.1928)	H-2→LUMO (48%); H-1→L+1 (42%)	◎$_{NI}$→◎$^{*}_{NI}$

Table S4. Energy differences between excited states.

	AzNI-1	AzNI-2	AzNI-3	AzNI-4	AzNI-5	AzNI-6
◎E$_{(S0\text{-}S1)}$(nm)	534 (511)*	452 (511)	504 (509)	514 (523)	581 (511)	460 (508)
◎E$_{(T1\text{-}S0)}$(cm^{-1})	4399	12731	11870	6539	13943	13313
◎E$_{(T1\text{-}S1)}$(cm^{-1})	2806	2805	3242	3975	3268	3134
◎E$_{(T2\text{-}S1)}$(cm^{-1})	3146	4686	7959	1940	1875	4107
◎E$_{(T2\text{-}T1)}$(cm^{-1})	11173	4690	8872	10322	5143	4324

* in parenthesis are given experimental values of emission maxima.

6. Optical properties

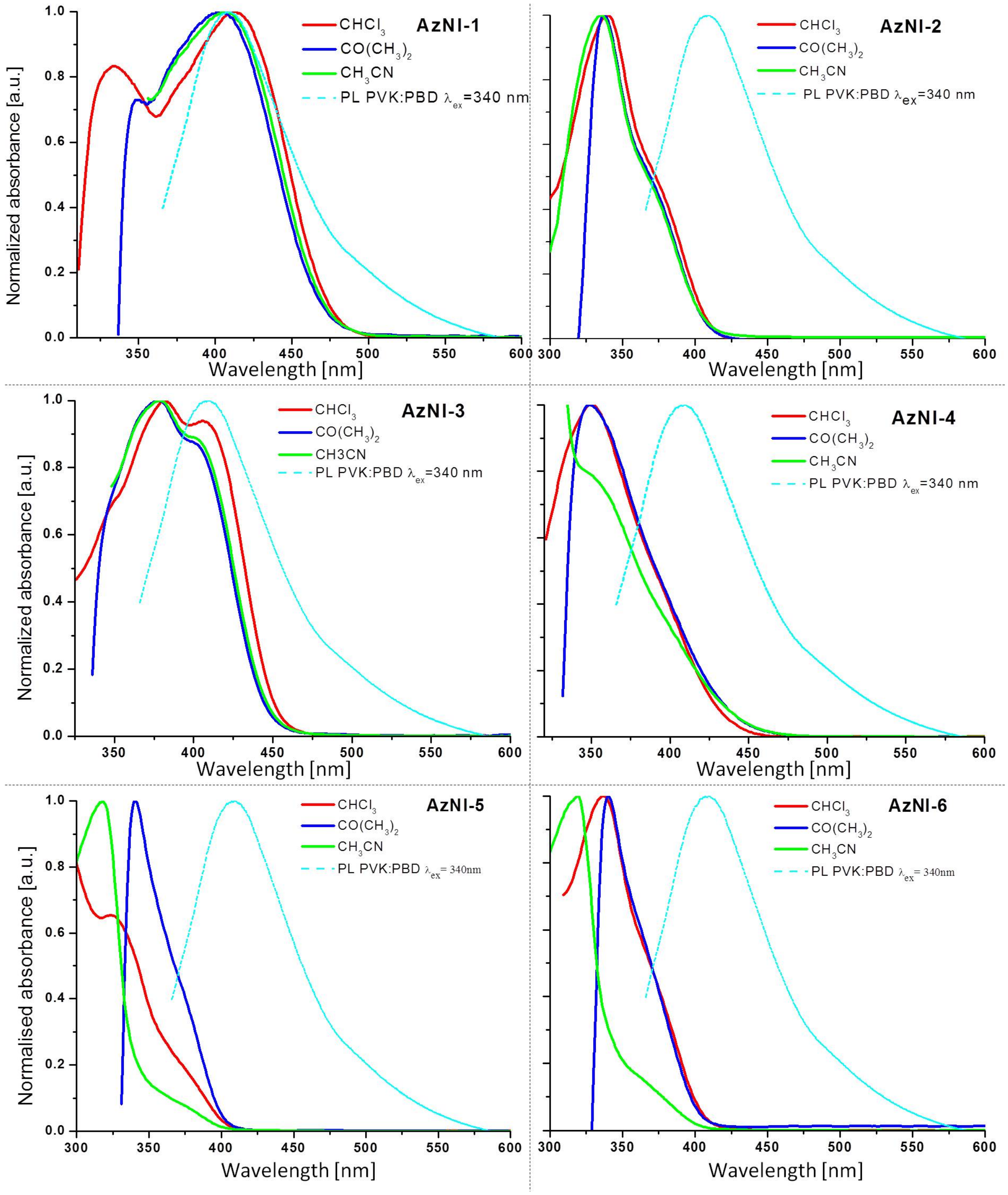

Figure S6. The absorption spectra of the *N*-hexyl-1,8-naphthalimides derivatives (AzNI-1 - 6) in the various solvents with the emission spectra of the PVK:PBD matrix.

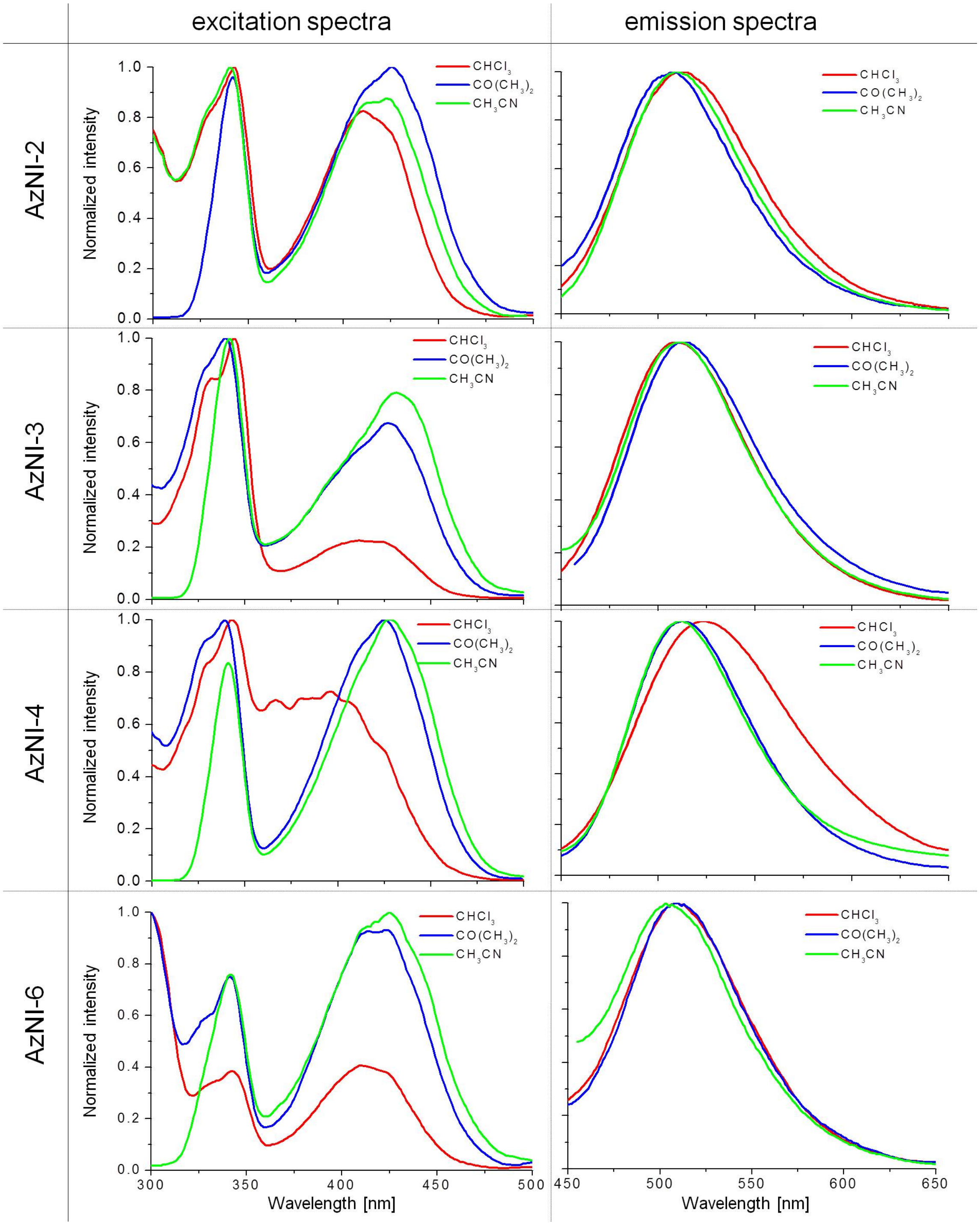

Figure S7. The excitation and emission spectra of the *N*-hexyl-1,8-naphthalimides derivatives (AzNI- 2, 3, 4, 6) in the various solvents (λ_{ex} = 340 nm).

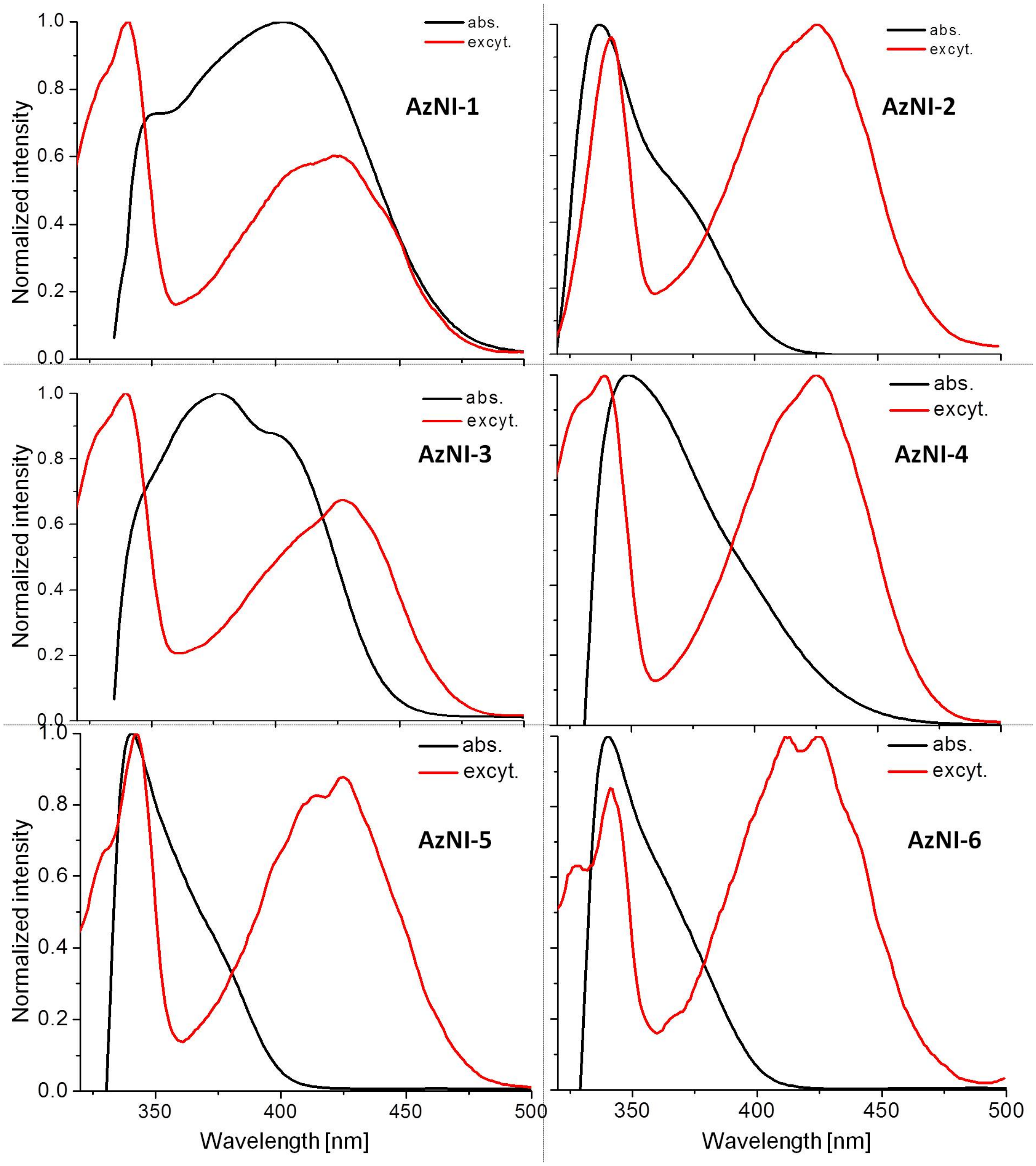

Figure S8. The absorption and excitation spectra of the *N*-hexyl-1,8-naphthalimides derivatives (AzNI- 1- 6) in acetone.

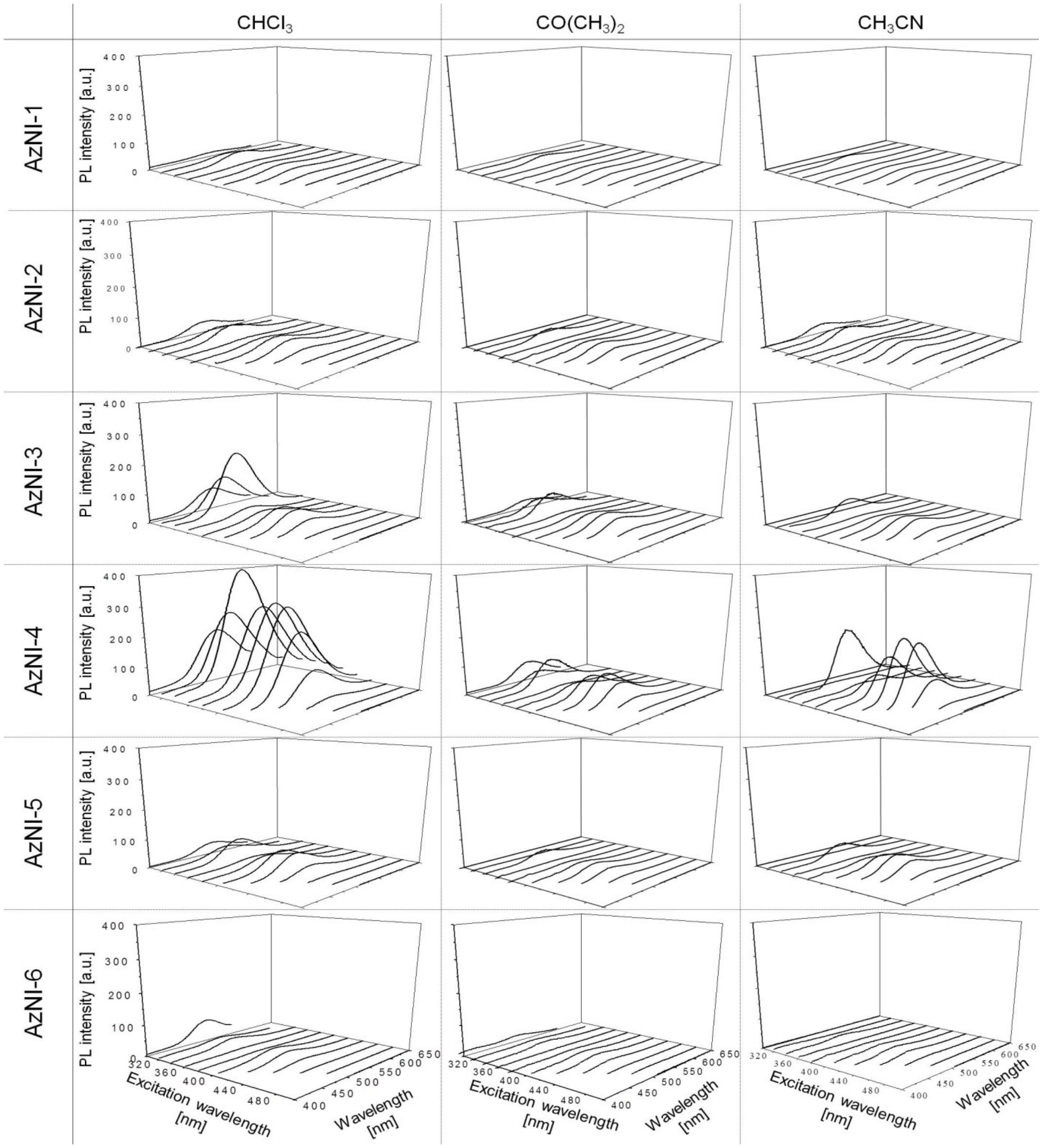

Figure S9. The 3D fluorescence spectra of the analyzed compounds in the excitation range from 300 to 500 nm and the collected emissions in the range from 400 to 650 nm in the chloroform ($CHCl_3$), acetone ($CO(CH_3)_2$) and acetonitryle (CH_3CN). Measurements were performed for equal concentration of each compound ($c = 1 \cdot 10^{-5}$ mol/dm^3) and under the same measurement conditions.

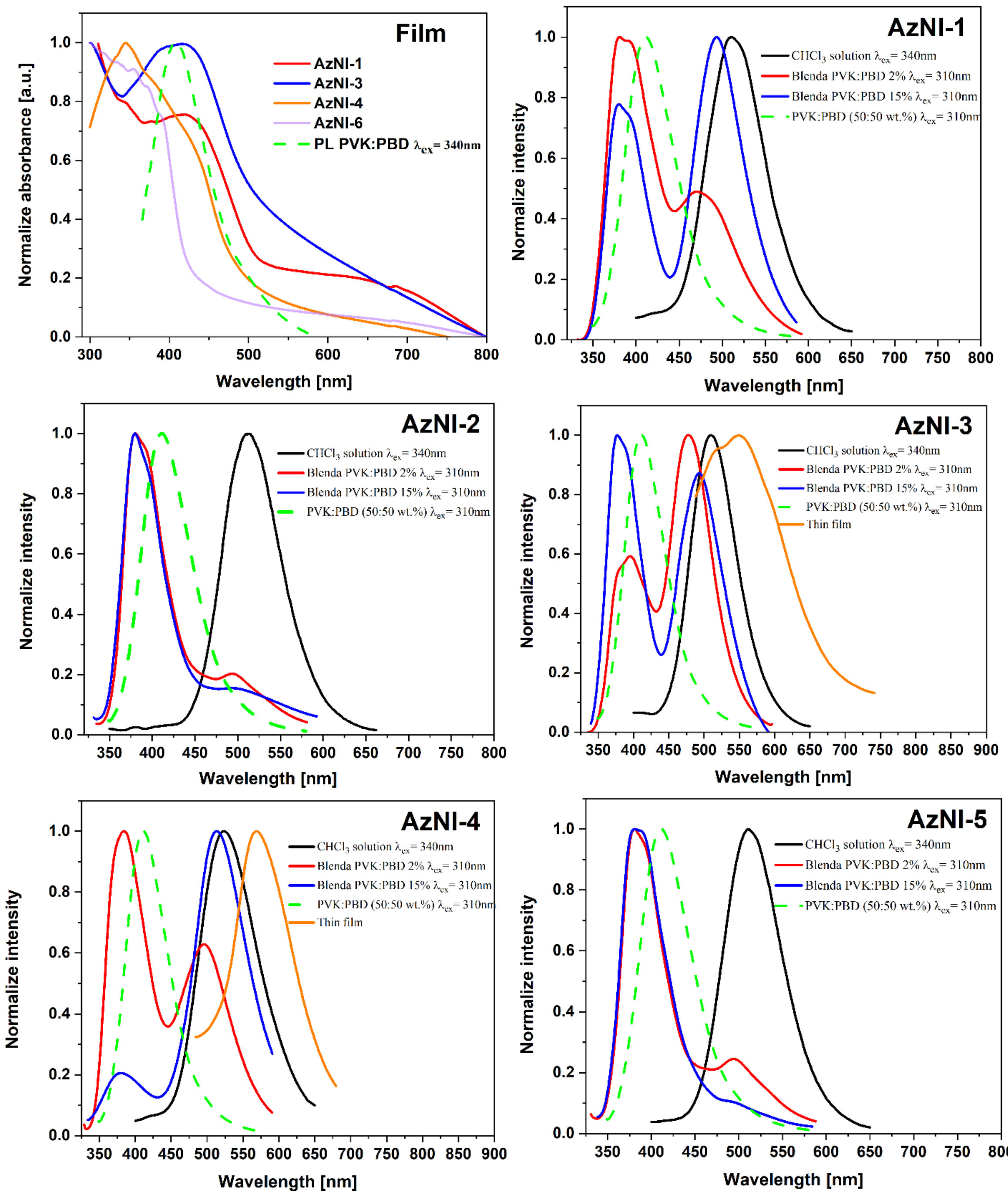
Film
AzNI-1
AzNI-3
AzNI-4
AzNI-6
PL PVK:PBD λex= 340nm
Normalize absorbance [a.u.]
Wavelength [nm]
AzNI-1
CHCl3 solution λex= 340nm
Blenda PVK:PBD 2% λex= 310nm
Blenda PVK:PBD 15% λex= 310nm
PVK:PBD (50:50 wt.%) λex= 310nm
Normalize intensity
Wavelength [nm]
AzNI-2
CHCl3 solution λex= 340nm
Blenda PVK:PBD 2% λex= 310nm
Blenda PVK:PBD 15% λex= 310nm
PVK:PBD (50:50 wt.%) λex= 310nm
Normalize intensity
Wavelength [nm]
AzNI-3
CHCl3 solution λex= 340nm
Blenda PVK:PBD 2% λex= 310nm
Blenda PVK:PBD 15% λex= 310nm
PVK:PBD (50:50 wt.%) λex= 310nm
Thin film
Normalize intensity
Wavelength [nm]
AzNI-4
CHCl3 solution λex= 340nm
Blenda PVK:PBD 2% λex= 310nm
Blenda PVK:PBD 15% λex= 310nm
PVK:PBD (50:50 wt.%) λex= 310nm
Thin film
Normalize intensity
Wavelength [nm]
AzNI-5
CHCl3 solution λex= 340nm
Blenda PVK:PBD 2% λex= 310nm
Blenda PVK:PBD 15% λex= 310nm
PVK:PBD (50:50 wt.%) λex= 310nm
Normalize intensity
Wavelength [nm]

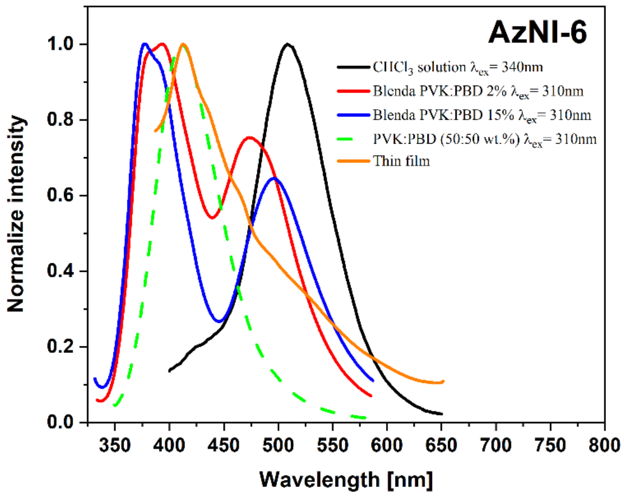

Figure S10. Normalize absorption spectra of a thin films and emission spectra in solutions and blends PVK:PBD:AzNI (and selected a thin films) with emission of the matrix.

Table S5. Life-time measurements with a time-correlated single photon counting (TCSPC) method.

Code	Medium	τ_1(ns)	τ_2(ns)	τ_3(ns)	$\Sigma\tau$(ns)	χ^2
AzNI-1		3.0	11.0	-	14.01	0.986
AzNI-2		0.2	9.6	-	9.81	1.029
AzNI-3	$CHCl_3$	0.1	9.3	-	9.48	1.159
AzNI-4		0.2	1.1	6.7	8.25	1.028
AzNI-5		0.6	10.3	-	10.89	1.069
AzNI-6		4.4	11.1	-	15.68	1.069

Pulse period = 50 ns. Results were presented as average values of decay after exponential fitting.
χ^2- coefficient of determination.

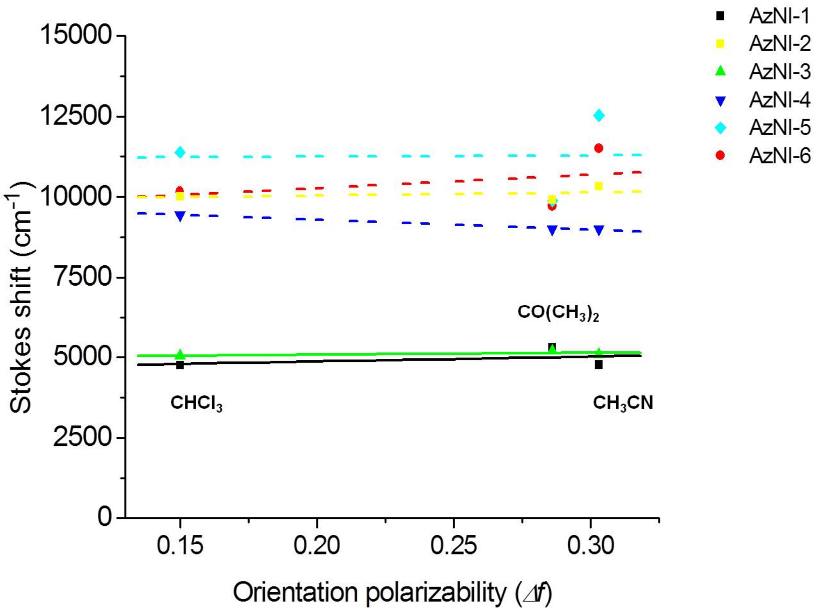

Figure S11. The dependence of the Stokes Shift on solvent orientation polarizability Δf. Stokes shifts calculated according to the equation $\Delta\nu=(1/\lambda_{abs}-1/\lambda_{em})\cdot 10^7$ [cm^{-1}]. Δf stands for the orientation polarizability defined as: $\Delta f=[(\varepsilon-1)/(2\varepsilon-1)]-[(n^2-1)/(2n^2-1)]$,where ε and n are the dielectric constants and the refractive index of the solvent, respectively.

Table S6. The electrochemical band gap and the optical band gap.

Molecule	Band gap (eV)	CV method	$CHCl_3$	$CO(CH_3)_2$	CH_3CN
**AzNI−1**	E_g	1.92[a] 2.12[b]	-	-	-
	E_g^{opt}	-	2.61	2.56	2.53
**AzNI−2**	E_g	2.41[a] 2.26[b]	-	-	-
	E_g^{opt}	-	2.99	3.02	3.02
**AzNI−3**	E_g	2.14[a] 2.36[b]	-	-	-
	E_g^{opt}	-	2.70	2.73	2.71
**AzNI−4**	E_g	1.97[a] 1.87[b]	-	-	-
	E_g^{opt}	-	2.79	2.76	2.76
**AzNI−5**	E_g	2.28	-	-	-
	E_g^{opt}	-	3.01	3.04	3.07
**AzNI−6**	E_g	2.25[a] 2.20[b]	-	-	-
	E_g^{opt}	-	3.02	3.04	3.06

[a]–DCM, [b]– ACN

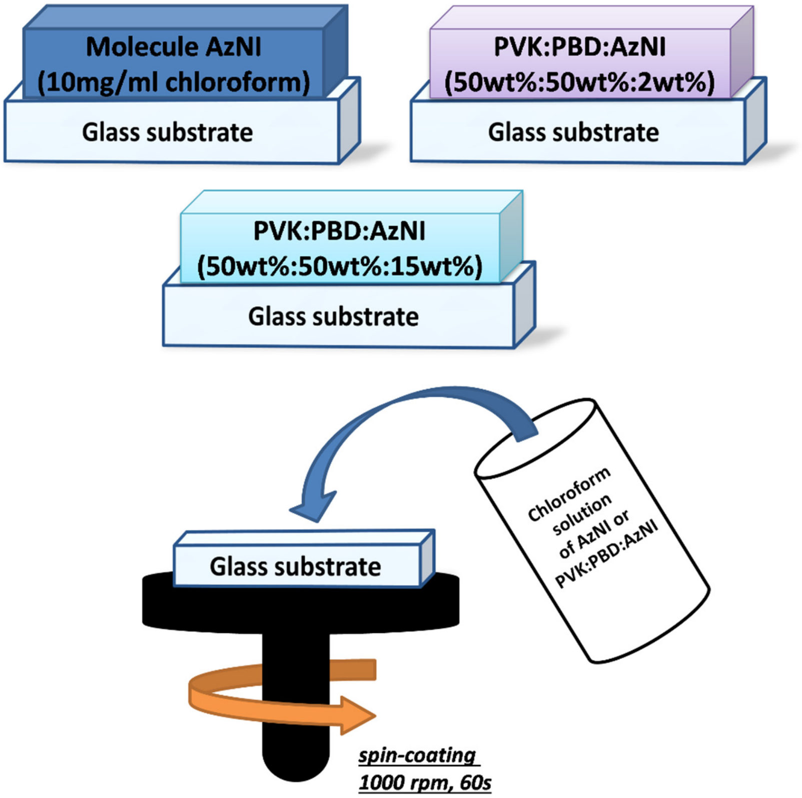

Figure S12. The demonstrative drawing of obtaining thin layers on the glass substrate.

7. Optimized geometries of the compounds in ground and excited states

Ground state

AzNI-1

C	-1.578280000	0.552602000	-0.804180000
C	-1.651581000	1.962006000	-0.666734000
C	-0.357180000	-0.085072000	-0.828105000
C	-0.446942000	2.717836000	-0.554673000
C	0.848455000	0.649910000	-0.684600000
C	0.792589000	2.033166000	-0.564916000
C	-2.902421000	2.621866000	-0.649806000
C	-2.963302000	3.998212000	-0.522374000
C	-0.546292000	4.128396000	-0.435131000
C	-1.777288000	4.751841000	-0.416453000
C	-2.819260000	-0.250278000	-0.930123000
C	-4.158293000	1.843551000	-0.765925000
N	-4.028625000	0.458193000	-0.939353000
O	-2.809027000	-1.470457000	-1.024471000
O	-5.264639000	2.365788000	-0.720803000
N	2.051562000	-0.063963000	-0.737225000
C	2.990490000	0.241207000	0.087728000
C	4.324653000	-0.355516000	0.145095000
C	3.731662000	-2.352113000	-1.274956000
C	4.648064000	-1.599766000	-0.477456000
C	4.093085000	-3.553235000	-1.832520000

C	5.963497000	-2.162921000	-0.282573000
C	5.390459000	-4.097151000	-1.641099000
C	6.299795000	-3.414430000	-0.881605000
C	5.313891000	0.336571000	0.910758000
C	6.901242000	-1.484343000	0.497159000
C	6.617818000	-0.254628000	1.091514000
C	5.109692000	1.624154000	1.502863000
C	6.087395000	2.244842000	2.237368000
C	7.610148000	0.420222000	1.865756000
C	7.356015000	1.637238000	2.433723000
H	2.786517000	0.998559000	0.850710000
H	-0.319722000	-1.161766000	-0.944723000
H	1.713301000	2.606465000	-0.515206000
H	-3.937334000	4.473815000	-0.509320000
H	0.366149000	4.712237000	-0.354269000
H	-1.836770000	5.831074000	-0.320644000
H	2.745778000	-1.946494000	-1.443401000
H	3.371388000	-4.097463000	-2.434142000
H	5.652565000	-5.047870000	-2.094418000
H	7.298009000	-3.809489000	-0.716045000
H	7.884892000	-1.924520000	0.640655000
H	4.174896000	2.148833000	1.357831000
H	5.891237000	3.222007000	2.667798000
H	8.577385000	-0.059287000	1.985545000
H	8.115277000	2.144965000	3.019485000
C	-5.268724000	-0.326692000	-1.049354000
H	-5.041602000	-1.193248000	-1.670596000
H	-6.000868000	0.305825000	-1.551680000
C	-5.790174000	-0.768786000	0.317979000
H	-5.015286000	-1.363871000	0.814975000
H	-5.966646000	0.121726000	0.932332000
C	-7.079010000	-1.585175000	0.203461000
H	-6.898080000	-2.465746000	-0.428445000
H	-7.843872000	-0.988829000	-0.313103000
C	-7.622782000	-2.039315000	1.560350000
H	-6.856820000	-2.633821000	2.077608000
H	-7.801662000	-1.158480000	2.192677000
C	-8.912877000	-2.857339000	1.455671000
H	-8.732209000	-3.736562000	0.822926000
H	-9.676403000	-2.262039000	0.937313000
C	-9.448930000	-3.306168000	2.816572000
H	-8.716978000	-3.929689000	3.342120000
H	-9.667659000	-2.444627000	3.457384000
H	-10.370375000	-3.888126000	2.714401000

AzNI-2

C	-6.097349000	-0.612918000	-0.163552000
C	-6.295074000	0.643606000	-0.798101000
C	-7.246806000	-1.450882000	0.031901000
C	-7.545582000	1.048583000	-1.207063000
C	-8.522640000	-1.001927000	-0.401087000
C	-8.674383000	0.221429000	-1.007392000
C	-4.819808000	-1.092624000	0.288206000
C	-4.725408000	-2.341539000	0.887818000

C	-7.093784000	-2.716397000	0.651232000
C	-5.856513000	-3.154990000	1.068682000
C	-3.604317000	-0.288690000	0.137734000
C	1.013793000	0.334515000	-0.182444000
C	0.997965000	1.697439000	0.208675000
C	-0.122615000	-0.437082000	-0.073780000
C	-0.207552000	2.266189000	0.717044000
C	-1.338972000	0.125138000	0.393732000
C	-1.365818000	1.455205000	0.794630000
C	2.167079000	2.488360000	0.118043000
C	2.146429000	3.812216000	0.519449000
C	-0.191795000	3.626016000	1.122683000
C	0.959073000	4.380766000	1.022285000
C	2.262236000	-0.279130000	-0.697859000
C	3.425885000	1.903061000	-0.401098000
N	3.377116000	0.563112000	-0.810684000
O	2.334405000	-1.457581000	-1.019643000
O	4.467272000	2.541994000	-0.479076000
N	-2.447662000	-0.724965000	0.483423000
H	-3.707804000	0.714277000	-0.290157000
H	-5.453545000	1.302449000	-0.974237000
H	-7.665890000	2.013290000	-1.690108000
H	-9.379574000	-1.650570000	-0.243097000
H	-9.653916000	0.554540000	-1.335072000
H	-3.746758000	-2.674207000	1.215042000
H	-7.974937000	-3.336597000	0.789418000
H	-5.749678000	-4.126385000	1.540500000
H	-0.090642000	-1.481747000	-0.359661000
H	-2.277358000	1.874100000	1.209990000
H	3.059319000	4.391218000	0.438728000
H	-1.104892000	4.066206000	1.513084000
H	0.954650000	5.420025000	1.334312000
C	4.624393000	-0.033742000	-1.314866000
H	5.173088000	0.759797000	-1.822510000
H	4.340134000	-0.803427000	-2.032669000
C	5.467798000	-0.634345000	-0.190182000
H	5.698984000	0.153606000	0.535938000
H	4.873353000	-1.395839000	0.327785000
C	6.764296000	-1.254580000	-0.714631000
H	7.344080000	-0.489838000	-1.249914000
H	6.524107000	-2.030961000	-1.454224000
C	7.628901000	-1.861312000	0.393275000
H	7.867492000	-1.084858000	1.133475000
H	7.048007000	-2.624220000	0.930112000
C	8.928649000	-2.485067000	-0.122487000
H	9.506646000	-1.721584000	-0.660147000
H	8.688009000	-3.260379000	-0.862098000
C	9.786594000	-3.087696000	0.991792000
H	10.067903000	-2.326287000	1.727905000
H	9.243107000	-3.875792000	1.524948000
H	10.708479000	-3.526751000	0.597409000

AzNI-3

C	-2.513761000	0.670540000	-0.831479000

C	-2.592531000	1.993609000	-0.327307000
C	-1.291120000	0.102902000	-1.116725000
C	-1.392204000	2.735873000	-0.118470000
C	-0.086643000	0.816739000	-0.882666000
C	-0.149557000	2.119939000	-0.403774000
C	-3.845409000	2.584295000	-0.041397000
C	-3.912263000	3.878543000	0.443101000
C	-1.498504000	4.063901000	0.370521000
C	-2.731032000	4.619367000	0.647303000
C	-3.751166000	-0.114196000	-1.063053000
C	-5.097085000	1.820479000	-0.256586000
N	-4.965703000	0.531993000	-0.793325000
O	-3.734666000	-1.268029000	-1.470442000
O	-6.201881000	2.276147000	0.009371000
N	1.113362000	0.176186000	-1.209792000
C	2.130027000	0.308949000	-0.434603000
H	2.032573000	0.857646000	0.508086000
C	3.434990000	-0.260732000	-0.760166000
C	4.545605000	-0.144844000	0.119326000
C	3.584231000	-0.936080000	-1.985364000
C	5.798012000	-0.717716000	-0.273865000
C	4.791679000	-1.491355000	-2.360523000
C	5.918370000	-1.395052000	-1.524293000
C	6.937596000	-0.619026000	0.579361000
C	8.185301000	-1.190089000	0.184383000
C	7.181985000	-1.959464000	-1.895481000
C	8.267423000	-1.861835000	-1.079543000
C	4.486097000	0.518624000	1.393631000
C	5.574812000	0.610760000	2.205629000
C	6.839329000	0.050257000	1.833928000
C	9.291320000	-1.078438000	1.041595000
C	7.970565000	0.137753000	2.659251000
C	9.183531000	-0.421539000	2.264645000
H	-1.247229000	-0.904409000	-1.513537000
H	0.764532000	2.692464000	-0.278990000
H	-4.887560000	4.301416000	0.655466000
H	-0.589730000	4.637766000	0.527888000
H	-2.795575000	5.634877000	1.024185000
H	2.714263000	-1.006933000	-2.627965000
H	4.882728000	-2.008392000	-3.311201000
H	7.254111000	-2.471114000	-2.850718000
H	9.219307000	-2.294389000	-1.373830000
H	3.553541000	0.956064000	1.727285000
H	5.497038000	1.117608000	3.163278000
H	10.238719000	-1.513963000	0.737569000
H	7.888801000	0.648726000	3.614195000
H	10.049660000	-0.345493000	2.914341000
C	-6.201029000	-0.239185000	-1.005754000
H	-6.016269000	-0.908811000	-1.845931000
H	-6.980378000	0.476367000	-1.268556000
C	-6.600612000	-1.035132000	0.236733000
H	-5.779879000	-1.712225000	0.500407000
H	-6.735251000	-0.338996000	1.072710000
C	-7.884981000	-1.836434000	0.015439000

H	-7.747354000	-2.518524000	-0.835006000
H	-8.697168000	-1.152697000	-0.268014000
C	-8.308253000	-2.641700000	1.246472000
H	-7.494954000	-3.323759000	1.531137000
H	-8.444006000	-1.959378000	2.097198000
C	-9.592989000	-3.447149000	1.033939000
H	-9.455383000	-4.127893000	0.183241000
H	-10.403891000	-2.763991000	0.747950000
C	-10.008669000	-4.247839000	2.269632000
H	-9.228167000	-4.960904000	2.557884000
H	-10.183697000	-3.587487000	3.126397000
H	-10.928397000	-4.814136000	2.091885000

AzNI-4

C	2.300901000	0.702448000	-0.410297000
C	2.686806000	2.013782000	-0.041043000
C	0.967403000	0.354289000	-0.470819000
C	1.679594000	2.981614000	0.252491000
C	-0.046815000	1.301029000	-0.164207000
C	0.321634000	2.599015000	0.166640000
C	4.053525000	2.373093000	0.028277000
C	4.418257000	3.657972000	0.387786000
C	2.090941000	4.292212000	0.613013000
C	3.429246000	4.619684000	0.679929000
C	3.329642000	-0.313022000	-0.739550000
C	5.103322000	1.374586000	-0.281275000
N	4.666079000	0.104442000	-0.686056000
O	3.045979000	-1.465001000	-1.043819000
O	6.298954000	1.625305000	-0.200912000
C	-4.216027000	0.325667000	-0.470781000
C	-5.541101000	-0.058208000	-0.554563000
C	-3.225174000	-0.565733000	-0.022895000
C	-5.946219000	-1.370326000	-0.199348000
C	-3.623930000	-1.860444000	0.337083000
C	-4.949705000	-2.260847000	0.258720000
N	-7.269088000	-1.776725000	-0.307494000
C	-7.658007000	-3.081249000	0.206060000
C	-8.322346000	-0.801619000	-0.504985000
C	-8.621479000	0.021317000	0.775839000
C	-9.670697000	1.011655000	0.548489000
N	-10.507820000	1.783320000	0.322531000
H	0.714973000	-0.651776000	-0.785525000
H	-0.461247000	3.321480000	0.373913000
H	5.473460000	3.901740000	0.436328000
H	1.331028000	5.035176000	0.836971000
H	3.728096000	5.624972000	0.958363000
H	-3.917635000	1.330654000	-0.748362000
H	-6.265406000	0.665131000	-0.906726000
H	-2.878722000	-2.568435000	0.690491000
H	-5.208618000	-3.268498000	0.554570000
H	-8.721202000	-3.233341000	0.016572000
H	-7.110999000	-3.878525000	-0.306823000
H	-7.479708000	-3.185020000	1.286041000
H	-8.064640000	-0.125284000	-1.322985000

H	-9.227071000	-1.328270000	-0.815226000
H	-8.931023000	-0.649642000	1.584354000
H	-7.710244000	0.529837000	1.107098000
C	5.698782000	-0.898037000	-0.992653000
H	6.548006000	-0.359969000	-1.414258000
H	5.277620000	-1.568281000	-1.742226000
C	6.123751000	-1.681652000	0.249233000
H	6.502457000	-0.976340000	0.998031000
H	5.240790000	-2.172205000	0.675250000
C	7.195017000	-2.726026000	-0.070382000
H	8.068760000	-2.229216000	-0.514707000
H	6.814359000	-3.417901000	-0.834693000
C	7.639261000	-3.525315000	1.157918000
H	8.017928000	-2.832037000	1.920506000
H	6.764365000	-4.018305000	1.602010000
C	8.709600000	-4.569944000	0.836049000
H	9.606985000	-4.099047000	0.419411000
H	9.009567000	-5.126700000	1.729446000
H	8.344744000	-5.293658000	0.098607000
N	-1.414335000	1.004238000	-0.224045000
C	-1.827480000	-0.175734000	0.074112000
H	-1.142165000	-0.957133000	0.433828000

AzNI-5

C	-4.388811000	0.385012000	-0.776528000
C	-5.701826000	0.818661000	-0.865908000
C	-3.948657000	-0.314901000	0.359782000
C	-4.860191000	-0.564874000	1.395498000
C	-6.623822000	0.568689000	0.169116000
C	-6.177314000	-0.131897000	1.301022000
C	-2.570201000	-0.783030000	0.481093000
C	1.987701000	-0.766953000	-0.555710000
C	2.242251000	-2.082763000	-0.092734000
C	0.695337000	-0.300020000	-0.658399000
C	1.146756000	-2.922971000	0.266640000
C	-0.402277000	-1.113153000	-0.273905000
C	-0.169222000	-2.409453000	0.167759000
C	3.566384000	-2.570320000	0.005427000
C	3.804203000	-3.857982000	0.451990000
C	1.426895000	-4.241018000	0.711930000
C	2.726698000	-4.694971000	0.804718000
C	3.113273000	0.118784000	-0.941974000
C	4.710043000	-1.704636000	-0.367720000
N	4.401495000	-0.427420000	-0.856715000
O	2.946890000	1.270414000	-1.320914000
O	5.874184000	-2.069971000	-0.267074000
N	-1.685749000	-0.572565000	-0.422640000
H	-2.326039000	-1.323629000	1.407972000
C	-8.025237000	1.032422000	0.065873000
C	-8.697886000	1.019648000	-1.166694000
C	-8.715193000	1.495637000	1.197790000
C	-10.017370000	1.454516000	-1.263684000
C	-10.034306000	1.931411000	1.100416000
C	-10.691153000	1.912366000	-0.130678000

H	-3.676542000	0.585673000	-1.569118000
H	-6.021669000	1.383606000	-1.735282000
H	-4.532461000	-1.112121000	2.275495000
H	-6.877201000	-0.359351000	2.098090000
H	0.515486000	0.702988000	-1.026755000
H	-1.007998000	-3.055017000	0.409697000
H	4.830465000	-4.200651000	0.519049000
H	0.598286000	-4.888740000	0.983544000
H	2.924981000	-5.704223000	1.150452000
H	-8.189999000	0.639906000	-2.047400000
H	-8.202381000	1.540411000	2.153255000
H	-10.522849000	1.428182000	-2.224168000
H	-10.547217000	2.295045000	1.985548000
H	-11.719308000	2.252094000	-0.206580000
C	5.528678000	0.443939000	-1.225763000
H	5.174310000	1.101514000	-2.019788000
H	6.318211000	-0.203535000	-1.607694000
C	6.034073000	1.262262000	-0.037548000
H	5.206732000	1.867387000	0.350926000
H	6.341666000	0.574426000	0.758603000
C	7.204818000	2.168705000	-0.422865000
H	6.893423000	2.841540000	-1.233895000
H	8.021953000	1.556439000	-0.828951000
C	7.730365000	2.999894000	0.750152000
H	6.912372000	3.610382000	1.157446000
H	8.040071000	2.326854000	1.561826000
C	8.902109000	3.910615000	0.373172000
H	8.590810000	4.581470000	-0.438660000
H	9.717847000	3.298778000	-0.034608000
C	9.420668000	4.737410000	1.551449000
H	8.632809000	5.381663000	1.957585000
H	9.767914000	4.089959000	2.364447000
H	10.256805000	5.379121000	1.256058000

AzNI-6

C	4.012226000	-1.067948000	-1.413576000
C	5.352025000	-0.713371000	-1.346526000
C	3.122528000	-0.712424000	-0.387921000
C	3.605934000	0.013674000	0.715653000
C	5.838237000	0.013922000	-0.242260000
C	4.940163000	0.372495000	0.788379000
C	1.718417000	-1.101674000	-0.484998000
C	-2.823294000	-0.761185000	0.574394000
C	-3.161203000	-2.075971000	0.164936000
C	-1.504164000	-0.370476000	0.651058000
C	-2.121941000	-2.995054000	-0.167462000
C	-0.461403000	-1.263495000	0.291874000
C	-0.776462000	-2.559512000	-0.096899000
C	-4.513315000	-2.485000000	0.093755000
C	-4.832738000	-3.772138000	-0.300275000
C	-2.485421000	-4.309945000	-0.558656000
C	-3.811164000	-4.686708000	-0.625902000
C	-3.889698000	0.206113000	0.932174000
C	-5.599082000	-1.537025000	0.439007000

N	-5.209400000	-0.263041000	0.875643000
O	-3.650119000	1.359151000	1.264665000
O	-6.784018000	-1.834187000	0.359340000
C	-6.278418000	0.689445000	1.216398000
N	0.853361000	-0.796014000	0.410730000
H	1.437584000	-1.668603000	-1.385175000
C	7.206863000	0.381533000	-0.165264000
C	8.379117000	0.697850000	-0.096917000
C	9.749617000	1.068222000	-0.016529000
C	10.647854000	0.709803000	-1.040581000
C	10.228068000	1.798935000	1.088533000
C	11.987388000	1.074597000	-0.957085000
C	11.569430000	2.159010000	1.162713000
C	12.452633000	1.799096000	0.142495000
C	-6.742979000	1.489814000	-0.000364000
C	-7.852544000	2.481021000	0.356160000
C	-8.337513000	3.295591000	-0.845652000
C	-9.447544000	4.291022000	-0.497111000
C	-9.926493000	5.100142000	-1.704074000
H	3.644150000	-1.627646000	-2.269241000
H	6.037035000	-0.989910000	-2.140165000
H	2.909361000	0.284680000	1.501066000
H	5.313848000	0.933295000	1.638009000
H	-1.260501000	0.633223000	0.978568000
H	0.019277000	-3.264362000	-0.317836000
H	-5.878483000	-4.053735000	-0.347846000
H	-1.700073000	-5.017262000	-0.809305000
H	-4.073445000	-5.694647000	-0.930342000
H	-7.102666000	0.106892000	1.628355000
H	-5.878122000	1.354733000	1.981637000
H	10.279877000	0.147296000	-1.891784000
H	9.536770000	2.075308000	1.877230000
H	12.671402000	0.793477000	-1.751738000
H	11.927956000	2.722246000	2.018614000
H	13.498742000	2.082036000	0.204014000
H	-5.883555000	2.027067000	-0.417881000
H	-7.099146000	0.791372000	-0.766459000
H	-8.701625000	1.936628000	0.792259000
H	-7.493043000	3.165175000	1.137264000
H	-7.487727000	3.837876000	-1.283232000
H	-8.695805000	2.611098000	-1.627190000
H	-10.294946000	3.747309000	-0.058554000
H	-9.087620000	4.973516000	0.284347000
H	-9.104940000	5.678045000	-2.142031000
H	-10.320762000	4.443126000	-2.487411000
H	-10.718910000	5.803286000	-1.428690000

S_1 state in $CHCl_3$

AzNI-1

C	-1.560668000	0.584641000	-0.330040000
C	-1.654530000	1.968896000	-0.005354000
C	-0.344173000	-0.057211000	-0.295561000
C	-0.466863000	2.683856000	0.362813000
C	0.855520000	0.638661000	0.034944000

C	0.764620000	2.007254000	0.382117000
C	-2.897879000	2.629922000	-0.024627000
C	-2.984839000	3.978043000	0.315293000
C	-0.595669000	4.061165000	0.705111000
C	-1.826788000	4.687478000	0.679492000
C	-2.773412000	-0.175172000	-0.696687000
C	-4.122920000	1.894203000	-0.397844000
N	-3.974243000	0.544205000	-0.745185000
O	-2.756586000	-1.376165000	-0.955429000
O	-5.233452000	2.419591000	-0.413317000
N	2.005577000	-0.096940000	0.014498000
C	3.191764000	0.482891000	-0.046405000
C	4.433496000	-0.179826000	0.145554000
C	3.452917000	-2.323245000	1.032921000
C	4.531815000	-1.580708000	0.512385000
C	3.568470000	-3.687851000	1.305031000
C	5.789006000	-2.261263000	0.337726000
C	4.781547000	-4.356119000	1.094419000
C	5.874843000	-3.649691000	0.618775000
C	5.659863000	0.561900000	-0.112324000
C	6.923847000	-1.549858000	-0.085149000
C	6.895193000	-0.157437000	-0.276680000
C	5.707741000	1.966352000	-0.220118000
C	6.886545000	2.640514000	-0.535349000
C	8.077972000	0.551346000	-0.609884000
C	8.076750000	1.929876000	-0.744166000
H	3.243056000	1.528911000	-0.352024000
H	-0.283704000	-1.111151000	-0.540321000
H	1.651709000	2.546630000	0.696665000
H	-3.955394000	4.459272000	0.294071000
H	0.295741000	4.613112000	0.987799000
H	-1.904442000	5.737198000	0.943312000
H	2.518805000	-1.815630000	1.224875000
H	2.710298000	-4.227057000	1.692457000
H	4.868364000	-5.416201000	1.308877000
H	6.824163000	-4.151539000	0.456415000
H	7.863725000	-2.080108000	-0.210104000
H	4.821867000	2.557439000	-0.024360000
H	6.878129000	3.722845000	-0.608962000
H	8.994399000	-0.013407000	-0.752962000
H	8.991868000	2.455749000	-0.995010000
C	-5.191980000	-0.198991000	-1.106379000
H	-4.895778000	-0.969493000	-1.818091000
H	-5.863725000	0.507258000	-1.593863000
C	-5.865508000	-0.825899000	0.114408000
H	-5.150472000	-1.494381000	0.607784000
H	-6.114155000	-0.031079000	0.827226000
C	-7.129140000	-1.601791000	-0.262371000
H	-6.872580000	-2.385384000	-0.988676000
H	-7.831816000	-0.928651000	-0.772746000
C	-7.823527000	-2.237934000	0.944406000
H	-7.119475000	-2.908937000	1.456061000
H	-8.079380000	-1.453898000	1.670730000
C	-9.088425000	-3.018055000	0.575586000

H	-8.830455000	-3.800471000	-0.150589000
H	-9.789709000	-2.346192000	0.062892000
C	-9.776301000	-3.649689000	1.787617000
H	-9.106361000	-4.349442000	2.299893000
H	-10.072721000	-2.885102000	2.514556000
H	-10.676334000	-4.201092000	1.497340000

AzNI-2

C	-6.136089000	-0.585025000	0.105050000
C	-6.490131000	0.772824000	0.244559000
C	-7.187972000	-1.561023000	0.133659000
C	-7.819501000	1.164676000	0.389088000
C	-8.530771000	-1.134625000	0.282490000
C	-8.844810000	0.210247000	0.406936000
C	-4.772540000	-1.037387000	-0.058742000
C	-4.524355000	-2.429898000	-0.168634000
C	-6.877858000	-2.935107000	0.018255000
C	-5.556271000	-3.356885000	-0.128949000
C	-3.664591000	-0.125259000	-0.111437000
C	1.057693000	0.373657000	-0.538125000
C	1.038928000	1.765678000	-0.212411000
C	-0.112844000	-0.365239000	-0.501200000
C	-0.206335000	2.390580000	0.155950000
C	-1.347995000	0.235959000	-0.170951000
C	-1.377040000	1.620017000	0.173259000
C	2.224246000	2.523299000	-0.234242000
C	2.203007000	3.886442000	0.106612000
C	-0.186098000	3.776758000	0.493460000
C	0.997774000	4.498656000	0.467022000
C	2.315430000	-0.283199000	-0.905482000
C	3.495779000	1.891868000	-0.606490000
N	3.454670000	0.532717000	-0.950544000
O	2.398446000	-1.486564000	-1.171814000
O	4.566718000	2.505531000	-0.628592000
N	-2.438611000	-0.592671000	-0.212366000
H	-3.868386000	0.943713000	-0.099036000
H	-5.725512000	1.539136000	0.249541000
H	-8.056236000	2.218065000	0.491047000
H	-9.315911000	-1.883981000	0.297448000
H	-9.877575000	0.520775000	0.519997000
H	-3.495675000	-2.742833000	-0.295773000
H	-7.685209000	-3.660203000	0.043611000
H	-5.334634000	-4.414583000	-0.220906000
H	-0.091926000	-1.419654000	-0.748347000
H	-2.303063000	2.091909000	0.480899000
H	3.134390000	4.439191000	0.083223000
H	-1.118291000	4.257891000	0.773825000
H	0.990447000	5.552186000	0.729171000
C	4.725648000	-0.106492000	-1.318505000
H	5.333180000	0.651652000	-1.812601000
H	4.489850000	-0.903534000	-2.023666000
C	5.460569000	-0.669887000	-0.101803000
H	5.641012000	0.144832000	0.609062000
H	4.810800000	-1.399227000	0.395768000

C	6.786863000	-1.329573000	-0.484325000
H	7.423773000	-0.596262000	-0.998308000
H	6.598751000	-2.133580000	-1.209427000
C	7.542006000	-1.899846000	0.718869000
H	7.727850000	-1.095793000	1.444651000
H	6.904564000	-2.632590000	1.233243000
C	8.871754000	-2.560619000	0.344999000
H	9.506603000	-1.827055000	-0.169752000
H	8.684106000	-3.363137000	-0.380870000
C	9.619301000	-3.126963000	1.554007000
H	9.846519000	-2.338667000	2.280562000
H	9.018378000	-3.885613000	2.068096000
H	10.565347000	-3.593056000	1.260353000

AzNI-3

C	-2.515426000	0.589860000	-0.407497000
C	-2.611601000	1.880403000	0.193607000
C	-1.274552000	0.000668000	-0.592897000
C	-1.409252000	2.560033000	0.598336000
C	-0.085265000	0.644877000	-0.189652000
C	-0.167219000	1.933853000	0.399255000
C	-3.866002000	2.491068000	0.391381000
C	-3.952710000	3.760370000	0.981229000
C	-1.539714000	3.851228000	1.187327000
C	-2.786507000	4.429428000	1.370948000
C	-3.724793000	-0.121586000	-0.830012000
C	-5.093848000	1.797316000	-0.016765000
N	-4.942204000	0.548020000	-0.636410000
O	-3.709309000	-1.248756000	-1.334926000
O	-6.221370000	2.267799000	0.159495000
N	1.072675000	-0.067549000	-0.418349000
C	2.237454000	0.352563000	0.006199000
H	2.340954000	1.262406000	0.594846000
C	3.425206000	-0.412285000	-0.284716000
C	4.737849000	0.009988000	0.148104000
C	3.306133000	-1.620106000	-1.018848000
C	5.868766000	-0.802886000	-0.164742000
C	4.401739000	-2.393451000	-1.316113000
C	5.709289000	-2.014923000	-0.901305000
C	7.170712000	-0.413136000	0.253871000
C	8.300713000	-1.227396000	-0.057654000
C	6.844403000	-2.807101000	-1.200430000
C	8.105556000	-2.429580000	-0.792508000
C	4.957988000	1.209529000	0.876209000
C	6.220386000	1.589356000	1.283341000
C	7.356378000	0.794188000	0.987970000
C	9.582817000	-0.815539000	0.372896000
C	8.661573000	1.165660000	1.397793000
C	9.756387000	0.366846000	1.091598000
H	-1.208602000	-0.980238000	-1.048134000
H	0.729006000	2.469614000	0.690724000
H	-4.932035000	4.201916000	1.120753000
H	-0.640037000	4.376757000	1.493594000
H	-2.860479000	5.413772000	1.823256000

H	2.312875000	-1.913145000	-1.334126000
H	4.280710000	-3.315467000	-1.876387000
H	6.705207000	-3.725679000	-1.761594000
H	8.966351000	-3.046775000	-1.028488000
H	4.125113000	1.854639000	1.122756000
H	6.353812000	2.511983000	1.838807000
H	10.439615000	-1.437749000	0.134002000
H	8.793260000	2.086899000	1.956354000
H	10.749355000	0.662991000	1.411297000
C	-6.166834000	-0.152448000	-1.046606000
H	-5.913214000	-0.756203000	-1.918028000
H	-6.892790000	0.610250000	-1.328420000
C	-6.726030000	-1.035060000	0.069556000
H	-5.959471000	-1.762946000	0.359831000
H	-6.928990000	-0.407849000	0.945443000
C	-8.001770000	-1.764181000	-0.356226000
H	-7.792297000	-2.377022000	-1.244042000
H	-8.757293000	-1.027839000	-0.663794000
C	-8.581863000	-2.653274000	0.746553000
H	-7.825312000	-3.388052000	1.055708000
H	-8.790986000	-2.040138000	1.634320000
C	-9.858339000	-3.387770000	0.327296000
H	-9.647327000	-3.999588000	-0.560006000
H	-10.612317000	-2.651926000	0.017163000
C	-10.431351000	-4.272864000	1.436149000
H	-9.708179000	-5.037036000	1.742793000
H	-10.680346000	-3.679975000	2.323512000
H	-11.342088000	-4.786301000	1.111411000

AzNI-4

C	2.328440000	0.572652000	-0.420177000
C	2.683042000	1.913038000	-0.091837000
C	0.982818000	0.175127000	-0.448634000
C	1.650585000	2.876205000	0.188798000
C	-0.019561000	1.114532000	-0.169690000
C	0.304806000	2.450123000	0.116037000
C	4.042231000	2.312850000	-0.045394000
C	4.373020000	3.646160000	0.284781000
C	2.024758000	4.204584000	0.510816000
C	3.371607000	4.572038000	0.555099000
C	3.363864000	-0.403105000	-0.722331000
C	5.096424000	1.355179000	-0.330090000
N	4.686708000	0.057650000	-0.684504000
O	3.120484000	-1.593034000	-1.002318000
O	6.309441000	1.630164000	-0.279178000
C	-4.194468000	0.343431000	-0.248862000
C	-5.533930000	0.058924000	-0.294593000
C	-3.233197000	-0.681860000	-0.053453000
C	-5.996532000	-1.289256000	-0.147341000
C	-3.685844000	-2.013797000	0.093005000
C	-5.024647000	-2.319712000	0.050500000
N	-7.316800000	-1.595608000	-0.187293000
C	-7.785688000	-2.960584000	0.074395000
C	-8.343458000	-0.582775000	-0.425356000

C	-8.792195000	0.072845000	0.901542000
C	-9.840124000	1.064252000	0.668925000
N	-10.670368000	1.846691000	0.458467000
H	0.760605000	-0.846191000	-0.728497000
H	-0.501621000	3.149443000	0.309802000
H	5.421043000	3.919317000	0.316441000
H	1.246921000	4.931980000	0.722001000
H	3.639217000	5.594743000	0.804649000
H	-3.840437000	1.361128000	-0.355617000
H	-6.234859000	0.870160000	-0.431663000
H	-2.960539000	-2.806974000	0.238194000
H	-5.336088000	-3.348707000	0.157619000
H	-8.872662000	-2.955783000	0.109139000
H	-7.464077000	-3.635642000	-0.723375000
H	-7.405738000	-3.323104000	1.031653000
H	-7.972239000	0.167245000	-1.121541000
H	-9.190765000	-1.073063000	-0.904258000
H	-9.172007000	-0.688579000	1.589992000
H	-7.942799000	0.560398000	1.390090000
C	5.741791000	-0.918083000	-0.975717000
H	6.571070000	-0.370417000	-1.423917000
H	5.332982000	-1.626758000	-1.696348000
C	6.209957000	-1.650756000	0.282230000
H	6.572912000	-0.909040000	1.003152000
H	5.347615000	-2.153358000	0.736084000
C	7.310684000	-2.670700000	-0.013771000
H	8.164099000	-2.161424000	-0.483265000
H	6.945966000	-3.399071000	-0.751899000
C	7.792816000	-3.415727000	1.234244000
H	8.155168000	-2.686188000	1.970619000
H	6.938702000	-3.922669000	1.702804000
C	8.894479000	-4.434802000	0.936126000
H	9.771947000	-3.947847000	0.495822000
H	9.220666000	-4.952607000	1.843975000
H	8.547212000	-5.193256000	0.225297000
N	-1.394306000	0.820948000	-0.156715000
C	-1.813221000	-0.389059000	-0.007771000
H	-1.142983000	-1.237079000	0.162414000

AzNI-5

C	3.977208000	-0.896304000	0.458161000
C	5.154208000	-0.697921000	-0.234113000
C	3.880470000	-0.575343000	1.847539000
C	5.052863000	-0.057085000	2.479234000
C	6.307747000	-0.176065000	0.392469000
C	6.218479000	0.134466000	1.770257000
C	2.692009000	-0.730935000	2.569731000
C	-1.566927000	-0.544322000	0.358071000
C	-1.462860000	0.868812000	0.152539000
C	-0.572085000	-1.232188000	0.986970000
C	-0.299892000	1.566432000	0.612522000
C	0.619045000	-0.560580000	1.468366000
C	0.717705000	0.850868000	1.259701000
C	-2.490480000	1.573165000	-0.493758000

C	-2.385412000	2.947455000	-0.688699000
C	-0.223224000	2.970646000	0.392757000
C	-1.246984000	3.642958000	-0.244258000
C	-2.772104000	-1.275051000	-0.115420000
C	-3.700444000	0.863490000	-0.972678000
N	-3.743759000	-0.523304000	-0.783173000
O	-2.921464000	-2.477652000	0.059341000
O	-4.630404000	1.447309000	-1.516365000
N	1.538361000	-1.310989000	2.055940000
H	2.675590000	-0.494026000	3.632758000
C	7.558340000	0.033157000	-0.359098000
C	7.536437000	0.367004000	-1.726293000
C	8.812364000	-0.095197000	0.267091000
C	8.718022000	0.562263000	-2.436955000
C	9.993498000	0.102627000	-0.443664000
C	9.953888000	0.432057000	-1.800082000
H	3.114663000	-1.317513000	-0.045448000
H	5.202778000	-0.979608000	-1.281052000
H	5.009292000	0.202193000	3.533013000
H	7.079854000	0.559691000	2.274904000
H	-0.655831000	-2.301226000	1.139821000
H	1.605942000	1.368410000	1.601750000
H	-3.196341000	3.463319000	-1.188900000
H	0.656621000	3.504312000	0.737191000
H	-1.177162000	4.713010000	-0.404759000
H	6.583694000	0.501171000	-2.227975000
H	8.860488000	-0.381111000	1.312699000
H	8.673407000	0.826716000	-3.489167000
H	10.948257000	-0.011047000	0.060908000
H	10.874684000	0.585127000	-2.353983000
C	-4.949039000	-1.232935000	-1.247358000
H	-4.638309000	-2.241033000	-1.520573000
H	-5.303396000	-0.709810000	-2.135203000
C	-6.037799000	-1.274725000	-0.175180000
H	-5.637132000	-1.767578000	0.718176000
H	-6.295712000	-0.247446000	0.107279000
C	-7.288980000	-2.010970000	-0.658494000
H	-7.018683000	-3.034050000	-0.953949000
H	-7.673370000	-1.521088000	-1.563694000
C	-8.395651000	-2.064091000	0.397755000
H	-8.009774000	-2.552680000	1.303283000
H	-8.663474000	-1.040398000	0.694411000
C	-9.652680000	-2.798422000	-0.076777000
H	-9.382659000	-3.820685000	-0.373446000
H	-10.035524000	-2.309484000	-0.982521000
C	-10.753414000	-2.846041000	0.984930000
H	-10.405905000	-3.358067000	1.889306000
H	-11.063145000	-1.836038000	1.276190000
H	-11.639977000	-3.375310000	0.621441000

AzNI-6

C	4.275762000	-1.897982000	-0.887036000
C	5.563635000	-1.428064000	-0.798486000
C	3.179658000	-1.131542000	-0.397809000

C	3.446870000	0.142286000	0.189055000
C	5.827092000	-0.154020000	-0.210165000
C	4.730593000	0.617235000	0.280543000
C	1.847291000	-1.641967000	-0.494682000
C	-2.787587000	-0.839698000	0.355490000
C	-3.199787000	-2.139230000	-0.071357000
C	-1.449635000	-0.499714000	0.349679000
C	-2.205526000	-3.086657000	-0.501778000
C	-0.452421000	-1.412601000	-0.078349000
C	-0.853933000	-2.714102000	-0.496032000
C	-4.559995000	-2.496179000	-0.073944000
C	-4.958577000	-3.772082000	-0.494650000
C	-2.651001000	-4.377307000	-0.917430000
C	-3.996774000	-4.702840000	-0.911006000
C	-3.783729000	0.144968000	0.800332000
C	-5.577787000	-1.527360000	0.362779000
N	-5.120248000	-0.278046000	0.805713000
O	-3.490963000	1.287627000	1.158102000
O	-6.784165000	-1.777583000	0.354228000
C	-6.133361000	0.696276000	1.236731000
N	0.829586000	-0.936035000	-0.042880000
H	1.730741000	-2.621093000	-0.965727000
C	7.131332000	0.328573000	-0.120292000
C	8.281033000	0.756801000	-0.040430000
C	9.589818000	1.248381000	0.052311000
C	10.680869000	0.484569000	-0.438287000
C	9.839190000	2.517065000	0.637952000
C	11.971794000	0.979580000	-0.342627000
C	11.136392000	2.996909000	0.725490000
C	12.205113000	2.233476000	0.237464000
C	-6.643494000	1.546921000	0.073200000
C	-7.694721000	2.564404000	0.520590000
C	-8.220312000	3.427571000	-0.629179000
C	-9.273267000	4.449124000	-0.190688000
C	-9.792596000	5.306618000	-1.346667000
H	4.081176000	-2.867488000	-1.335153000
H	6.395539000	-2.014124000	-1.172420000
H	2.606413000	0.718240000	0.556176000
H	4.932092000	1.585499000	0.725142000
H	-1.144179000	0.488508000	0.672091000
H	-0.117604000	-3.445700000	-0.808031000
H	-6.014176000	-4.015492000	-0.486525000
H	-1.911324000	-5.102944000	-1.242189000
H	-4.312865000	-5.690364000	-1.232446000
H	-6.950979000	0.130725000	1.683535000
H	-5.667735000	1.326384000	1.994464000
H	10.487364000	-0.484019000	-0.885138000
H	9.003720000	3.098113000	1.011659000
H	12.803482000	0.393324000	-0.718600000
H	11.323442000	3.966944000	1.173565000
H	13.218249000	2.615121000	0.309047000
H	-5.792576000	2.065990000	-0.383049000
H	-7.067702000	0.883174000	-0.689126000
H	-8.535283000	2.036900000	0.992475000

H	-7.266320000	3.214242000	1.296280000
H	-7.379049000	3.953611000	-1.101776000
H	-8.647028000	2.777055000	-1.405393000
H	-10.112360000	3.921550000	0.282254000
H	-8.845236000	5.097629000	0.585417000
H	-8.977601000	5.868521000	-1.816764000
H	-10.253703000	4.684255000	-2.121883000
H	-10.543062000	6.027850000	-1.007434000

T_1 state

AzNI-1

C	-1.564685000	0.502986000	-0.611952000
C	-1.671326000	1.916927000	-0.509777000
C	-0.333245000	-0.105722000	-0.563236000
C	-0.490256000	2.702272000	-0.353264000
C	0.858802000	0.655716000	-0.378015000
C	0.762907000	2.050504000	-0.285479000
C	-2.932005000	2.549538000	-0.570150000
C	-3.024519000	3.929670000	-0.475232000
C	-0.620656000	4.113292000	-0.266824000
C	-1.863455000	4.711822000	-0.324497000
C	-2.783340000	-0.324351000	-0.779626000
C	-4.162966000	1.743680000	-0.734181000
N	-4.002630000	0.358198000	-0.864138000
O	-2.746308000	-1.547664000	-0.845578000
O	-5.281175000	2.246203000	-0.762459000
N	2.035666000	-0.064108000	-0.357738000
C	3.145063000	0.480422000	0.172778000
C	4.391041000	-0.114407000	0.059998000
C	3.940570000	-1.849601000	-1.729504000
C	4.630511000	-1.425054000	-0.600708000
C	4.145424000	-3.141521000	-2.255754000
C	5.601824000	-2.273385000	-0.002624000
C	5.048142000	-3.994126000	-1.641662000
C	5.778132000	-3.559047000	-0.521675000
C	5.559407000	0.521075000	0.731718000
C	6.417766000	-1.770487000	1.089821000
C	6.518810000	-0.341644000	1.324375000
C	5.701537000	1.897708000	0.839407000
C	6.760735000	2.458876000	1.583306000
C	7.550790000	0.225276000	2.077926000
C	7.666514000	1.620351000	2.212670000
H	3.087072000	1.406748000	0.747377000
H	-0.255682000	-1.182562000	-0.653374000
H	1.657443000	2.656208000	-0.192554000
H	-4.004833000	4.389485000	-0.522030000
H	0.274702000	4.716012000	-0.150488000
H	-1.950721000	5.790501000	-0.254320000
H	3.226099000	-1.184918000	-2.196716000
H	3.583315000	-3.462842000	-3.126315000
H	5.203564000	-4.997915000	-2.025452000
H	6.496431000	-4.224746000	-0.051548000

H	7.144063000	-2.432243000	1.550708000
H	4.989856000	2.552441000	0.346057000
H	6.851158000	3.536729000	1.666103000
H	8.272140000	-0.426763000	2.562063000
H	8.477303000	2.035281000	2.803664000
C	-5.223241000	-0.452121000	-1.017186000
H	-4.950254000	-1.331060000	-1.600751000
H	-5.936191000	0.150073000	-1.580017000
C	-5.811904000	-0.862148000	0.332730000
H	-5.057720000	-1.429656000	0.890200000
H	-6.032927000	0.042198000	0.911417000
C	-7.081880000	-1.700455000	0.173404000
H	-6.855258000	-2.597162000	-0.419637000
H	-7.824502000	-1.131018000	-0.402342000
C	-7.692690000	-2.119671000	1.512953000
H	-6.949386000	-2.688750000	2.088507000
H	-7.915550000	-1.222107000	2.106596000
C	-8.966253000	-2.956556000	1.364080000
H	-8.741552000	-3.852691000	0.770420000
H	-9.706650000	-2.386672000	0.786946000
C	-9.570459000	-3.369442000	2.707808000
H	-8.861071000	-3.967200000	3.291152000
H	-9.832834000	-2.490805000	3.307841000
H	-10.478666000	-3.965741000	2.574109000

AzNI-2

C	-6.113948000	-0.715579000	0.004115000
C	-6.483607000	0.619644000	0.248442000
C	-7.142703000	-1.707670000	-0.015645000
C	-7.809279000	0.979849000	0.465375000
C	-8.481594000	-1.319353000	0.207222000
C	-8.815478000	0.005143000	0.444759000
C	-4.723263000	-1.128909000	-0.230141000
C	-4.467482000	-2.526602000	-0.468411000
C	-6.813918000	-3.079805000	-0.259098000
C	-5.490595000	-3.460857000	-0.479378000
C	-3.667671000	-0.222498000	-0.226544000
C	1.091364000	0.313782000	-0.716243000
C	1.046910000	1.726373000	-0.451312000
C	-0.052600000	-0.432748000	-0.702329000
C	-0.207971000	2.351804000	-0.169743000
C	-1.338833000	0.155674000	-0.432293000
C	-1.383582000	1.571042000	-0.163241000
C	2.221324000	2.487708000	-0.462806000
C	2.177658000	3.862195000	-0.196097000
C	-0.220355000	3.745797000	0.094006000
C	0.955208000	4.483845000	0.080245000
C	2.386806000	-0.344942000	-1.003953000
C	3.524954000	1.849727000	-0.753556000
N	3.516963000	0.479263000	-1.041180000
O	2.485687000	-1.550179000	-1.207648000
O	4.577686000	2.479381000	-0.751579000
N	-2.376607000	-0.682130000	-0.457078000
H	-3.857054000	0.830659000	-0.052790000

H	-5.728870000	1.397023000	0.271821000
H	-8.060243000	2.019212000	0.650883000
H	-9.253557000	-2.083291000	0.189309000
H	-9.850454000	0.284843000	0.613942000
H	-3.440952000	-2.821477000	-0.640546000
H	-7.612624000	-3.814457000	-0.269019000
H	-5.256653000	-4.505179000	-0.663215000
H	-0.010930000	-1.496953000	-0.900948000
H	-2.323825000	2.061176000	0.053484000
H	3.104937000	4.422253000	-0.210329000
H	-1.168442000	4.229122000	0.308733000
H	0.927381000	5.548583000	0.284969000
C	4.812787000	-0.161619000	-1.323830000
H	5.433973000	0.583296000	-1.820548000
H	4.616603000	-0.985795000	-2.009413000
C	5.492902000	-0.669160000	-0.052347000
H	5.637816000	0.174248000	0.632756000
H	4.826061000	-1.382672000	0.445317000
C	6.838028000	-1.334867000	-0.350065000
H	7.493797000	-0.618291000	-0.863589000
H	6.685323000	-2.167728000	-1.050195000
C	7.540097000	-1.852844000	0.907676000
H	7.691105000	-1.019674000	1.608186000
H	6.882680000	-2.567790000	1.421757000
C	8.887012000	-2.521455000	0.618890000
H	9.541820000	-1.805680000	0.104184000
H	8.733811000	-3.352678000	-0.082241000
C	9.581552000	-3.036412000	1.881244000
H	9.774240000	-2.219248000	2.585505000
H	8.960641000	-3.777169000	2.397572000
H	10.540871000	-3.509255000	1.647968000

AzNI-3

C	-2.506263000	0.536020000	-0.293383000
C	-2.594376000	1.815591000	0.335542000
C	-1.288228000	-0.071351000	-0.462656000
C	-1.400027000	2.458822000	0.786210000
C	-0.070970000	0.547280000	-0.031247000
C	-0.156405000	1.818751000	0.596061000
C	-3.840607000	2.439822000	0.518919000
C	-3.919626000	3.684896000	1.140457000
C	-1.515732000	3.727792000	1.413261000
C	-2.752401000	4.325449000	1.584944000
C	-3.733959000	-0.144947000	-0.762963000
C	-5.080728000	1.777923000	0.057361000
N	-4.941268000	0.542812000	-0.588568000
O	-3.719247000	-1.254515000	-1.285876000
O	-6.191555000	2.273410000	0.219207000
N	1.059756000	-0.167005000	-0.269326000
C	2.279229000	0.320358000	0.053395000
H	2.387309000	1.309959000	0.490241000
C	3.427136000	-0.443174000	-0.182596000
C	4.778853000	0.039461000	0.143307000
C	3.301368000	-1.754997000	-0.768180000

C	5.901814000	-0.795431000	-0.137556000
C	4.386197000	-2.532251000	-1.024335000
C	5.723708000	-2.089965000	-0.724415000
C	7.220861000	-0.348732000	0.164608000
C	8.349243000	-1.184810000	-0.118065000
C	6.842475000	-2.891603000	-0.991975000
C	8.130104000	-2.457611000	-0.699399000
C	5.016068000	1.299539000	0.724573000
C	6.298028000	1.740749000	1.021845000
C	7.426686000	0.936739000	0.751610000
C	9.649616000	-0.712559000	0.192757000
C	8.752734000	1.365028000	1.044903000
C	9.838648000	0.546092000	0.765806000
H	-1.228248000	-1.043441000	-0.937215000
H	0.734690000	2.323226000	0.949812000
H	-4.894338000	4.140314000	1.269513000
H	-0.613932000	4.224381000	1.758107000
H	-2.822979000	5.294884000	2.066354000
H	2.300468000	-2.096459000	-0.998641000
H	4.260256000	-3.515993000	-1.467698000
H	6.689814000	-3.870440000	-1.437379000
H	8.983575000	-3.092952000	-0.914413000
H	4.185705000	1.956345000	0.954064000
H	6.441817000	2.719152000	1.469808000
H	10.502305000	-1.348955000	-0.023156000
H	8.899428000	2.343974000	1.490583000
H	10.843688000	0.886099000	0.994714000
C	-6.171384000	-0.125385000	-1.045531000
H	-5.907406000	-0.709339000	-1.927081000
H	-6.873717000	0.658912000	-1.326905000
C	-6.772535000	-1.024541000	0.034762000
H	-6.027204000	-1.773375000	0.326667000
H	-6.988806000	-0.416848000	0.921121000
C	-8.048707000	-1.719883000	-0.443730000
H	-7.825497000	-2.314067000	-1.340583000
H	-8.782949000	-0.962809000	-0.751952000
C	-8.671799000	-2.625476000	0.621537000
H	-7.935953000	-3.380568000	0.931393000
H	-8.894097000	-2.030712000	1.518496000
C	-9.948732000	-3.327005000	0.150473000
H	-9.724360000	-3.920183000	-0.746076000
H	-10.681849000	-2.570740000	-0.160401000
C	-10.565110000	-4.229210000	1.221564000
H	-9.863165000	-5.013246000	1.527222000
H	-10.827544000	-3.654347000	2.116851000
H	-11.475318000	-4.718389000	0.860257000

AzNI-4

C	2.312991000	0.540247000	-0.432228000
C	2.675249000	1.886782000	-0.202230000
C	0.970969000	0.143062000	-0.433168000
C	1.652725000	2.867849000	0.032288000
C	-0.051806000	1.103681000	-0.200641000
C	0.312351000	2.449085000	0.022966000

C	4.033622000	2.291763000	-0.193286000
C	4.360678000	3.633051000	0.043861000
C	2.026320000	4.217257000	0.264335000
C	3.365247000	4.590161000	0.268989000
C	3.360126000	-0.458982000	-0.666220000
C	5.097914000	1.313391000	-0.423982000
N	4.687515000	-0.001040000	-0.677738000
O	3.114109000	-1.656035000	-0.851527000
O	6.299669000	1.605339000	-0.404407000
C	-4.220017000	0.396723000	-0.163911000
C	-5.563516000	0.126079000	-0.172231000
C	-3.246272000	-0.644823000	-0.320384000
C	-6.049948000	-1.207163000	-0.339363000
C	-3.740653000	-1.977948000	-0.480652000
C	-5.083610000	-2.251609000	-0.488701000
N	-7.391408000	-1.492520000	-0.362384000
C	-7.852886000	-2.878009000	-0.417517000
C	-8.390069000	-0.464129000	-0.123014000
C	-8.583768000	-0.196350000	1.391267000
C	-9.584078000	0.841588000	1.627349000
N	-10.383429000	1.669409000	1.779278000
H	0.754803000	-0.898880000	-0.619217000
H	-0.487216000	3.162208000	0.195213000
H	5.409802000	3.906751000	0.046619000
H	1.247279000	4.952852000	0.439399000
H	3.641448000	5.623487000	0.448078000
H	-3.863184000	1.410862000	-0.034782000
H	-6.255214000	0.948662000	-0.045653000
H	-3.028984000	-2.789486000	-0.598174000
H	-5.408807000	-3.275799000	-0.612186000
H	-8.941540000	-2.886590000	-0.403361000
H	-7.519653000	-3.363682000	-1.339389000
H	-7.489432000	-3.458679000	0.437144000
H	-8.111058000	0.457975000	-0.634346000
H	-9.333376000	-0.793868000	-0.559708000
H	-8.900206000	-1.114423000	1.896489000
H	-7.635746000	0.114665000	1.841211000
C	5.740565000	-1.001632000	-0.901445000
H	6.568434000	-0.489617000	-1.392027000
H	5.327934000	-1.756340000	-1.570942000
C	6.208431000	-1.643553000	0.405033000
H	6.572180000	-0.854645000	1.073840000
H	5.347541000	-2.114625000	0.893751000
C	7.309313000	-2.680905000	0.176729000
H	8.161469000	-2.204472000	-0.327850000
H	6.943014000	-3.457898000	-0.508804000
C	7.793045000	-3.338182000	1.472523000
H	8.156514000	-2.559896000	2.156520000
H	6.939817000	-3.812151000	1.975619000
C	8.894371000	-4.374901000	1.242056000
H	9.771013000	-3.919013000	0.768221000
H	9.221801000	-4.829348000	2.182619000
H	8.545798000	-5.180015000	0.585401000
N	-1.408527000	0.876721000	-0.165857000

C	-1.863365000	-0.382964000	-0.320412000
H	-1.193358000	-1.234286000	-0.439754000

AzNI-5

C	-4.207828000	0.643579000	-0.227896000
C	-5.464694000	1.200933000	-0.329918000
C	-4.031087000	-0.666058000	0.312102000
C	-5.198724000	-1.364612000	0.738958000
C	-6.622432000	0.506199000	0.096382000
C	-6.448518000	-0.793000000	0.634481000
C	-2.758692000	-1.267263000	0.428663000
C	1.957309000	-0.792400000	-0.309168000
C	2.261306000	-2.107651000	0.202936000
C	0.666381000	-0.338247000	-0.349050000
C	1.198706000	-2.946008000	0.672031000
C	-0.430311000	-1.140443000	0.106975000
C	-0.127337000	-2.471624000	0.623795000
C	3.579001000	-2.560885000	0.245736000
C	3.879751000	-3.844623000	0.750173000
C	1.529795000	-4.232017000	1.170341000
C	2.853906000	-4.668266000	1.205738000
C	3.047569000	0.080822000	-0.794095000
C	4.683368000	-1.702138000	-0.232233000
N	4.342015000	-0.449292000	-0.754850000
O	2.849916000	1.216089000	-1.217843000
O	5.857230000	-2.058827000	-0.184403000
N	-1.638960000	-0.591634000	0.017111000
H	-2.708607000	-2.269069000	0.852813000
C	-7.960448000	1.111045000	-0.015103000
C	-8.260790000	2.021472000	-1.046437000
C	-8.977821000	0.796294000	0.906112000
C	-9.526098000	2.592163000	-1.151971000
C	-10.242245000	1.368928000	0.800184000
C	-10.523886000	2.269654000	-0.229392000
H	-3.328360000	1.190638000	-0.545067000
H	-5.565589000	2.208896000	-0.718419000
H	-5.093078000	-2.367753000	1.142165000
H	-7.319633000	-1.361531000	0.942038000
H	0.450609000	0.651446000	-0.733146000
H	-0.920583000	-3.115918000	0.979110000
H	4.914950000	-4.162403000	0.769808000
H	0.731907000	-4.876044000	1.526688000
H	3.084843000	-5.655474000	1.591400000
H	-7.504861000	2.259501000	-1.787246000
H	-8.765680000	0.120987000	1.728279000
H	-9.736716000	3.283997000	-1.961705000
H	-11.007448000	1.118380000	1.528463000
H	-11.510089000	2.715349000	-0.311893000
C	5.440133000	0.411767000	-1.225513000
H	5.039800000	1.023165000	-2.034062000
H	6.215083000	-0.246403000	-1.617655000
C	5.997144000	1.294669000	-0.108701000
H	5.185241000	1.912234000	0.292424000
H	6.351983000	0.653189000	0.706383000

C	7.136700000	2.189255000	-0.600879000
H	6.777008000	2.814684000	-1.429518000
H	7.939204000	1.563162000	-1.014712000
C	7.710180000	3.086625000	0.498687000
H	6.906567000	3.711306000	0.912970000
H	8.067948000	2.460835000	1.328192000
C	8.851208000	3.985670000	0.014561000
H	8.491900000	4.608633000	-0.815383000
H	9.652738000	3.359490000	-0.399549000
C	9.417611000	4.879899000	1.119396000
H	8.643019000	5.538325000	1.528463000
H	9.811764000	4.280285000	1.947635000
H	10.230807000	5.511675000	0.748087000

AzNI-6

C	4.304800000	-1.991759000	-0.658721000
C	5.586044000	-1.507706000	-0.597497000
C	3.183964000	-1.183139000	-0.272665000
C	3.452044000	0.153419000	0.181592000
C	5.843816000	-0.177161000	-0.145684000
C	4.735332000	0.633835000	0.240722000
C	1.882806000	-1.698033000	-0.345842000
C	-2.790820000	-0.825626000	0.344247000
C	-3.195136000	-2.124024000	-0.128766000
C	-1.469496000	-0.474810000	0.373957000
C	-2.201447000	-3.054226000	-0.571108000
C	-0.437216000	-1.377802000	-0.054755000
C	-0.841629000	-2.683388000	-0.531629000
C	-4.547163000	-2.476149000	-0.162446000
C	-4.942412000	-3.740091000	-0.631148000
C	-2.630767000	-4.324106000	-1.036323000
C	-3.980703000	-4.655685000	-1.063838000
C	-3.810782000	0.145922000	0.800087000
C	-5.581674000	-1.520824000	0.291508000
N	-5.142300000	-0.283517000	0.777513000
O	-3.524692000	1.274733000	1.186239000
O	-6.779265000	-1.785594000	0.254805000
C	-6.170152000	0.674075000	1.220128000
N	0.812271000	-0.918190000	0.027464000
H	1.758026000	-2.720501000	-0.699085000
C	7.154719000	0.317778000	-0.084068000
C	8.298335000	0.748955000	-0.031029000
C	9.621965000	1.249483000	0.030131000
C	10.713617000	0.436712000	-0.345724000
C	9.869674000	2.568947000	0.467500000
C	12.010513000	0.933845000	-0.284043000
C	11.171078000	3.054747000	0.524659000
C	12.245046000	2.241844000	0.150211000
C	-6.667370000	1.552781000	0.072324000
C	-7.734265000	2.547950000	0.533142000
C	-8.249120000	3.439246000	-0.599905000
C	-9.318133000	4.438183000	-0.148374000
C	-9.827251000	5.324162000	-1.287229000
H	4.122779000	-3.005113000	-1.004579000

H	6.424448000	-2.129599000	-0.892273000
H	2.611335000	0.769435000	0.475659000
H	4.926812000	1.644650000	0.585103000
H	-1.174817000	0.505400000	0.728907000
H	-0.102410000	-3.397804000	-0.869950000
H	-5.998732000	-3.979728000	-0.645976000
H	-1.884841000	-5.037108000	-1.373371000
H	-4.289340000	-5.631227000	-1.423773000
H	-6.989109000	0.090460000	1.639995000
H	-5.719925000	1.283565000	2.003363000
H	10.525985000	-0.577256000	-0.681960000
H	9.032815000	3.195304000	0.756805000
H	12.842619000	0.300673000	-0.575244000
H	11.350490000	4.070624000	0.862139000
H	13.259153000	2.625551000	0.196552000
H	-5.814278000	2.092112000	-0.355300000
H	-7.074214000	0.908590000	-0.715809000
H	-8.577146000	1.999720000	0.976103000
H	-7.323302000	3.178426000	1.333616000
H	-7.405363000	3.986112000	-1.043351000
H	-8.657716000	2.807778000	-1.401146000
H	-10.159447000	3.889609000	0.295784000
H	-8.908070000	5.067548000	0.652708000
H	-9.010371000	5.906821000	-1.727763000
H	-10.270494000	4.720585000	-2.087297000
H	-10.589539000	6.028373000	-0.938948000

8. Electrochemical investigations in dry acetonitrile

Table S7. The electrochemical data investigated in acetonitrile.

Molecule	Method	E_{red}^{1} (V)	$E_{red}^{1(onset)}$ (V)	E_{ox}^{1} (V)	$E_{ox}^{1(onset)}$ (V)	E_{LUMO} (eV)	E_{HOMO} (eV)	E_g (eV)
**AzNI–1**	CV	−1.61	−1.45	0.82	0.66	−3.65	−5.76	2.12
	DPV	−1.53	−1.19	0.73	0.62	−3.91	−5.72	1.81
**AzNI–2**	CV	−1.77	−1.60	0.87	0.66	−3.50	−5.76	2.26
	DPV	−1.58	−1.57	0.78	0.56	−3.52	−5.66	2.14
**AzNI–3**	CV	−1.88	−1.55	0.95	0.81	−3.55	−5.91	2.36
	DPV	−1.63	−1.45	0.72	0.62	−3.66	−5.72	2.07
**AzNI–4**	CV	−1.59	−1.42	0.69	0.45	−3.68	−5.55	1.87
	DPV	−1.58	−1.33	0.49	0.31	−3.77	−5.41	1.62
**AzNI–6**	CV	−1.73	−1.56	0.74	0.64	−3.54	−5.74	2.20
	DPV	−1.73	−1.57	0.64	0.49	−3.52	−5.59	2.07

$E_{HOMO} = (-5.1-E_{ox}^{1(onset)})\cdot e^-$, $E_{LUMO} = (-5.1-E_{red}^{1(onset)})\cdot e^-$, $E_g = E_{ox}^{1(onset)}-E_{red}^{1(onset)}$. Solvent: dry ACN and 0.1 mol/dm^3 Bu_4NPF_6 and platinum wire as a working electrode. E_{ox}^{1} – the first oxidation process, E_{red}^{1} – the first reduction process, $E_{red}^{1(onset)}$ – the onset potential of the first reduction process, $E_{ox}^{1(onset)}$– the onset potential of the first oxidation process. E_{HOMO} and E_{LUMO} as IP and EA. v = 0.1 V/s for CV and v = 0.05V/s for DPV.

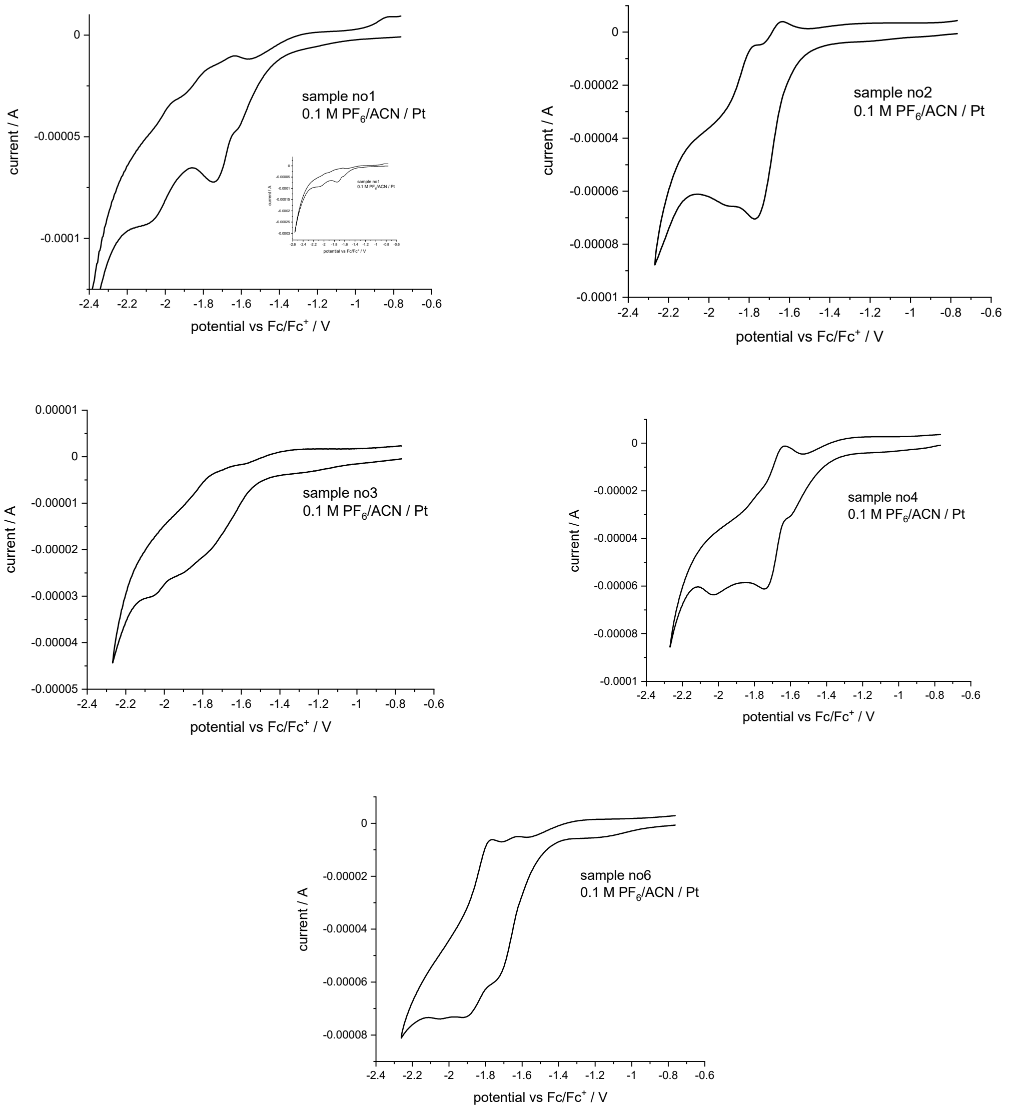

Figure S13. CV of the samples 1-6 (AzNI-1 – AzNI-6) (except AzNI-5, sample no5) in 0.1 M tetrabutylammonium hexafluorophosphate (Bu_4NPF_6) in dry acetonitrile (ACN) after Ar bubbling, at room temperature.

References

51. Gaussian 09, Revision A.02, Frisch M. J., Trucks G. W., Schlegel H. B., Scuseria G. E., Robb M. A., Cheeseman J. R., Scalmani G., Barone V., Petersson G. A., Nakatsuji H., Li X., Caricato M., Marenich A., Bloino J., Janesko B. G., Gomperts R., Mennucci B., Hratchian H. P., Ortiz J. V., Izmaylov A. F., Sonnenberg J. L., Williams-Young D., Ding F., Lipparini F., Egidi F., Goings J., Peng B., Petrone A., Henderson T., Ranasinghe D., Zakrzewski V. G., Gao J., Rega N., Zheng G., Liang W., Hada M., Ehara M., Toyota K., Fukuda R., Hasegawa J., Ishida M., Nakajima T., Honda Y., Kitao O., Nakai H., Vreven T., Throssell K., Montgomery J. A. Jr., Peralta J. E., Ogliaro F., Bearpark M., Heyd J. J., Brothers E., Kudin K. N., Staroverov V. N., Keith T., Kobayashi R., Normand J., Raghavachari K., Rendell A., Burant J. C., Iyengar S. S., Tomasi J., Cossi M., Millam J. M., Klene M., Adamo C., Cammi R., Ochterski J. W., Martin R. L., Morokuma K., Farkas O., Foresman J. B., and D. J. Fox, Gaussian, Inc., Wallingford CT, 2016
52. Becke, A. D. Density-functional thermochemistry. III. The role of exact exchange, *J. Chem. Phys.*, ***1993***, *98*, 5648-5652. https://doi.org/10.1063/1.464913.
53. Lee, C.; Yang, W.; Parr, R.G. Development of the Colle-Salvetti correlation-energy formula into a functional of the electron den sity, *Phys. Rev. B.**1988***, *37*, 785-789. https://doi.org/10.1103/PhysRevB.37.785.
54. Grimme, S.; Ehrlich, S.; Goerigk, L. Effect of the damping function in dispersion corrected density functional theory, *J. Comput. Chem.**2011***, *32*, 1456-1465. https://doi.org/10.1002/jcc.21759.
55. Barone, V.; Cossi, M. Quantum Calculation of Molecular Energies and Energy Gradients in Solution by a Conductor Solvent Model, *J. Phys. Chem. A.* ***1998***, *102*, 1995.https://doi.org/10.1021/jp9716997.
56. O'Boyle, N.M.; Tenderholt, A.L.; Langner, K.M. A Library for Package-Independent Computational Chemistry Algorithms. *J. Comput. Chem.**2008***, *29*, 839-845. https://doi.org/10.1002/jcc.20823.
57. Casida, M.E. In Recent Developments and Applications of Modern Density Functional Theory, Theoretical and Computational Chemistry, Jorge M. Seminario Eds.; Elsevier: Amsterdam, 1996; Volume 4, pp. 391.
